# Supplementary material for: Exploiting spatial dimensions to enable parallelized continuous directed evolution
Source: Mol Syst Biol. 2022 Sep 21;18(9):e10934. doi: 10.15252/msb.202210934 (PMC9491160; doi:10.15252/msb.202210934)
Supplement: Supplementary file 1 — Appendix [file MSB-18-e10934-s007.pdf]

Exploiting spatial dimensions to enable parallelized continuous directed evolution

Ting Wei[1,*], Wangsheng Lai[1,*], Qian Chen[1,2,*], Yi Zhang[1,*], Chenjian Sun[1,2], Xionglei He[3], Guoping Zhao[1,4], Xiongfei Fu[1,2,+], Chenli Liu[1,2,+]

[1]CAS Key Laboratory for Quantitative Engineering Biology, Shenzhen Institute of Synthetic Biology, Shenzhen Institutes of Advanced Technology, Chinese Academy of Sciences, Shenzhen 518055, China

[2]University of Chinese Academy of Sciences, Beijing 100049, China

[3]State Key Laboratory of Biocontrol, School of Life Sciences, Sun Yat-Sen University, Guangzhou, 510275, China

[4]Key Laboratory of Synthetic Biology, Institute of Plant Physiology and Ecology, Shanghai Institutes for Biological Sciences, Chinese Academy of Sciences, Shanghai, 200032, China

*equal contribution

[+]To whom correspondence may be addressed. Email: cl.liu@siat.ac.cn or xiongfei.fu@siat.ac.cn

Appendix

Appendix Text (page 3)

Appendix Text S1. Experimental notes
Calculation of phage generation time of SPACE system

Appendix Figures

Appendix Figure S1 (page 4). Growth curves of *E. coli* and phage production after infection.
Appendix Figure S2 (page 5). Model simulation of phage infection process.
Appendix Figure S3 (page 6). Time-lapse plots of the simulated relative bacterial cell-density and nutrient profiles along an arc line across the infected region with a fixed radius.
Appendix Figure S4 (page 7). Competition between weak and strong phages with an initial titer ratio of 10^5:1.
Appendix Figure S5 (page 8). Gating strategy used for flow cytometry analysis.
Appendix Figure S6 (page 9-12). Histograms obtained for the measurement of *in vivo* transcriptional activity based on flow cytometry.
Appendix Figure S7 (page 13). Enrichment of PS by SPACE system and liquid continuous culturing system by model simulation.
Appendix Figure S8 (page 14). Simulated competition of five phage strains with different bacterial expansion speed.
Appendix Figure S9 (page 15). Ratio of bacterial cells with fluorescent signals after infection by reporter phages.
Appendix Figure S10 (page 16). Simulated competition of two phages with a host bacterial population containing resistant cells.

Appendix Tables

Appendix Table S1 (page 17). Bacterial strains used in this study.
Appendix Table S2 (page 17-18). Model parameters.
Appendix Table S3 (page 18-20). Sequences of T7 promoter variants.
Appendix Table S4 (page 20-21). Plasmids used in this study.
Appendix Table S5 (page 21). Representative mutants obtained from parallelized SPACE of T7 RNAP

Appendix References (page 21)

Appendix Text S1. Experimental notes

Calculation of phage generation time of SPACE system

We estimated the phage generation time using the same model proposed for PACE (Esvelt *et al*, 2011). This model is based on an assumption that lagoons, in which the phage-assisted evolution takes place, operate in a steady state that the total phage and cell concentration does not change over time. The average phage generation time in this model is equal to 2 / (dilution rate) (Esvelt *et al.*, 2011). In SPACE, the bacterial propagating front composed of exponentially growing and steadily advancing cells provides a constant flow of fresh host. Once infected by the phages, bacterial cells are able to migrate and carrying along progeny phages. It is analogous to a lagoon. Those left behind the propagating front are considered as outflow of the lagoon. Therefore, the dilution rate in SPACE system should be equal to (cell expansion speed /front width). The cell expansion speed measured from time-lapsed images (**Fig 1C**) is approximately 3.89 mm/h, and the front width is 1.10-1.41 mm, so the dilution rate of SPACE under this condition is 2.76-3.54 h^{-1}, comparable with the dilution rate of PACE (2.5-3.2 h^{-1}) (Esvelt *et al.*, 2011). In the meantime, the effective duration of the evolution in a single run is calculated by dividing the distance from the phage inoculation spot (10 mm from the center) to the edge of the agar plate with cell expansion speed, which is 8.35 h for an 8.5-cm plate, and 16.71 h for a 15-cm plate. Thus, a single run using an 8.5-cm agar plate corresponds to approximately 12-15 phage generations. Longer effective duration provided by a 15-cm agar plate allows 23-30 generations in a single run over night. More phage generations could be achieved by simply transferring to a new agar plate and start another run.

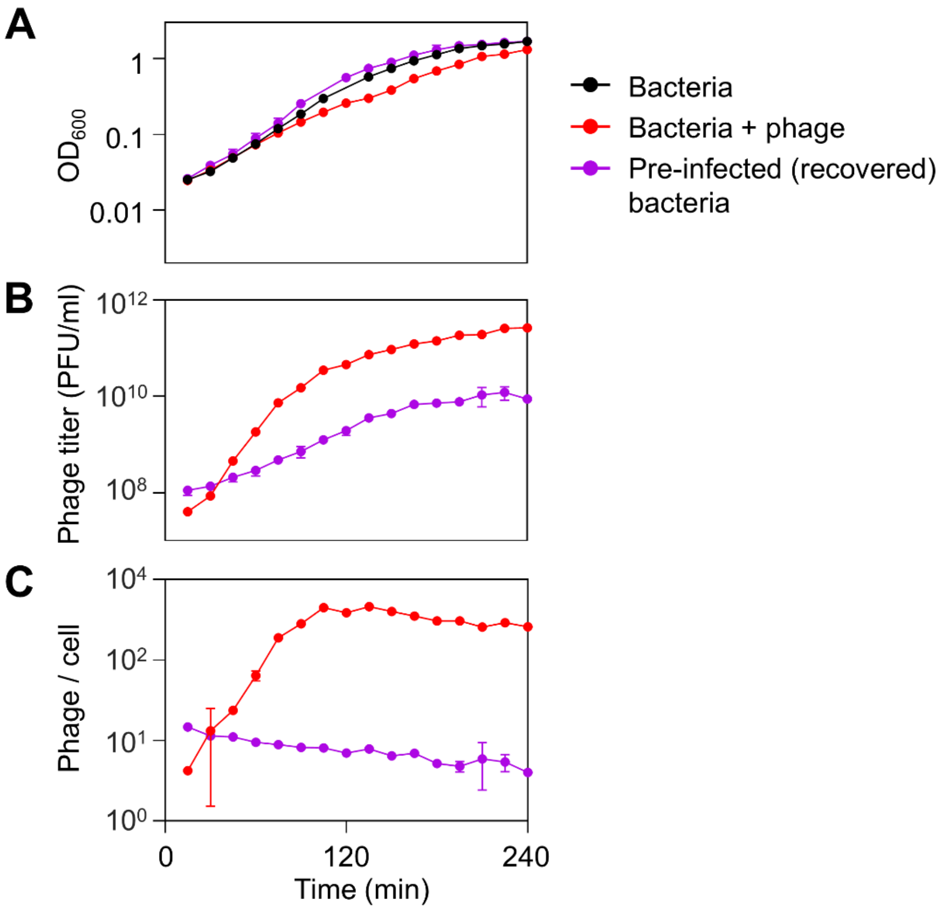

Appendix Figure S1 - Growth curves of *E. coli* and phage production after infection.

A Growth curves of uninfected, freshly infected, and pre-infected (recovered) bacteria. *E. coli* FM15 cells were cultured in LB broth. Bacteria are FM15 cells without phage inoculation; Bacteria + phage are cells with 10^8 PFU/ml of M13 phages added at the 0 time point. Pre-infected (recovered) bacteria are cells co-cultured overnight with phages, washed to remove phages in the supernatant, and transferred to fresh LB broth. For all three groups, the initial OD_{600} of the bacterial suspension was adjusted to 0.01.

B Phage titer in the supernatant of collected samples quantified by qPCR.

C Phage number per cell calculated by dividing phage titer with the cell density quantified by flow cytometry. Data represent mean values ± s.d. for three biological replicates.

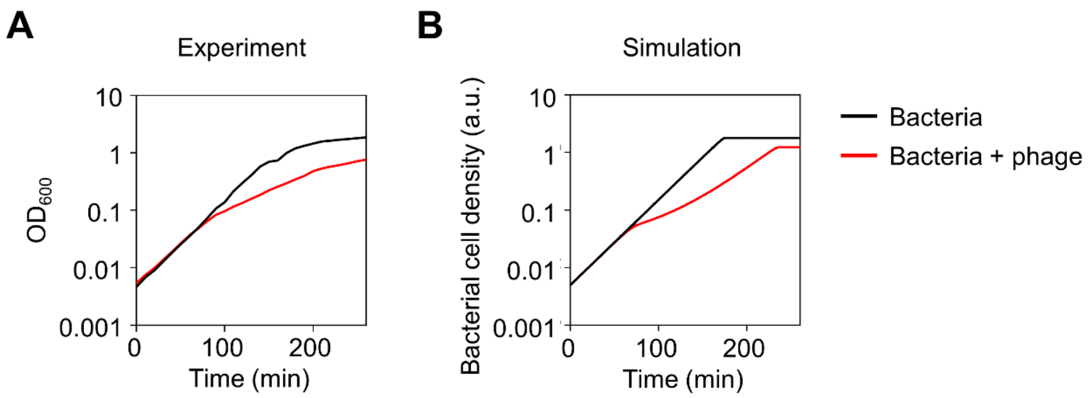

Appendix Figure S2 - **Model simulation of phage infection process.**

A Growth curves of *E. coli* FM15 cells in liquid culture without phage, or with 10^7 PFU/ml phages added at the beginning of the experiment.

B Model simulated bacterial growth curve without phage, or with an initial phage titer of 0.01 a.u. in a well-mixed system.

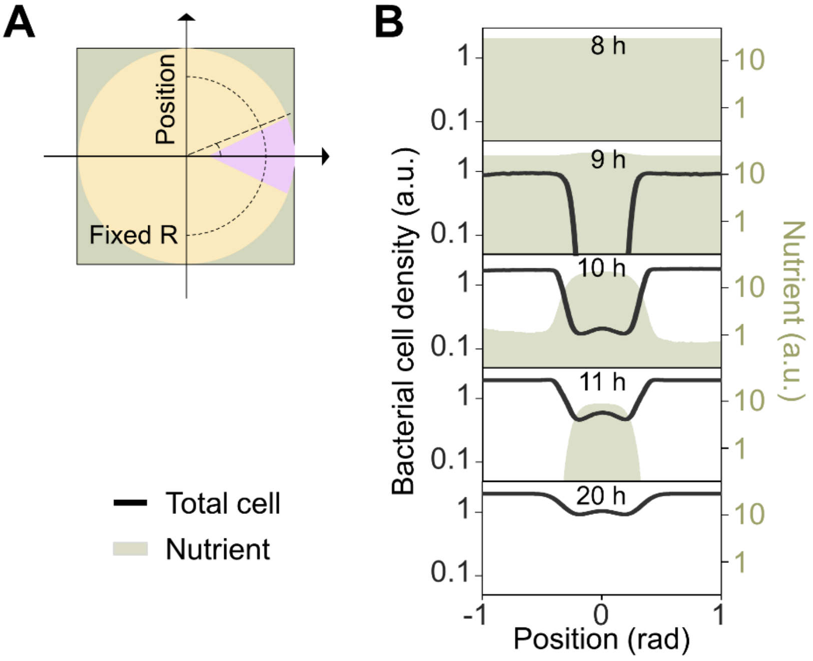

Appendix Figure S3 - **Time-lapse plots of the simulated relative bacterial cell-density and nutrient profiles along an arc line across the infected region with a fixed radius**.

A Schematic of a simulated fan shape formed after 20 h of bacterial range expansion. Yellow filled circle represents bacterial lawn. The fan-shaped infected zone is signified by purple color.

B Bacterial cell density and nutrient concentration values obtained from an arc line with a fixed radius across the fan shape as shown in (A). The model parameters used were identical to those used in the bottom two rows of Fig 1C. The fixed radius was set at 25 mm from the center of the agar plate.

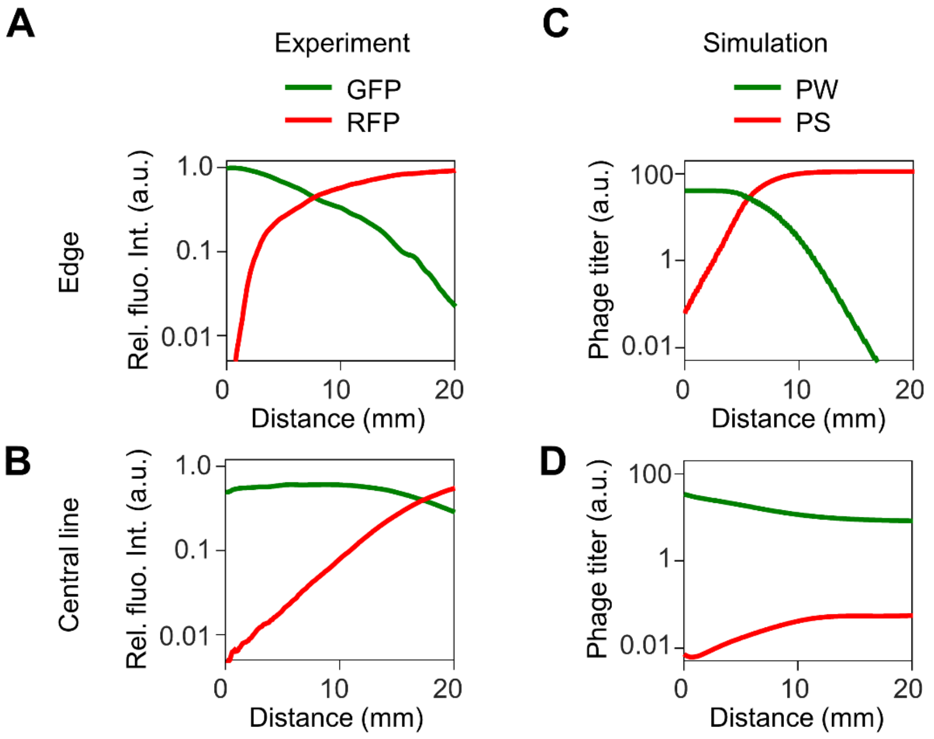

Appendix Figure S4 - Competition between weak and strong phages with an initial titer ratio of 10^5:1.

A, B Profiles of the fluorescence intensity along the central line and the edge of the fan-shaped infection zone in the experimental result in Fig 3C. The relative intensities were obtained by dividing the detected values with the maximum value of red or green fluorescence intensity, respectively.

C, D Simulated profiles of phage titer of the weak phage (PW) and the strong phage (PS) along the central line and the edge of the fan-shaped infection zone after competition as shown in Fig 3C.

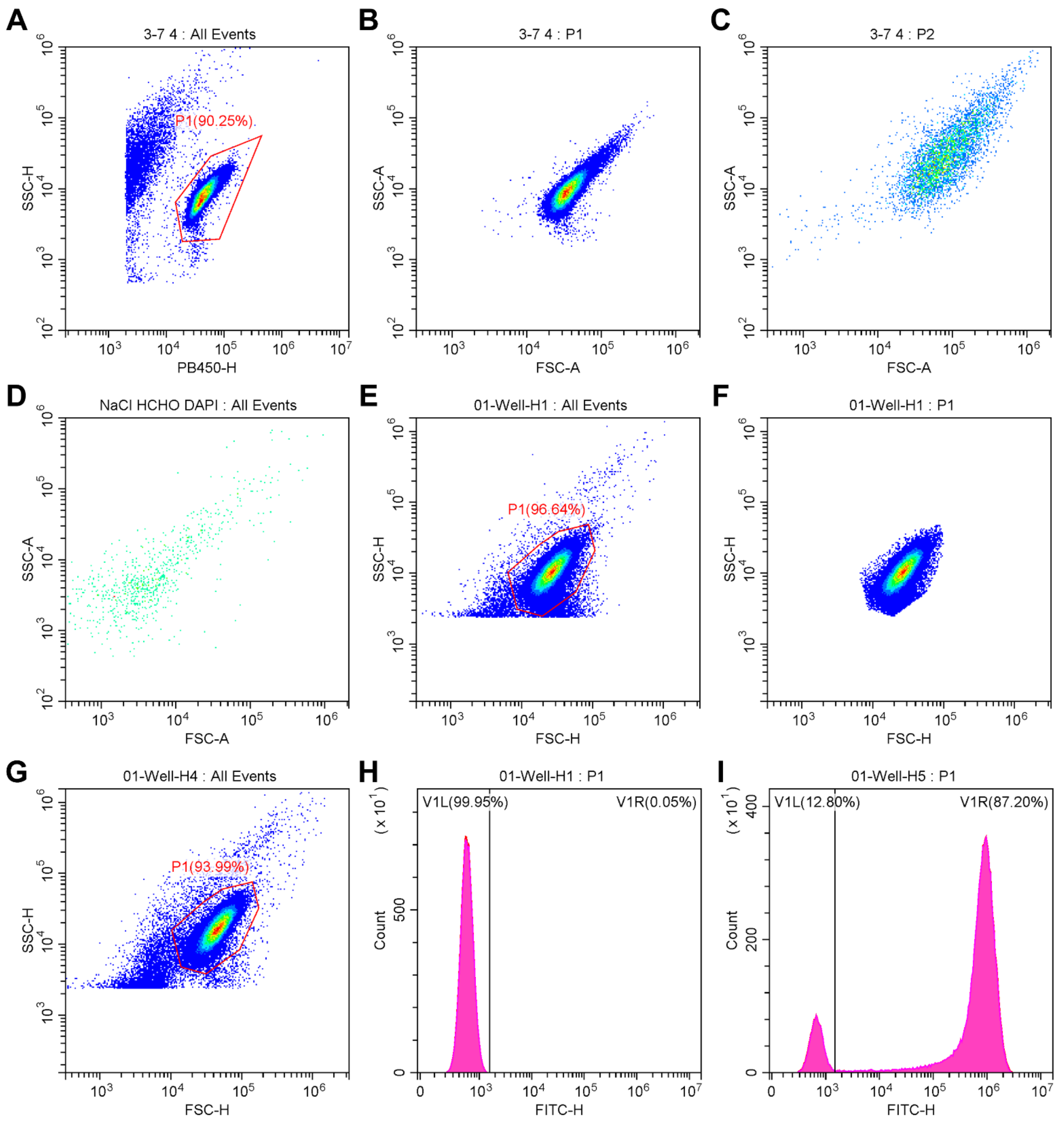

Appendix Figure S5 - Gating strategy used in flow cytometry analysis.

A-D Gating strategy used in cell counting. The DAPI-positive particles in P1 in panel (A) were regarded as bacterial cells. The FSC-H/SSC-H plots of populations inside and outside of P1 are shown in panels (B) and (C), respectively. The FSC-H/SSC-H plot of the staining buffer (0.9 % NaCl with 1 µg/ml DAPI) is shown in panel (D).

E-I Gating strategy used in the measurement of *in vivo* transcriptional activity. Particles in P1 in panel (E) were regarded as bacterial cells, and the population formed by these particles is shown in panel (F). The FSC-H/SSC-H plot of phage-infected bacterial cells is shown in panel (G). (H) For the measurement of *in vivo* transcriptional activity, V1L covering 99.95% of the population in an uninfected bacterial sample based on the distribution of FITC-H signal values was set to determine the background fluorescence. This gate was applied to phage-infected samples of the same bacterium, and particles falling in V1R as shown in panel (I) were used to measure the transcriptional activity.

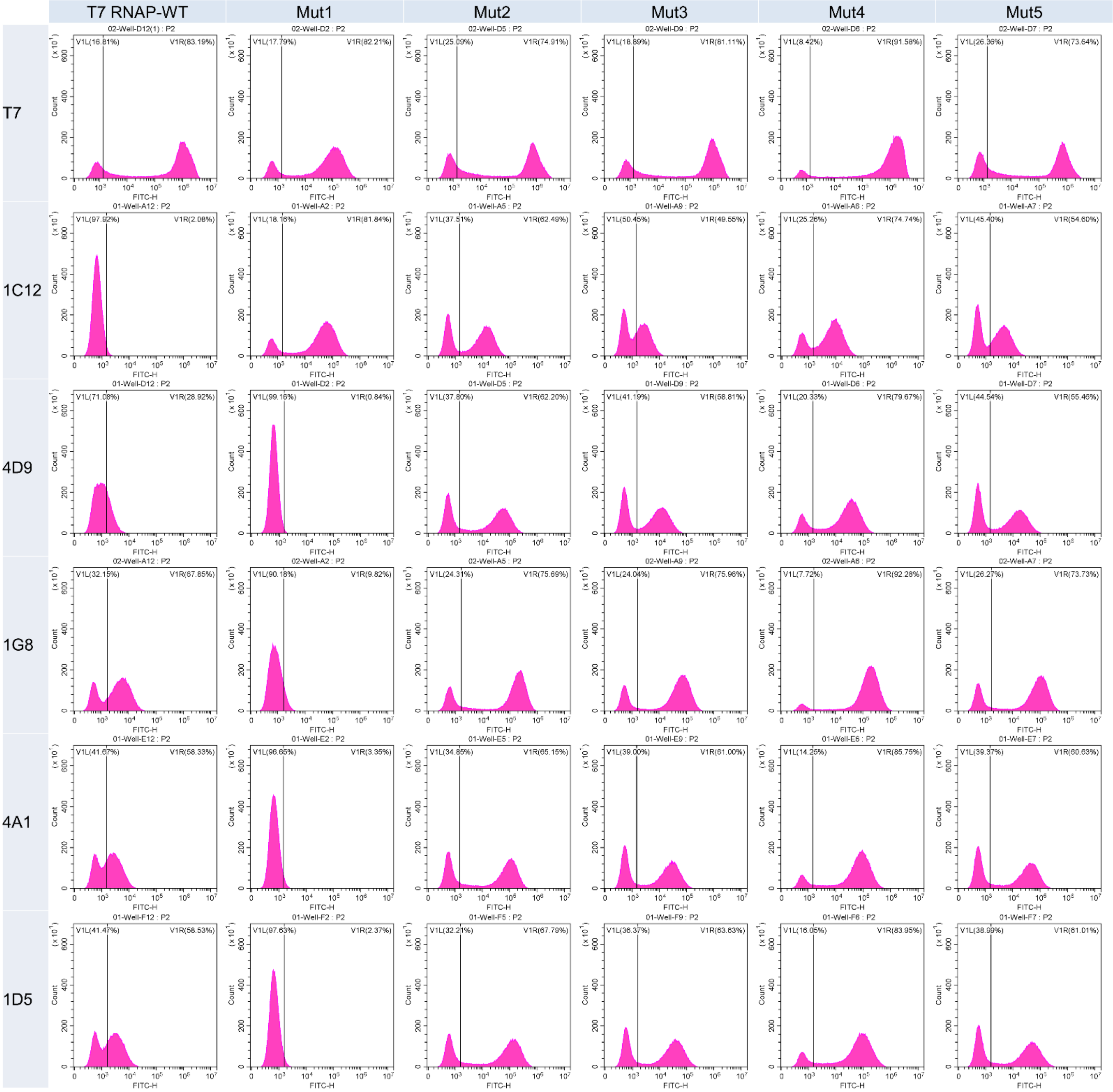
T7 RNAP-WT
Mut1
Mut2
Mut3
Mut4
Mut5
T7
1C12
4D9
1G8
4A1
1D5
Count
FITC-H

(Fig S6, Part 1)

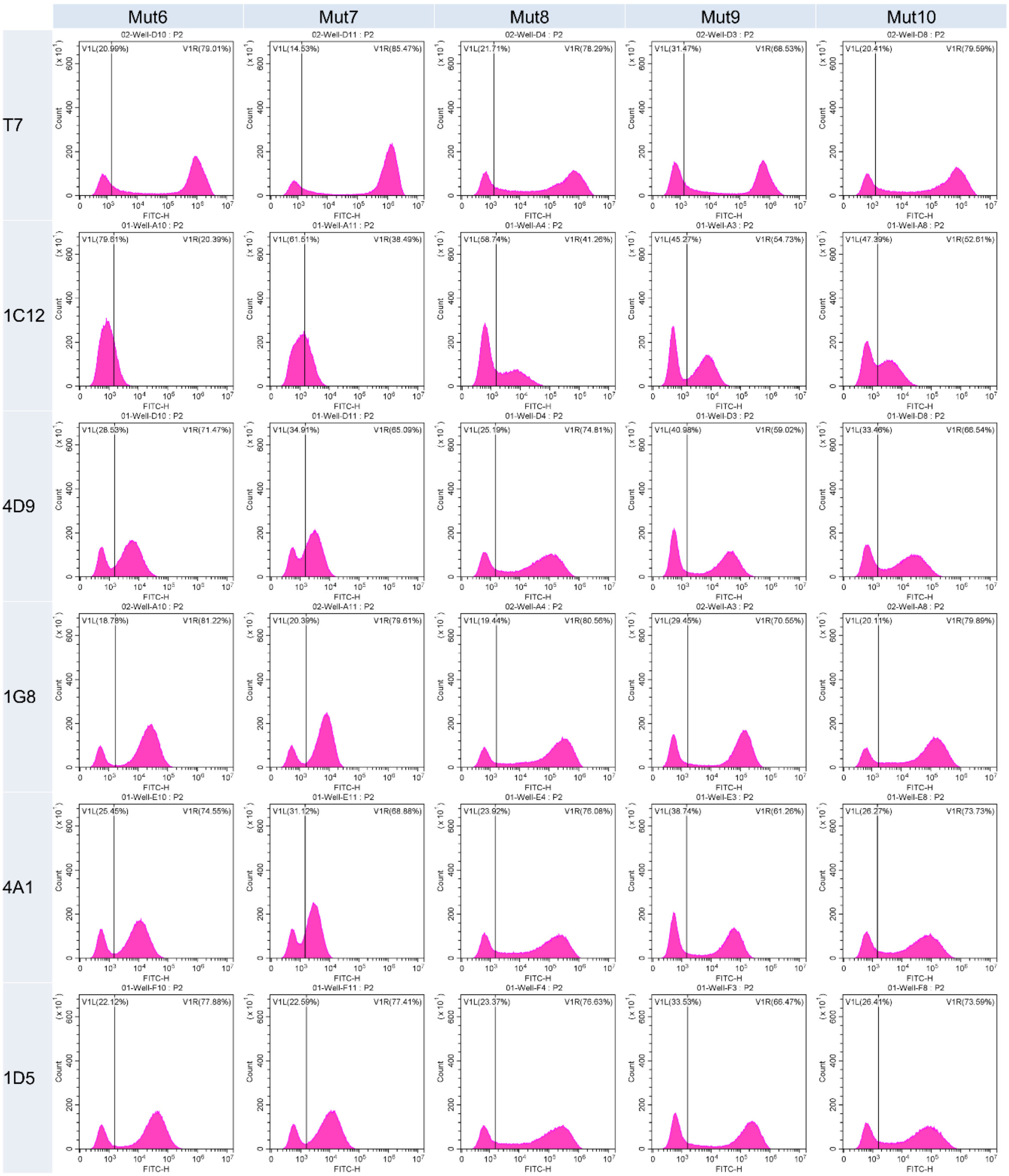

(Fig S6, Part 2)

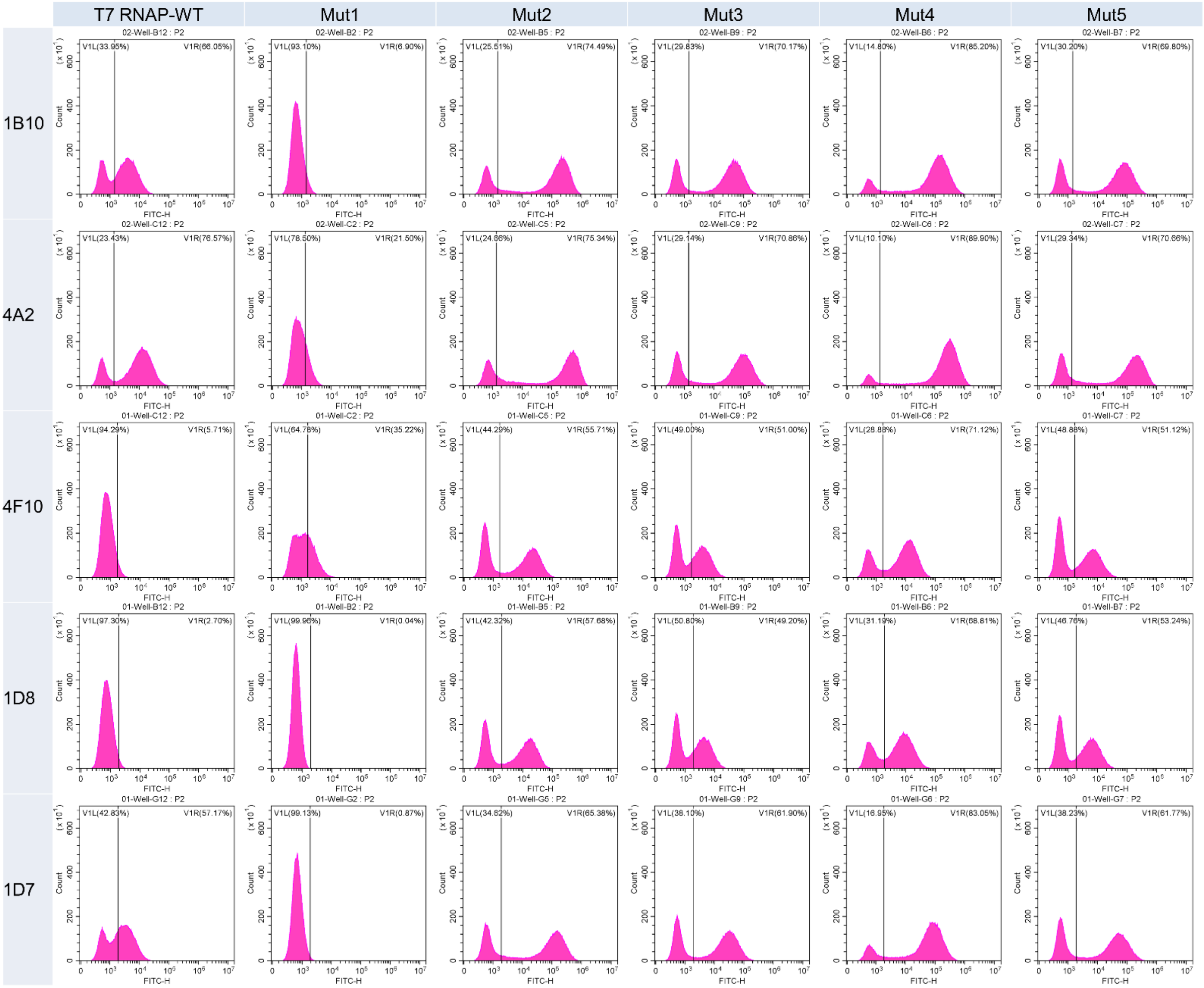
T7 RNAP-WT
Mut1
Mut2
Mut3
Mut4
Mut5
1B10
4A2
4F10
1D8
1D7
FITC-H
Count

(Fig S6, Part 3)

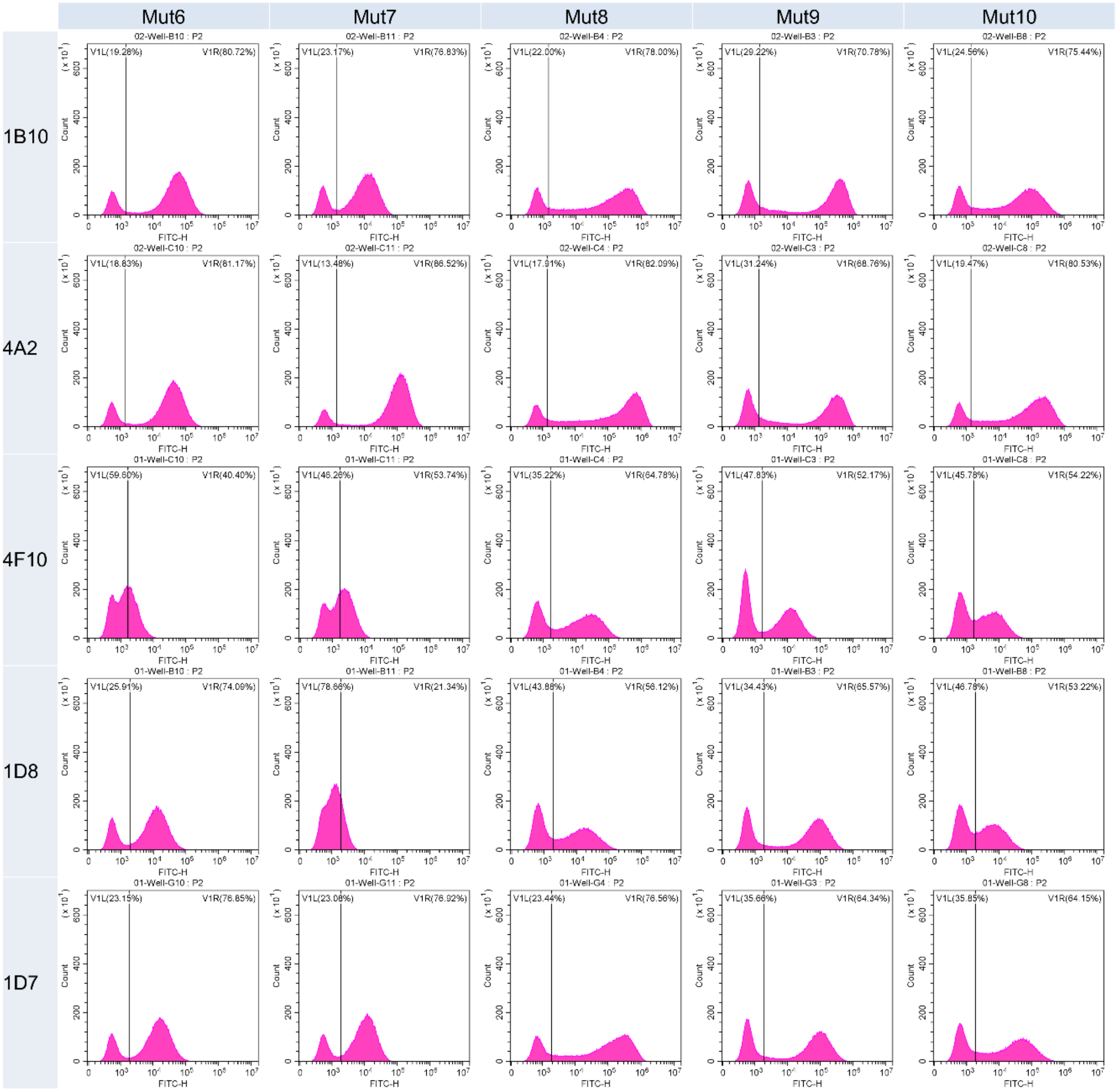

(Fig S6, Part 4)

Appendix Figure S6 - Histograms obtained for the measurement of *in vivo* transcriptional activity based on flow cytometry.

Results from one out of three replicated assays to measure the expression activity of the wild-type and 10 mutant RNAPs on T7 promoter and 10 promoter variants in **Fig 4D** are shown in this figure. Results from the other two replicates are included in the Source Data.

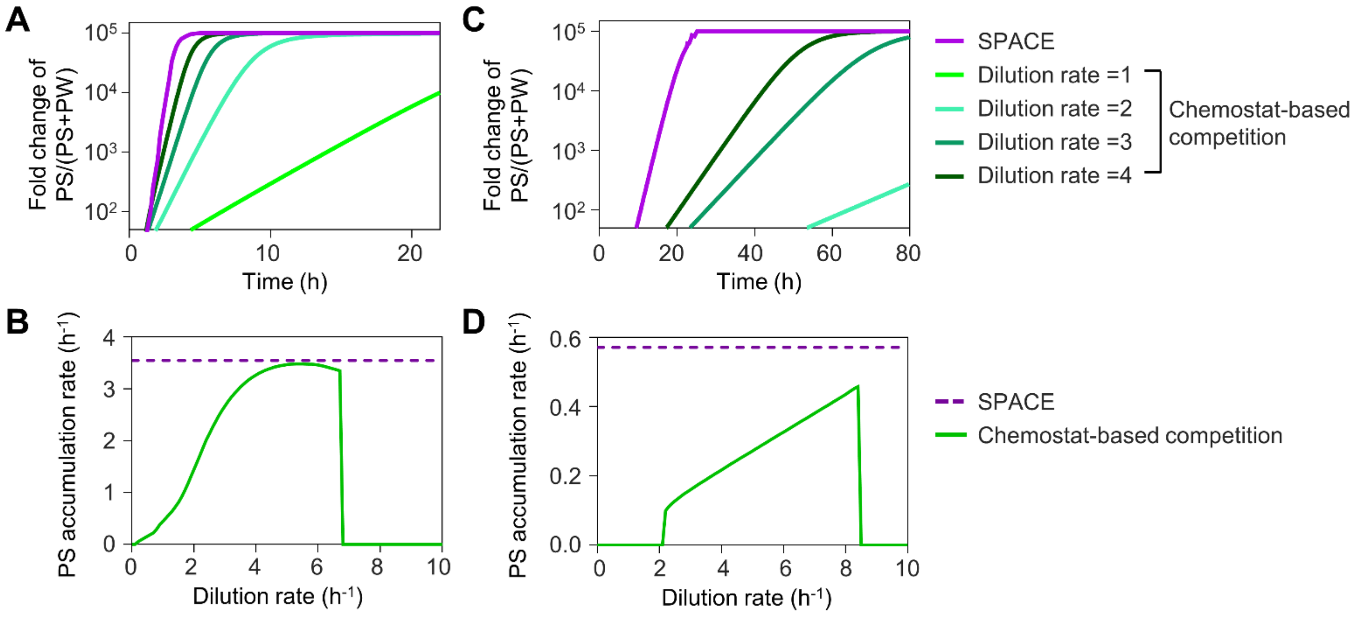

Appendix Figure S7 - Enrichment of PS by SPACE system and chemostat-based competition system by model simulation.

A, B Simulated fold change of PS/(PS+PW) and PS accumulation rate in SPACE and a chemostat-based competition system with a significant difference in the strength of PS and PW. Initial PW: PS is 10^5:1. Production rates of PW and PS are 40 and 100, respectively. Fold change along the sideward edge of infection zone under a SPACE condition the same as in **Fig 3** is compared with that in well-mixed chemostat-based competition system using different dilution rate (DR, volumes of the liquid system exchanged by continuous flow per hour). Data for liquid system in (B) are obtained by calculating the slope of curves on logarithmic scale with varied DR as shown in (A).

C, D Simulated fold change of PS/(PS+PW) and PS accumulation rate in SPACE and a chemostat-based competition system with a mild difference in the strength of PS and PW. Initial PW: PS is 10^5:1. Production rates of PW and PS are 90 and 100, respectively.

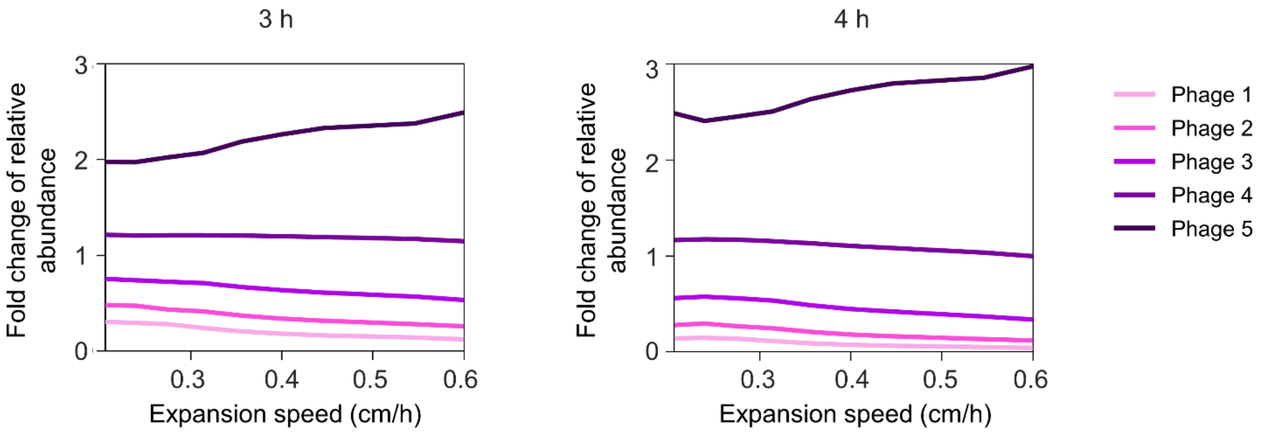

Appendix Figure S8 - Simulated competition of five phage strains with different bacterial expansion speed. Production rates of Phage 1-5 are 80, 85, 90, 95, and 100, respective. The initial mixture of phage inoculant consists of the five strains, each composing 20% of the whole population. Graphs show the fold change of the relative abundance of each phage at the bacterial expanding front near the edge as compared to that at the initial phage spot 3 h and 4 h after phage infection.

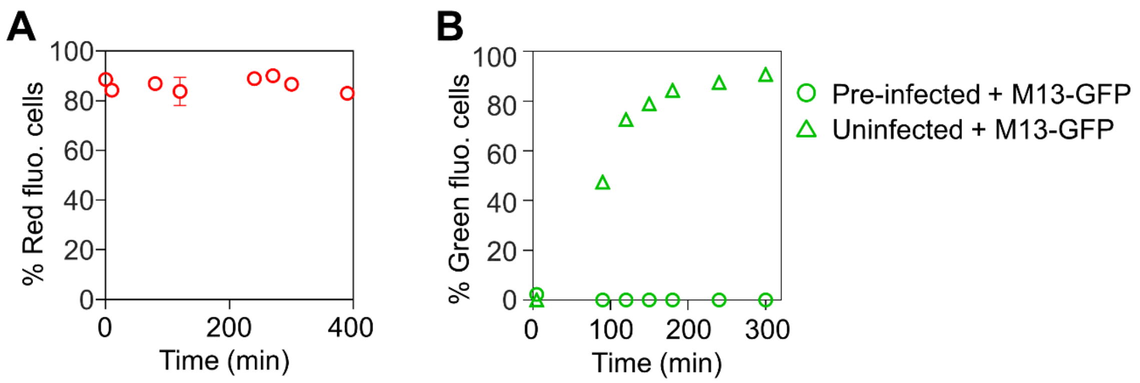

Appendix Figure S9 - Ratio of bacterial cells with fluorescent signals after infection by reporter phages.
A Time course of the percentage of cells exhibiting red fluorescence signals after overnight pre-infection by marker phages. *E. coli* FM15 cells in LB broth were infected by M13s used in **Fig 3**, the reporter M13 phage with a red fluorescent protein gene in its genome, for 10 hours. These pre-infected cells were washed to remove supernatant phages, and 1:500 transferred to fresh LB broth to continue culturing with fluorescence measurements at different time points.
B Time course of the percentage of cells exhibiting green fluorescence signals after M13-GFP infection of pre-infected and uninfected fresh bacterial cells. Pre-infected cells were prepared as in (A).
The ratio of fluorescent cells was measured by flow cytometry using PE and FITC channels for red and green fluorescence, respectively. Data represent mean values ± s.d. for three biological replicates.

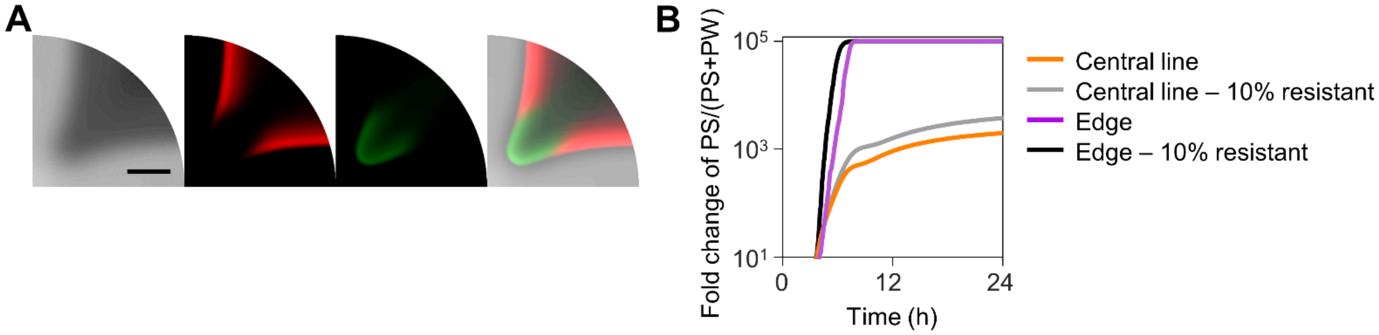

Appendix Figure S10 - Simulated competition of two phages with a host bacterial population containing resistant cells.

A, B The outcome and fold enrichment of the strong phage in simulated competition assay with an initial of weak phage (PW): strong phage (PS) at 10^5:1. The production rates of PW and PS are 40 and 100, respectively. In the host bacterial population, 10% of cells resistant to phage infection all the time was assumed for this simulation. In panel (B), the fold enrichment along the central radial line and sideward edge of infection zone is compared between two cases: bacterial population containing 0%, or 10% of cells resistant to phage infection.

Appendix Table S1. Bacterial strains used in this study.

Strain name	Genotype and remarks	Reference or source
E. coli MG1655-mCherry	*F⁻ λ– ilvG– rfb-50 rph-1 attB::mCherry (AmpR)*	Ref. (Liu *et al*, 2019)
E. coli ER2738	*F' proA+B+ lacI^q Δ(lacZ)M15 zzf::Tn10 (TetR)/fhuA2 glnV Δ(lac-proAB) thi-1 Δ(hsdS-mcrB)5*	NEB
E. coli DH5α	*F⁻ φ80d lacZΔM15 Δ(lacZYA-argF) U169 endA1 recA1 hsdR17(r_k^-, m_k^+) supE44λ- thi-1 gyrA96 relA1 phoA*	TransGen Biotech, Beijing
E. coli BL21	*F⁻ dcm omp*T *hsd*S(r_B^- m_B^-) *gal* [*mal*B$^+$]$_{K-12}$(λ^S)	TransGen Biotech, Beijing
E. coli FMG1655-mCherry	*F' proA+B+ lacI^q Δ(lacZ)M15 zzf::Tn10 (TetR)/ λ– ilvG– rfb-50 rph-1 attB::mCherry* (Amp^R*)*	This study
E. coli FMG1655-mCherry *Δ(lacZ)M15*	*F' proA+B+ lacI^q Δ(lacZ)M15 zzf::Tn10 (TetR)/ λ– ilvG– rfb-50 rph-1 attB::mCherry* (Amp^R*) Δ(lacZ)M15*	This study
E. coli FM15	*F' proA+B+ lacI^q Δ(lacZ)M15 zzf::Tn10 (TetR)/ λ– ilvG– rfb-50 rph-1 attB::Kan^R Δ(lacZ)M15*	This study

Appendix Table S2. Model parameter

Parameter	Symbol	Value	Reference or parameter variation
Effective diffusion coefficient	μ	60 $\mu m^2/s$	Ref. (Liu *et al.*, 2019)
Chemotactic coefficient	χ	410 $\mu m^2/s$	Fit to match expansion speed for expansion in reference condition.
Diffusion of nutrient	D_n	800 $\mu m^2/s$	Ref. (Cremer *et al*, 2016)
Diffusion of attractant	D_s	800 $\mu m^2/s$	Ref. (Cremer *et al.*, 2016)
Growth rate	λ_0	2.08 h^{-1}	Average growth rate in LB medium. **Appendix Figure S2**
Yield nutrient consumption	Y_n	0.064 OD_{600}/mM	Ref. (Liu *et al.*, 2019)
Monod constant nutrient uptake	n_k	0.4 mM	Defined by fitting the growth curve of *E. coli*. **Appendix Figure S2**
Lower Weber offset attractant sensing	K_1	3.5 μM	Ref. (Yang *et al*, 2015)
Upper Weber offset attractant sensing	K_2	1000 μM	Ref. (Yang *et al.*, 2015)
Uptake rate chemoattractant	g_0	9 μM/(min • OD_{600})	Ref. (Fu *et al*, 2018)
Monod constant attractant uptake	s_k	1 μM	Ref. (Schellenberg & Furlong, 1977)
Phage production rate	α	0.1~100 a.u. /OD_{600}	Production rate varied to match the resultant fan shape size at different levels of *gIII* expression

Infection ability of phage	k_0	0.9 a.u. $^{-1}$	Fit to match the growth curve of *E. coli* infected by M13 bacteriophage. **Appendix Figure S2**
Monod constant phage uptake	p_k	0.01 a.u.	Fit to match the growth curve of *E. coli* infected by M13 bacteriophage. **Appendix Figure S2**
Growth coefficient of infected cell	η_1	0.1	Fit to match the growth curve of *E. coli* infected by M13 bacteriophage. **Appendix Figure S2**
Growth coefficient of recovered cell	β_1	0.9	Defined by fitting the growth curve of *E. coli* infected by M13 bacteriophage. **Appendix Figure S2**
Recovered coefficient of infected cell	θ_1	10^{-4} s^{-1}	Fit to match the growth curve of *E. coli* infected by M13 bacteriophage. **Appendix Figure S2**
Initial concentration of nutrients	n_0	30 mM	
Initial concentration of attractant	s_0	60 µM	

Appendix Table S3. Sequences of T7 promoter variants.

Number	Sequence
1A1	ACCACAGTGAACCTACAGGGAGA
1A2	TAATACGAGTCCCTACAGGGAGA
1A3	TCATACGAGTACCTACAGGGAGA
1A4	ATCGGAGTAAACGAGAAGGGAGA
1A5	AACTACTCCTTTCTAGCGGGAGA
1A6	GTTCGGGATTATGAGCCGGGAGA
1A7	GAATGCGACTCACTATAGGGAGA
1A8	TAATCCGACACACCATAGGGAGA
1A9	GGAGTATGATCTCCAAAGGGAGA
1A10	ACGCAGAACAGTACTAGGGGAGA
1A11	CCTAGTCTTTACGAGATGGGAGA
1A12	AAGTACCCTTTACCCTTGGGAGA
1B1	TAAGACGAAACACGATTGGGAGA
1B2	TAATACGACGCACGATAGGGAGA
1B3	TAATACGTAGGACGATAGGGAGA
1B4	TAATACGTAGGACGAAAGGGAGA
1B5	TAATAAGCCTCTCATCGGGGAGA
1B6	AAACGCCACTCACTGTAGGGAGA
1B7	AAGCGGCACTCAATGAAGGGAGA
1B8	TAATCCCTCCTACAATAGGGAGA
1B9	GGGAGAATTAACGGAAGGGGAGA
1B10	GAATGCAACTCAGGAAAGGGAGA
1B11	AGTAAACCTCGTTACACGGGAGA

1B12	GTTAGAGCAACCATTCTGGGAGA
1C7	TAGTACGCCCCACTATAGGGAGA
1C8	ACATACGATTCAAGCGCGGGAGA
1C9	AGGACTCTGCAGTACCTGGGAGA
1C10	TTCGACGTCCCACAAAGGGGAGA
1C11	ATGAAGCGTCCTGATCAGGGAGA
1C12	TAATACGACCCACTTCAGGGAGA
1D1	CTAGAATTCGGTTGACAGGGAGA
1D2	CAAGACTTCTGACGATAGGGAGA
1D3	TGAAGCAGCTTCGTACAGGGAGA
1D4	CGGGGTATTAAGTAGGCGGGAGA
1D5	TAGTGCGACTCACAATAGGGAGA
1D6	TGGTGCGACTAACAATAGGGAGA
1D7	TGTAAGAACTCACTTTGGGGAGA
1D8	TGTATGAACTCACGTTGGGGAGA
1D9	GCTGACGACTGACTATTGGGAGA
1D10	GTATAAGTATTCGCGCAGGGAGA
1D11	TTAGCCACTTGGAACTAGGGAGA
1D12	AAATACGACTCACTATGGGGAGA
1E1	GAAGACGTCTCACTATAGGGAGA
1E2	GCTAGGATTACGCTATAGGGAGA
1E3	GCTAGGATTACGCTATCGGGAGA
1E4	GCTAGGATTAAGATAACGGGAGA
1E5	TTCCACTTTGCCCGTTTGGGAGA
1E6	TTCCGATTTGACCGTTTGGGAGA
1E7	TAACACTTCTCACGATAGGGAGA
1E8	CAATGCTCCTCACTAAAGGGAGA
1E9	GAATACGACTTACTAGGGGGAGA
1E10	GAATCCGACTTACTAGGGGGAGA
1E11	TCACACGACACCCTATAGGGAGA
1E12	ATCTCCGGTCATACGATGGGAGA
1G1	TCTACGGAATGTCCATTGGGAGA
1G2	ATGATTCCGCGGTAGCAGGGAGA
1G3	TAGTACGCGTGACTATAGGGAGA
1G4	ATTCTGTTCGTGATTCGGGGAGA
1G5	CTTCGTGTCGGATTAAGGGGAGA
1G6	TGTACCCCTTAAACTCTGGGAGA
1G7	TGTACCCCTGAAACTCTGGGAGA
1G8	TAATACGCCTCACCACAGGGAGA
1G9	AATTGAACCTCACCGTGGGGAGA
1G10	CGTGTATGTGATACTGCGGGAGA
4A1	TAATACGCCTCACCACTGGGAGA
4A2	GAATGCGACTCCCTATAGGGAGA
4A3	AAATACTAACGATTATAGGGAGA
4A4	TAGGACGTAAGCCGAATGGGAGA
4A5	GCTAGGATTAAGCTAACGGGAGA
4A6	GTTTACGATTCTGAACCGGGAGA

4D7	AAGTACCCCTTACCCTTGGGAGA
4D8	AAGCAGAACTCTCCAAAGGGAGA
4D9	AAATAAGCCTCACCGTAGGGAGA
4D10	AACGACGACTCCGTGAAGGGAGA
4D11	ATGCAGTGGCTTAGAGTGGGAGA
4D12	TAATACGCGTCACTATAGGGAGA
4E1	TACTACGCCTCTCTAGCGGGAGA
4E2	AATCCAGATTGACCACTGGGAGA
4E3	GGCACGATAATGTGCGGGGGAGA
4E4	TCAAACGACTTACCGTAGGGAGA
4E5	CTAGCCACTGGGAACTAGGGAGA
4E6	AAGTTCACCCCTTTGATGGGAGA
4F1	GATTACGATTCTGAACCGGGAGA
4F2	TAAAGTCGATCAGTACTGGGAGA
4F3	ATGCCGATAGATGACCTGGGAGA
4F4	TAAAGTGGATCACTACTGGGAGA
4F5	AGTACTCCTGACACTCTGGGAGA
4F6	TAATGCGCCTGACAATAGGGAGA
4F7	TATGACGACTCACTATAGGGAGA
4F8	AGAGATCACGCAATCCAGGGAGA
4F9	ACTAAGAGCATCGGTAAGGGAGA
4F10	TAAAGTGGCTCACTATTGGGAGA
4F11	ATTCTCTTCGCGATTCGGGGAGA
4F12	CTAGCTTACGGTACTTAGGGAGA
4G1	GTATAAGACTCACCGCAGGGAGA
4G2	ATGATTCCGCGATAGTAGGGAGA

Appendix Table S4. Plasmids used in this study.

Plasmid name	Class	Antibiotic resistance	Origin of replication	Promoter	Genes
pLAa1~96	AP[a]	Spe	pUC	P_{T7} and variants	gIII
pLAasc1~21	AP	Carb	SC101	P_{T7} and variants	gIII
pLAa188a	AP	Carb	SC101	P_{psp}	gIII
pLM1	MP-s[b]	Chl	CloDF13	P_{psp}	pspABCDE-dnaQ926-dam-seqA
pLRp	RP[c]	Chl	CloDF13	P_{psp}	pspABCDE-sfGFP
pLAR1	ARP[d]	Spe	ColEI	P_{psp}	pspABCDE
pLAr1~21	RP	Carb	SC101	P_{T7} and variants	sfGFP
SP-T7	SP[e]		f1	P_{gIII}	T7 RNAP WT
SP-T7mut	SP		f1	P_{gIII}	T7 RNAP mutant

SP-T7GFP	SP	f1	P_{gIII}/J23100	T7 RNAP WT/sfGFP
SP-T7mutRuby	SP	f1	P_{gIII}/J23100	T7 RNAP mutant/mRuby

[a] accessory plasmid; [b] mutagenesis plasmid using psp promoter to drive mutator genes; [c] reporter plasmid; [d] accessory plasmid harboring regulator genes of psp promoter; [e] selection phage

Appendix Table S5. Representative mutants obtained from parallelized SPACE of T7 RNAP

Mutant no.	Amino acid changes
**Mut1**	E222G Q758R H772R
**Mut2**	E222K
**Mut3**	H772R
**Mut4**	E222A
**Mut5**	E222G
**Mut6**	K206R I244V
**Mut7**	E242K
**Mut8**	E222V D770N
**Mut9**	K206R E222K
**Mut10**	I244V

Appendix References

Cremer J, Segota I, Yang C-y, Arnoldini M, Sauls JT, Zhang Z, Gutierrez E, Groisman A, Hwa T (2016) Effect of flow and peristaltic mixing on bacterial growth in a gut-like channel. *Proceedings of the National Academy of Sciences* 113: 11414

Esvelt KM, Carlson JC, Liu DR (2011) A system for the continuous directed evolution of biomolecules. *Nature* 472: 499-503

Fu X, Kato S, Long J, Mattingly HH, He C, Vural DC, Zucker SW, Emonet T (2018) Spatial self-organization resolves conflicts between individuality and collective migration. *Nature communications* 9: 2177

Liu W, Cremer J, Li D, Hwa T, Liu C (2019) An evolutionarily stable strategy to colonize spatially extended habitats. *Nature* 575: 664-668

Schellenberg GD, Furlong CE (1977) Resolution of the multiplicity of the glutamate and aspartate transport systems of Escherichia coli. *The Journal of biological chemistry* 252: 9055-9064

Yang Y, M Pollard A, Höfler C, Poschet G, Wirtz M, Hell R, Sourjik V (2015) Relation between chemotaxis and consumption of amino acids in bacteria. *Molecular microbiology* 96: 1272-1282
