# Supplementary material for: Exploiting spatial dimensions to enable parallelized continuous directed evolution
Source: Mol Syst Biol. 2022 Sep 21;18(9):e10934. doi: 10.15252/msb.202210934 (PMC9491160; doi:10.15252/msb.202210934)
Supplement: Supplementary file 2 — Expanded View Figures PDF [file MSB-18-e10934-s005.pdf]

Expanded View Figures

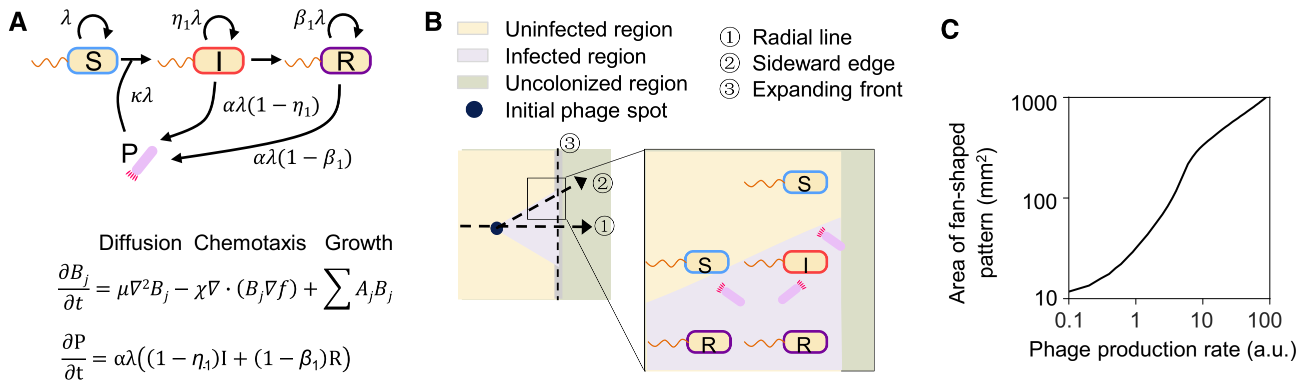

Figure EV1. Kinetic model of the interaction between bacteria and phage.

A The bacterial populations are classified into three categories: susceptible, infected, and recovered bacteria. Infected bacteria are converted from susceptible bacteria by phage infection, and eventually become recovered bacteria. These three bacterial populations all proliferate by consuming the nutrition in the semisolid culture media. In our experimental system, motile bacteria expand their range into unoccupied territories by diffusion and chemotaxis. In the meantime, the nonmotile phages are transmitted by their host bacteria, and their titer depends on the level of infectious progeny phage production by infected and recovered bacteria. The details of this model are described in Materials and Methods and Appendix Table S2.

B The spatiotemporal dynamics of phage infection in the radial direction (①) and the lateral direction (②) of the fan-shaped infection region are distinct from each other. In the radial direction, the expansion of infection range is driven by a "hitchhiking effect" (Ping *et al*, 2020), that is, phages are transported by the bacteria at the moving front. The bacterial population at the front experiences an active infection process involving the emergence of infected and recovered cells, the annihilation of susceptible cells, and eventually all cells become recovered cells, maintaining a balance between cell growth at the front and back diffusion (Cremer *et al*, 2019) (③). Differently, along the sideward edge, the expansion of infection range is driven by a "relay effect" of infected bacteria in the lateral direction, that is, infected bacteria from the infected region invade into the uninfected region, in which they produce phages continuously encountering and infecting susceptible bacterial cells. This cycle repeats and shows a relay-like effect, effectively generating a moving boundary between the infected and uninfected regions, which is eventually presented as the sideward edge (②).

C Model prediction showing that the size of the fan-shaped pattern is positively correlated with the phage production rate.

Source data are available online for this figure.

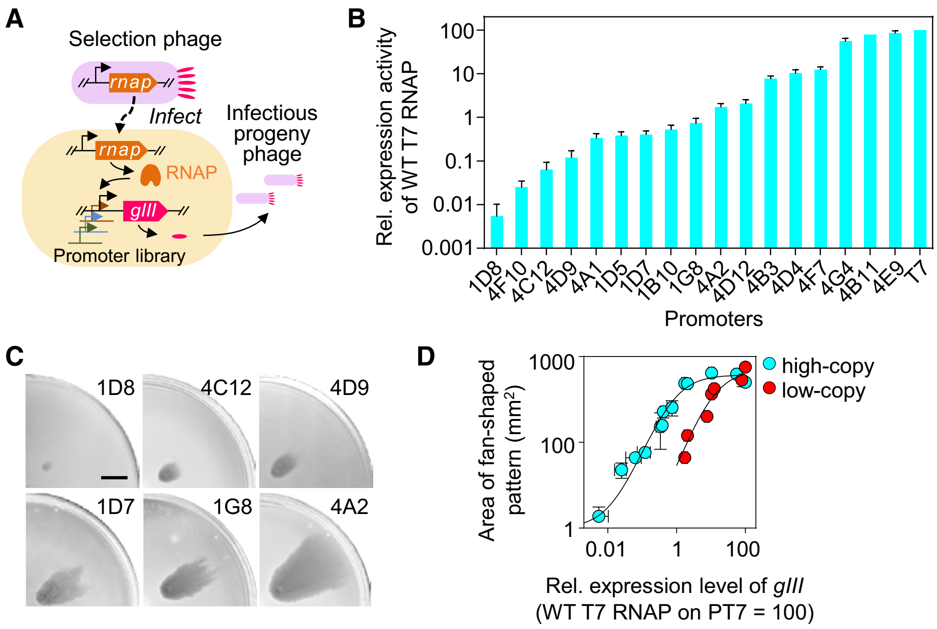

Figure EV2. Relationship between the area of the fan-shaped pattern and the *gIII* expression level.

A An activity-dependent phage propagation cassette on accessory plasmid. Expression level of *gIII* is under the control of a library of synthetic promoter variants. Selection phage carries a wild-type T7 RNA polymerase (RNAP) gene in place of its *gIII*. The T7 RNAP exhibits different transcriptional activities on different promoter variants.

B Relative expression levels of 17 synthetic promoter variants (sequences shown in Appendix Table S3) were determined by *in vivo* transcriptional activity assay (Materials and Methods). Error bars represent s.d. of three biologically independent assays.

C Photographs of a quarter of the semisolid agar plates with typical patterns obtained for bacteria carrying accessory plasmids containing representative promoters with different expression activities in (B). Scale bar represents 1 cm.

D Relationship between the area of the fan-shaped pattern and the *gIII* expression level. Data represent mean ± s.d. for at least three biological replicates. Fitting lines are generated with functions $y = 10 + 600x/(0.8 + x)$ and $y = 700x/(12 + x)$ for high- and low-copy accessory plasmids, respectively.

Source data are available online for this figure.

 © 2022 The Authors

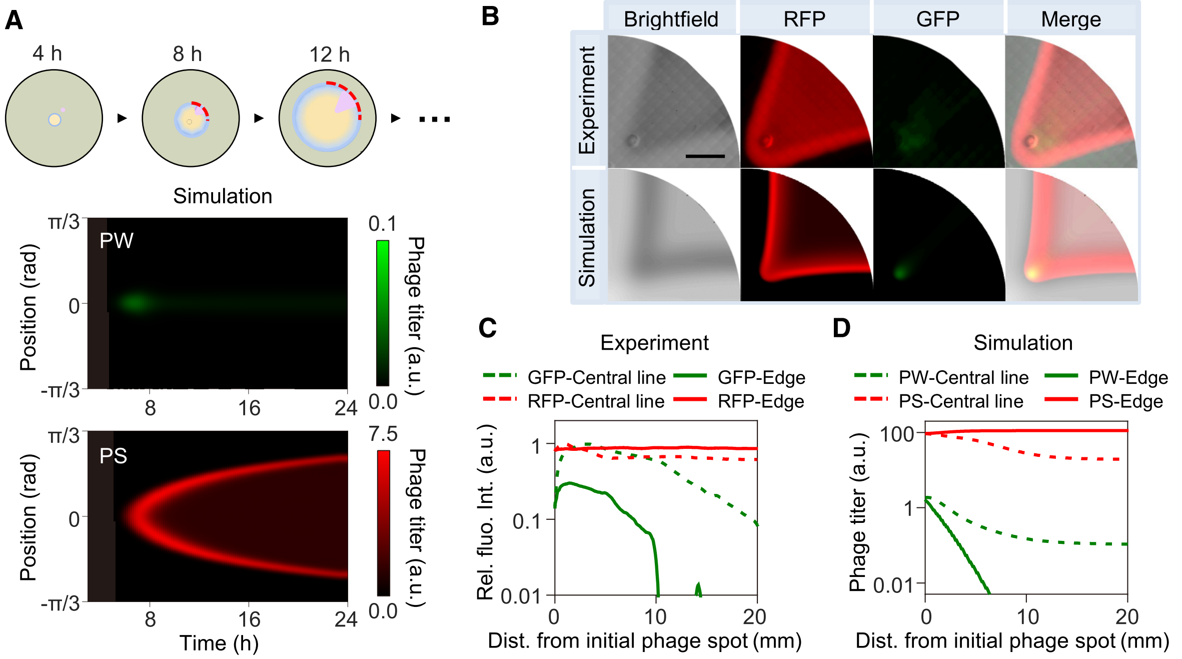

Figure EV3. Competition between weak and strong phages with an initial titer ratio of 1:1.

A Simulated kymograph of weak (PW) and strong (PS) phage titers in the bacterial expanding front as shown by the red dashed arc line in the schematic with an initial titer ratio of 1:1. The production rates of PW and PS are set as 40 and 100, respectively.

B The experimental result shows raw photographs of a representative two-phage competition assay after initial inoculation with 1:1 mixture of M13s and M13w at 1 cm away from the center. The simulation result is the outcome of competition between two phages with relative production rates as those in panel (A). Scale bar represents 1 cm.

C Profiles of the fluorescence intensity along the central radial line and the sideward edge of the fan-shaped infection zone in the experimental result in (B). The relative intensities were obtained by dividing the detected values with the maximum value of red or green fluorescence intensity, respectively.

D Plots of the simulated phage-titer profiles of PW and PS after 24-h competition.

Source data are available online for this figure.

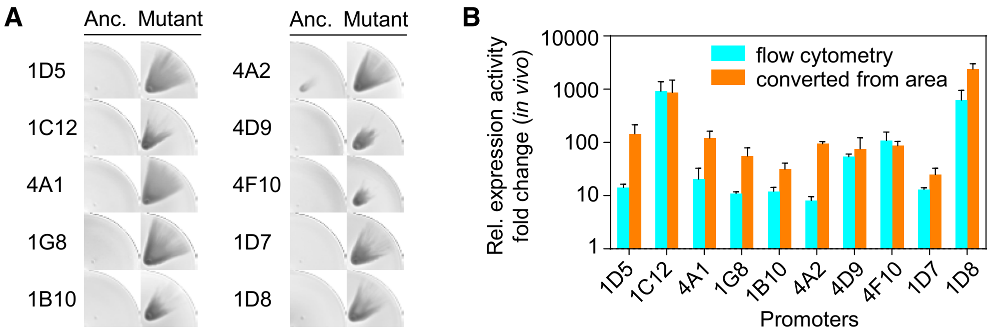

Figure EV4. Improved expression activity of RNAP mutants.

A Improvement in RNAP recognition of 10 selected synthetic promoters as shown in Fig 4B is demonstrated visually, by comparing the area size of fan shapes formed by phages carrying corresponding mutant RNAP genes with those carrying the wild-type T7 RNAP gene (Anc.). *Escherichia coli* FM15 cells carrying low-copy accessory plasmids were used. The images of a quarter of the agar plate containing a representative fan-shaped pattern are shown.

B Fold changes in the relative expression activity of RNAP mutants on their corresponding target promoters. The activity of the mutants was measured either by the *in vivo* transcriptional assay based on flow cytometry (Materials and Methods) or converted from the area of fan-shaped pattern using the transfer function in Fig EV2D. Data represent mean values ± s.d. for three biological replicates.

Source data are available online for this figure.

© 2022 The Authors
