# Supplementary material for: Exploiting spatial dimensions to enable parallelized continuous directed evolution
Source: Mol Syst Biol. 2022 Sep 21;18(9):e10934. doi: 10.15252/msb.202210934 (PMC9491160; doi:10.15252/msb.202210934)
Supplement: Supplementary file 9 — PDF+ [file MSB-18-e10934-s009.pdf]

Article

SOURCE DATA

OPEN ACCESS

molecular systems biology

Exploiting spatial dimensions to enable parallelized continuous directed evolution

Ting Wei[1,*,†], Wangsheng Lai[1,†], Qian Chen[1,2,†], Yi Zhang[1,†], Chenjian Sun[1,2], Xionglei He[3], Guoping Zhao[1,4], Xiongfei Fu[1,2,**] & Chenli Liu[1,2,***]

Abstract

Current strategies to improve the throughput of continuous directed evolution technologies often involve complex mechanical fluid-controlling system or robotic platforms, which limits their popularization and application in general laboratories. Inspired by our previous study on bacterial range expansion, in this study, we report a system termed SPACE for rapid and extensively parallelizable evolution of biomolecules by introducing spatial dimensions into the landmark phage-assisted continuous evolution system. Specifically, M13 phages and chemotactic *Escherichia coli* cells were closely inoculated onto a semisolid agar. The phages came into contact with the expanding front of the bacterial range, and then comigrated with the bacteria. This system leverages competition over space, wherein evolutionary progress is closely associated with the production of spatial patterns, allowing the emergence of improved or new protein functions. In a prototypical problem, SPACE remarkably simplified the process and evolved the promoter recognition of T7 RNA polymerase (RNAP) to a library of 96 random sequences in parallel. These results establish SPACE as a simple, easy to implement, and massively parallelizable platform for continuous directed evolution in general laboratories.

Keywords bacteriophage; directed evolution; range expansion; spatial competition; virus spreading

Subject Categories Methods & Resources; Microbiology, Virology & Host Pathogen Interaction

DOI 10.15252/msb.202210934 | Received 24 January 2022 | Revised 2 September 2022 | Accepted 5 September 2022

Mol Syst Biol. (2022) 18: e10934

Introduction

Directed evolution mimics natural evolution and typically proceeds with iterative rounds of genotype diversification and selection for desired phenotype activity (Bloom & Arnold, 2009). The steps required in the library construction and selection/screening cycles of conventional directed evolution methods are generally labor-intensive and time-consuming. In order to improve the efficiency and reduce manual labor, researchers are exploring to bring in sophisticated mechanical instruments such as microfluidic/millifluidic systems (Agresti *et al*, 2010; Fallah-Araghi *et al*, 2012; Wong *et al*, 2018) and automated robotic platforms (Pham *et al*, 2017; Piatkevich *et al*, 2018; Chory *et al*, 2021). Continuous directed evolution methods, on the other hand, employ delicate biological designs to enable autonomous cycles of mutant library construction and selection by coupling gene functions of interest to the fitness of replicating organisms (Esvelt *et al*, 2011; Crook *et al*, 2016; Ravikumar *et al*, 2018; English *et al*, 2019), leading to rapid optimization of biomolecules with little human intervention required. One representative of these methods, phage-assisted continuous evolution (PACE) has been applied to evolve a wide range of protein functions such as the specificity of RNA polymerase, TALEN, and Cas9, target specificity and drug resistance of proteases, activity and target compatibility of base editors, and improved soluble expression of proteins (Esvelt *et al*, 2011; Carlson *et al*, 2014; Dickinson *et al*, 2014; Hubbard *et al*, 2015; Badran *et al*, 2016; Bryson *et al*, 2017; Packer *et al*, 2017; Hu *et al*, 2018; Wang *et al*, 2018; Thuronyi *et al*, 2019; Richter *et al*, 2020; Blum *et al*, 2021). It links the desired property of biomolecules to phage propagation to enable rapid rounds of evolution, and utilizes a chemostat-like apparatus (upper panel, Fig 1A) to constantly supply both uninfected host bacterial cells and a continuously diluted environment for selection. Although the PACE system has been brilliantly designed, the complexity of continuous culturing apparatus and requisite process control make it challenging to perform continuous directed evolution in a highly parallelized form (d'Oelsnitz & Ellington, 2018) unless it is facilitated with robotic platforms (DeBenedictis *et al*, 2022). This limits its use for important application tasks such as evolution toward multiple targets and in different conditions, or high experimental replication to map evolutionary trajectories (Harms & Thornton, 2013) in general

1 CAS Key Laboratory for Quantitative Engineering Biology, Shenzhen Institute of Synthetic Biology, Shenzhen Institutes of Advanced Technology, Chinese Academy of Sciences, Shenzhen, China
2 University of Chinese Academy of Sciences, Beijing, China
3 State Key Laboratory of Biocontrol, School of Life Sciences, Sun Yat-Sen University, Guangzhou, China
4 CAS Key Laboratory for Synthetic Biology, Institute of Plant Physiology and Ecology, Shanghai Institutes for Biological Sciences, Chinese Academy of Sciences, Shanghai, China
*Corresponding author. Tel: +86 755 86585245; E-mail: ting.wei@siat.ac.cn
**Corresponding author. Tel: +86 755 86585243; E-mail: xiongfei.fu@siat.ac.cn
***Corresponding author. Tel: +86 755 86585245; E-mail: cl.liu@siat.ac.cn
[†]These authors contributed equally to this work

© 2022 The Authors. Published under the terms of the CC BY 4.0 license.

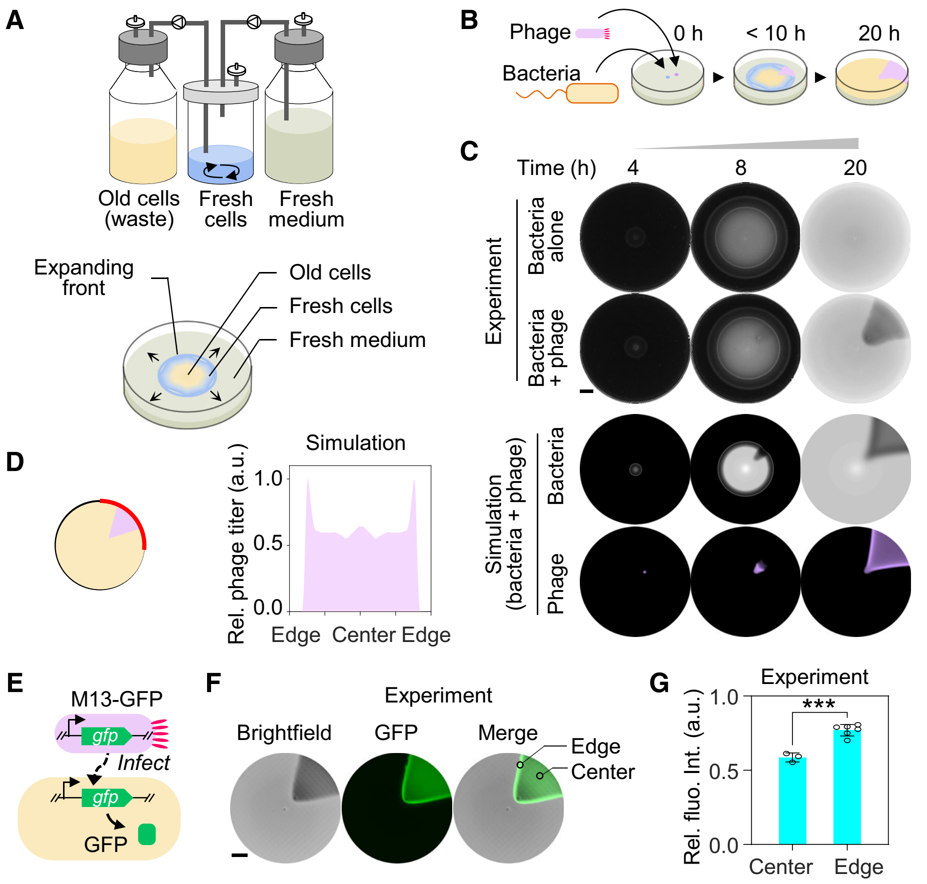

Figure 1. Formation of the fan-shaped pattern by phage infection during bacterial range expansion.

A Illustration of the analogy between bacterial range expansion in a semisolid agar plate and a chemostat-like continuous culturing device.

B Phage propagation during bacterial range expansion leads to the formation of a visible fan-shaped region of lower cell density.

C Time-lapse photographs of typical patterns obtained for bacteria alone and bacteria with phage, with corresponding model simulations. Scale bar represents 1 cm.

D The plot of the simulated phage-titer profile along bacterial expanding front at the time point of 24 h. The position of expanding front is shown by the red arc line in the schematic.

E Design of a reporter phage M13-GFP for fluorescence imaging of phage-infected region.

F Visualization of infected region. *Escherichia coli* FM15 was inoculated at the center of a semisolid agar plate and the reporter phage M13-GFP was inoculated 1-cm away from the center. Fluorescence images (Materials and Methods) were captured after overnight incubation using FITC channel and an exposure time of 200 ms. Scale bar represents 1 cm.

G Relative fluorescence intensity at the center and edge positions of the infection zone as shown in (F). The relative intensity was obtained by dividing the detected values with the maximum value of green fluorescence intensity on the plate. Data represent mean values ± s.d. for three values of the center and six values of the edge from three biological replicates. Two-tailed *t*-test was used to compare two groups. ****P* = 0.0005.

Source data are available online for this figure.

laboratories not equipped with sophisticated microfluidic or robotic instruments.

The once-humble agar plate is increasingly seen as a useful platform that can address questions not amenable to study by standard "well-mixed" liquid culture (Baym *et al*, 2016; Bosshard *et al*, 2017; Fraebel *et al*, 2017; Ni *et al*, 2017; Shih *et al*, 2018; Liu *et al*, 2019). For instance, it has recently been utilized to study the evolution of antibiotic resistance (Baym *et al*, 2016) and the colonization strategies of bacterial range expansion (Liu *et al*, 2019). In such studies, bacterial cells are typically inoculated at the center or edge of the semisolid agar plate. The subsequent range expansion of bacteria is led by a propagating front of growing cells (blue circle in lower panel of Fig 1A) moving outward toward the uncolonized territory, while cells with lower motility are left behind to grow until the nutrients are exhausted (Cremer *et al*, 2019). The steadily advancing front and associated growing wake thus provide a "moving chemostat" that harbors exponentially growing fresh cells (Koster *et al*, 2012; Cremer *et al*, 2019), with spontaneous separation from old cells whose growth and motility slow dramatically as nutrients are depleted (lower panel, Fig 1A).

In this study, we sought to develop a new method that combines the advantages of spatial range expansion and PACE. Compared with conventional methods where evolution plays out only on the temporal dimension, bringing in spatial dimensions enables visualization, separation of different evolutionary events, and straightforward operation without requirements for special culturing or monitoring equipment.

 © 2022 The Authors

Results

We began by inoculating M13 phages, which conduct chronic infections without lysing or severely damaging their host cells, (purple dot in Fig 1B) in front of a motile bacterial inoculum. A substantial fraction of the expanding cells in the front encountered phages and got infected, resulting in a slowdown of their subsequent growth (Appendix Fig S1). Progeny phages were then produced and carried forward along the expansion route by infecting neighboring fresh cells. This combination of cell migration and repeated phage infection cycles was expected to result in the formation of a visible fan-shaped infection zone with lower cell density than the uninfected regions (Li *et al*, 2020) (Fig 1B). Experiments showed that a suspension of exponentially growing *Escherichia coli* FM15 cells (Appendix Table S1), when inoculated at the center of an 8.5-cm Petri dish containing 10 ml of LB medium and 0.25% agar, formed a uniform bacterial lawn after overnight incubation (first row in Fig 1C). In contrast, inoculating bacteria at the center and 10^3 PFU of M13 phages 1 cm away from the center of an identical semisolid agar plate led to formation of a dark (low cell density) fan-shaped pattern in the midst of a white (high cell density) bacterial lawn after overnight incubation (second row in Fig 1C). The fan-shaped pattern of low cell density area was stable for days until the agar dried up.

To gain a quantitative understanding of the patterning process, we developed a mathematical model, RESIR (Range Expansion with Susceptible Infected Recovered kinetics) model derived from previous models (Kermack & McKendrick, 1927; Cremer *et al*, 2019), based on the characterized properties of the bacteria–phage interaction (Fig EV1A, Appendix Fig S2, Materials and Methods). In our model, numerical simulations with realistic parameter values (Appendix Table S2) recaptured the fan-shaped pattern as the experiments (bottom two rows in Fig 1C). The saturated cell density in this fan-shaped region is lower than that of uninfected region because the nutrient is partially consumed by phage production and is hence less available for supporting bacterial growth (Appendix Fig S3), thereby yielding a visible low cell density region. The development of the fan-shaped pattern was mainly driven by the expansion in the radial direction, supplemented by the extension in the lateral direction (Fig EV1B).

One prediction of the model was that, at the boundaries of the fan, uninfected cells migrating side by side with infected cells continuously served as fresh hosts for progeny phages during the range expansion, resulting in a moderately higher phage titer at the sideward edge than in the central region of the fan-shaped infection zone (Fig 1C and D). To verify this, we visualized the phage-infected bacteria by introducing a fluorescence gene accompanied with phage infection. Specifically, we constructed a reporter phage M13-GFP harboring a "superfolder" variant of green fluorescent protein (*gfp*) gene located downstream of gene *IV* in the wild-type M13 phage genome. The infection of M13-GFP phage introduces the *gfp* gene into the host bacteria. Thus, the bacteria infected by M13-GFP phage could be visualized by the fluorescent signal which reflects the expression level of phage genes locally (Fig 1E). As shown in Fig 1F and G, the brightest fluorescent signals in the semisolid agar plate, which suggested the highest cumulative expression of genes in the phage genome including *gfp*, overlapped with the sideward edge of the infection zone, consistent with the model simulation (Fig 1D).

The model simulation also predicted that the size of the fan-shaped pattern increased with the phage production rate (Fig EV1C). To test the proportionality predicted by the model, we borrowed one key design in PACE, an activity-dependent phage propagation module located in an accessory plasmid (Esvelt *et al*, 2011), which constructs a linkage between the function of the biomolecule to be evolved and phage propagation via the activation of *gIII* expression. It is known that the production of M13 phage scales with increasing levels of its minor coat protein pIII (encoded by *gIII*) over concentrations spanning two orders of magnitude (Rakonjac & Model, 1998). We used this design to vary the phage production rate by altering the expression level of *gIII*. Specifically, a selection phage (Esvelt *et al*, 2011) was constructed by replacing the intrinsic *gIII* of the M13 genome with the wild-type T7 RNA polymerase (RNAP) gene, while a copy of *gIII* was inserted into an accessory plasmid (Esvelt *et al*, 2011) in the host *E. coli* cell (Fig EV2A). The expression of *gIII* was put under the control of a library of 17 T7 promoter variants (Appendix Table S3), on which wild-type T7 RNAP exhibits different levels of activities ranging from 0.005 to 85% of the activity of wild-type T7 RNAP on the T7 promoter (Fig EV2B). When a high-copy-number accessory plasmid was used (Materials and Methods, Appendix Table S4), the area sizes of the fan-shaped pattern steadily increased with increasing expression levels of *gIII*, saturating at an activity level approximately 1.7% of the wild-type T7 RNAP activity on the T7 promoter (Fig EV2C). To distinguish more finely between high expression levels, a low-copy-number accessory plasmid was employed (Materials and Methods, Appendix Table S4). The combined utilization of the high- and low-copy-number accessory plasmids provided a measurement range of expression activity spanning 4 orders of magnitude (Fig EV2D). These results suggested that the area of the fan-shaped pattern could be used as a straightforward assessment of the activity of interest. We first used the high-copy-number accessory plasmid unless noted otherwise.

To develop our system of spatial directed evolution, we next sought to modulate the second key design in PACE, the mutagenesis plasmid (Badran & Liu, 2015; Bryson *et al*, 2017), to match the needs of the applications in semisolid media. The mutagenesis plasmid (MP4) typically used in PACE includes three mutators: DnaQ926, a dominant negative mutant of the delta domain of *E. coli* DNA polymerase, Dam, DNA adenine methyltransferase, and SeqA, a negative regulator of replication initiation (Badran & Liu, 2015). The expression of these mutator genes is driven by a small molecule inducer arabinose. Differently, for our spatial evolution system, we employed phage shock protein promoter (P_{psp}) (Brissette *et al*, 1990, 1991) to drive the expression of the mutators upon M13 phage infection via a pIV-dependent signaling cascade (Brissette *et al*, 1991), generating the mutagenesis plasmid MP-s for this study (Appendix Table S4). The stringency of the P_{psp} was confirmed by using *E. coli* FM15 cells carrying a plasmid with *gfp* under the control of P_{psp} (Fig 2A). The cells exhibited green fluorescence only in the presence of phage infection (Fig 2B), confirming the stringency of this promoter could prevent undesired induction of mutagenesis in bacteria cells before they came into contact with phages. The mutation rate conferred by MP-s was measured to be comparable to that of MP4, which is 4.4×10^{-7} and approximately 5.9×10^{-4} substitutions per bp per generation for *E. coli* and M13 phage, respectively (Badran & Liu, 2015; Materials and Methods).

© 2022 The Authors

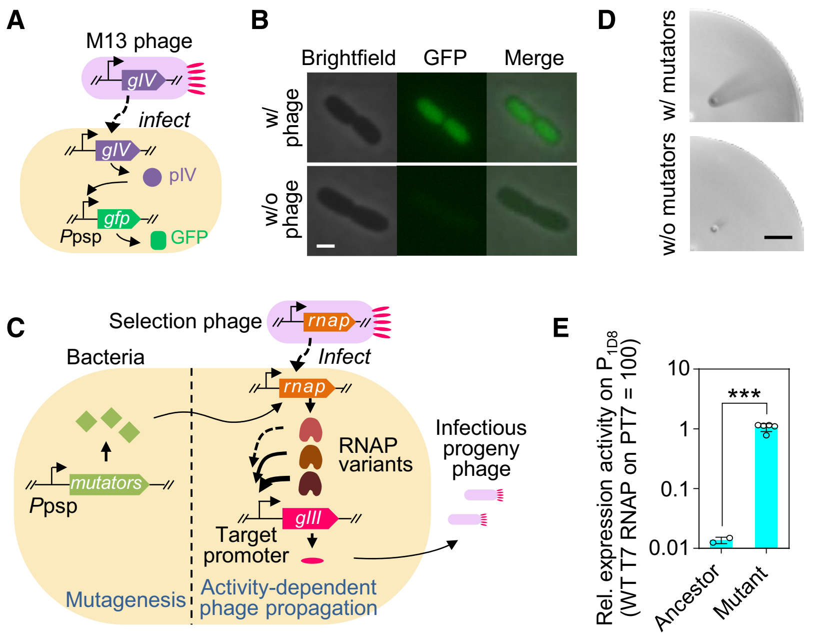

Figure 2. Establishment of a prototypic SPACE system.

A Reporter system to verify phage infection-inducible expression of proteins conferred by the phage shock protein (psp) promoter via a pIV-dependent signaling cascade (Brissette *et al*, 1991). In the mutagenesis plasmid MP-s developed in this study, *gfp* was replaced with mutator genes (Badran & Liu, 2015).

B Microscopic images of *E. coli* FM15 cells carrying a plasmid with *gfp* gene downstream of the psp promoter with or without wild-type M13 phage infection. Scale bar: 1 μm.

C Schematic design of SPACE coupling T7 RNAP activity with the expression of *gIII*. The host bacteria carry a mutagenesis module and an activity-dependent phage propagation module, which are harbored by the mutagenesis plasmid MP-s and the accessory plasmid, respectively.

D Photographs of a quarter of the semisolid agar plate of a SPACE experiment using high-copy accessory plasmid to improve within-cell activity of T7 RNAP on a synthetic promoter 1D8. An *E. coli* FM15 strain carrying no MP was used as control. Scale bar: 1 cm.

E Activity in cells of T7 RNAP and its mutants from the SPACE experiment in (D). Two clones of the ancestor and five clones of the mutants obtained from the evolution towards 1D8 recognition were measured by *in vivo* transcriptional assay (Materials and Methods). Two-tailed *t*-test was used to compare the two groups. ***$P = 0.0001$. Data represent mean values ± s.d.

Source data are available online for this figure.

By introduction of the accessory plasmid carrying the activity-dependent phage propagation module and MP-s carrying the *in vivo* mutagenesis module into our motile host strain, *E. coli* FM15, we established a prototypic system named as SPACE, standing for Spatial PACE. In SPACE, ancestor selection phages (Esvelt *et al*, 2011) carrying a wild-type T7 RNAP gene infect bacterial cells, and the expression of mutator genes induced by the infection leads to mutations in the RNAP gene during the replication of phage genome, and then expression of different RNAP variants. Desired RNAP variants with improved activity on the target synthetic promoter activate the expression of *gIII* on an accessory plasmid to produce infectious progeny phages, which in turn infect neighboring susceptible bacterial cells and repeat the process (Fig 2C). In contrast, RNAP variants that do not lead to sufficient production of pIII and infectious progeny result in the formation of typically much smaller fan-shaped pattern or no infection zone at all.

As an initial test of SPACE, T7 RNAP was evolved to recognize a synthetic promoter named 1D8 (Appendix Table S3) with nine bases different from wild-type T7 promoter. A distinct fan-shaped pattern was observed after 20 h development (upper panel of Fig 2D), while no infection zone was formed on the host cells lacking the mutagenesis module (lower panel in Fig 2D). From the sideward edge of the fan-shaped pattern, five phage clones were purified. Their average transcriptional activity on the promoter 1D8 was 70-fold greater than that of the wild-type T7 RNAP (Fig 2E, Materials and Methods). These results established the ability of the SPACE system to evolve enzyme activities on a single agar plate with minimal efforts.

To better understand the underlying evolutionary process of the SPACE experiment, we extended the RESIR model to describe competitions between two phages with different progeny production rates. The simulated competition results in a spatial separation between the two phages, of which the strong phages with higher progeny production rate dominate outer area near the sideward edge of the infection zone, while the weak phages with lower progeny production rate are confined inside (Fig EV3A and B). To experimentally validate the spatial separation of phages, we competed a phage M13s carrying a red fluorescent protein (*rfp*) gene in wild-type M13 genome against a weaker variant (M13w) carrying a *gfp* gene (Fig 3A). M13w harbored three mutations K184A/R186A/D187A in its *gIII*, which resulted in lower progeny productivity than wild-type M13 (Deng & Perham, 2002). The expression of red or green fluorescent proteins induced by the infection of M13s or M13w could thereby indicate the spatial distribution of these two

 © 2022 The Authors

phages (Fig 3B). Their spatial abundance patterns were characterized after 20 h of competition assay (Materials and Methods) with an initial titer ratio of 1:1 between M13w and M13s. As expected, M13s (red) dominated the outer area of the fan-shaped infection region along the direction of the sideward edges, while the M13w (green) was localized in the inner area mostly around the initial inoculation spot (Fig EV3C and D).

To more closely reflect actual evolutionary process, in which stronger phages may be generated at very low frequencies, we repeated the competition assay with an M13w: M13s inoculant ratio of 10^5:1. Both experimental and theoretical results showed that the strong phage M13s could eventually outcompete the weak and take over the population as the phage population spread via its range-expanding host (Fig 3C and D, Appendix Fig S4). And the strong phage is accumulated most efficiently at the sideward edge of the infection zone due to the most active infections occurring there. Thus, during SPACE process, the advantageous phage mutants with high production rates, which usually occur at very low frequencies in an evolutionary process, would be autonomously separated from the weaker and get enriched rapidly at the sideward edge of the infection zone, as illustrated in Fig 3E.

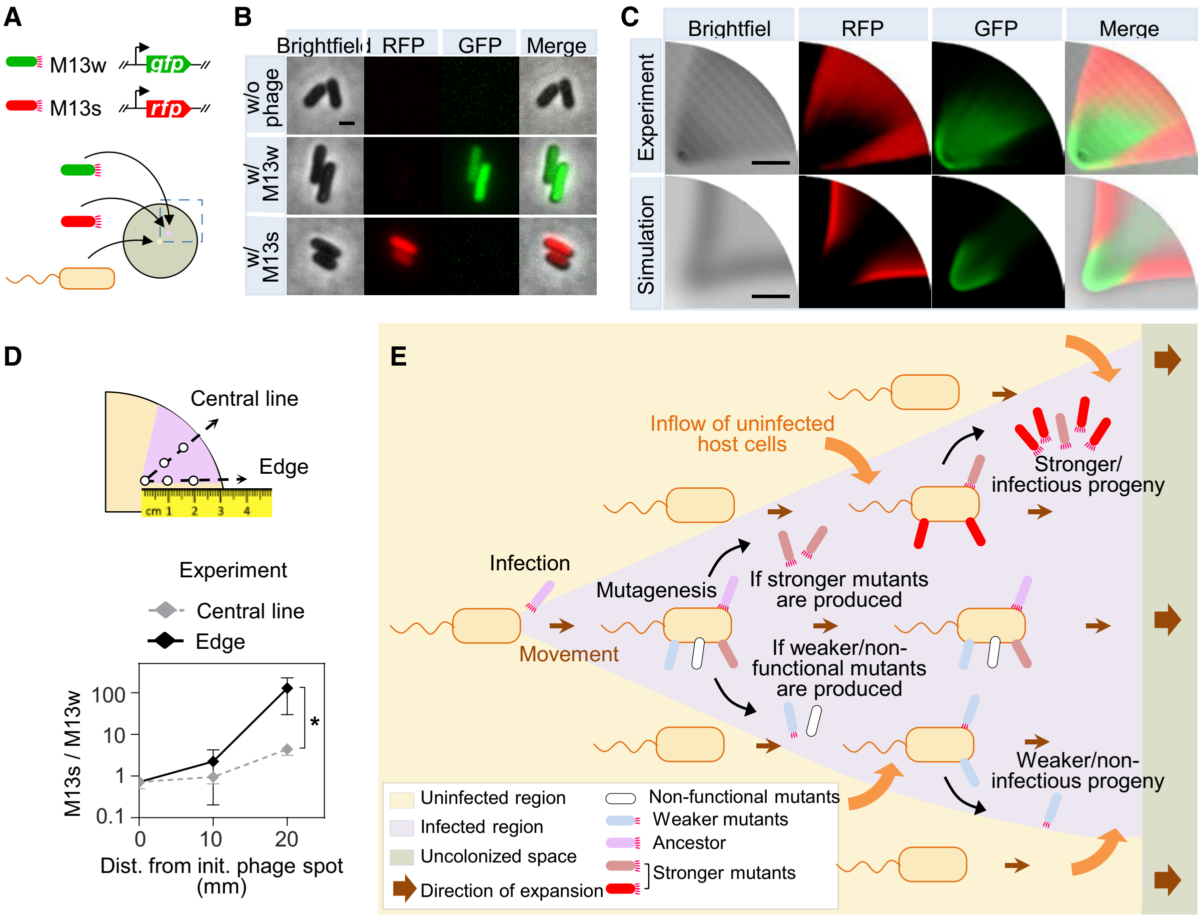

Figure 3. Spatial competition and adaptation of phages during range expansion of their hosts.

A Design of a competition assay between phages with different production rates. M13s carries a red fluorescent protein (*rfp*) gene in a wild-type M13 genome, while a weaker phage variant M13w carries a green fluorescent protein (*gfp*) gene. The phage titers of M13s and M13w were 9.6 × 10^8 and 1.3 × 10^7 PFU/ml, respectively, after 2.5 h propagation in *E. coli* FM15 by shaking incubation at 37°C with a multiplicity of infection (MOI) of 0.001.

B Microscopic images of *E. coli* FM15 cells with or without phage infection. Scale bar represents 1 μm.

C The experimental result shows raw photographs of a representative two-phage competition 20 h after initial inoculation with 10^5:1 mixture of M13w: M13s at 1 cm away from the center. The simulation result is the outcome of competition between two phages PW and PS with relative production rates of 40 and 100, respectively. Scale bar represents 1 cm.

D Phage titer ratio of M13s to M13w along the central line and the edge of the fan-shaped infection zone in the experimental result in (C). Samples were collected from different positions as shown in the schematic and phage titer was quantified by qPCR (Materials and Methods). Data represent mean values ± s.d. for three independent assays. Two-tailed *t*-test was used to compare two groups. *P = 0.028.

E Illustration of the phage spatial evolution process during the range expansion of the host bacteria. Mutants with improved activity to produce infectious progeny phages continuously infect neighboring susceptible bacterial cells and get enriched along the sideward edge of the fan-shaped infected zone; meanwhile, weak mutants that do not lead to sufficient production of infectious progeny result in typically much smaller fan-shaped pattern or no visible infection region at all.

Source data are available online for this figure.

© 2022 The Authors

We next demonstrated that SPACE is scalable and parallelizable. Ninety-six SPACE experiments were carried out concurrently to evolve T7 RNAP to recognize a library of random T7 promoter variant sequences (Fig 4A). The starting selection phage carrying wild-type T7 RNAP was inoculated onto 96 semisolid agar plates, each of which contained an inoculum of host cells harboring an accessory plasmid containing a random T7 promoter variant (Appendix Table S3) and the mutagenesis plasmid MP-s. Twenty out of the 96 experiments were deemed as successful evolution based on the formation of the fan-shaped pattern, and the evolved phages were isolated and sequenced for mutations in the T7 RNAP mutants (Fig 4B). As the size of the fan-shaped pattern is linked to the activity of RNAP (Fig EV2), we compared the size of the infected region to assess the improvements in enzyme activity, and confirmed that the isolated mutant phages presented larger fan-shaped patterns than the ancestor (Figs 4B and EV4A). The expression activity levels of the evolved RNAP mutants on their target sequences were further measured *in vivo* based on flow cytometry (Fig EV4B, Appendix Fig S5), and also calculated using the area-to-activity transfer function (given in Fig EV2D). Roughly consistent with the calculation, the fold change in measured activities of the RNAP mutants ranged from 8.1 (4A2) to 918.3 (1C12) as compared with the activity of wild-type T7 RNAP on the corresponding promoters (Fig EV4B). Several evolved T7 RNAP mutants were further purified and assayed *in vitro* (Materials and Methods), and the purified RNAP mutants exhibited improvements ranging from 3.4-fold (1G8) to 72.9-fold (1D8), compared with the starting enzyme (Fig 4C). Aside from mutations at frequently reported sites including E222, N748, Q758, D770, and E772 (Ikeda *et al*, 1993; Raskin *et al*, 1993; Rong *et al*

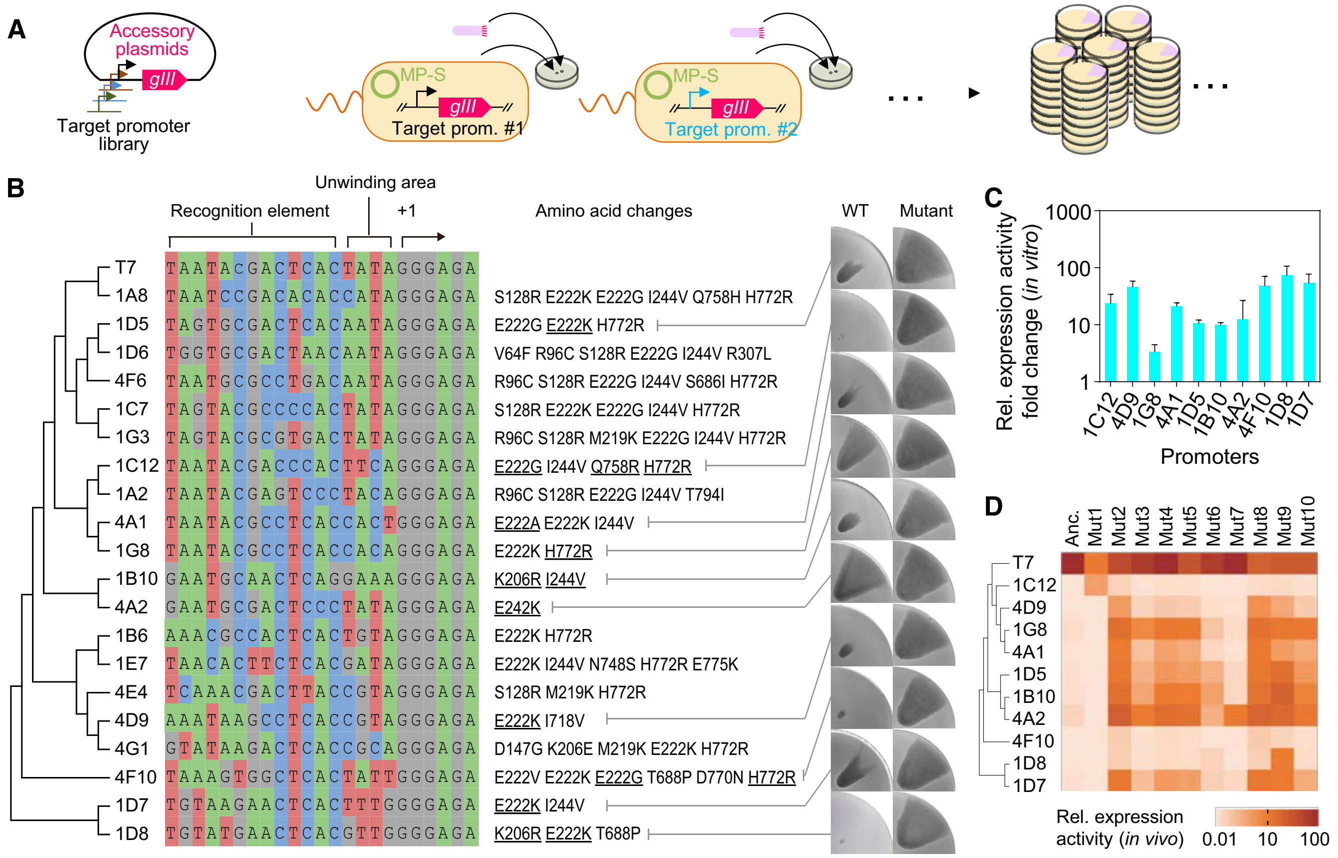

Figure 4. Parallelizable SPACE system.

A An Illustration of the parallelized SPACE experiments. Each FM15 strain carrying an accessory plasmid with a specific target promoter on it was inoculated on an agar plate to launch the experiments in parallel.

B List of library promoter sequences for which SPACE produced improved RNAP variants relative to wild-type RNAP (wild-type T7 promoter sequence is at the top). For 10 selected promoters, improvement of RNAP performance is demonstrated visually on the right, by comparing the size of fan shapes formed by phages carrying corresponding mutant RNAP genes (underlined mutations) with those formed by ancestor (anc.) phages carrying the wild-type T7 RNAP gene. The images of a quarter of the agar plate containing a representative fan-shaped pattern are shown.

C Fold change in the relative expression activity of the evolved RNAP mutants as compared with the wild-type T7 RNAP, for the 10 selected promoter sequences corresponding to the images in (B). Results were obtained by *in vitro* transcriptional assay (Materials and Methods). Data represent mean values ± s.d. for three biologically independent assays.

D Expression activity of 10 RNAP mutants on T7 promoter and 10 promoter variants measured by *in vivo* transcriptional assay. Mutants are arranged in the same order as their original target promoters listed on the left side of the heatmap. Amino acid changes of each mutant are listed in Appendix Table S5. Data are normalized so that the activity of wild-type RNAP on the T7 promoter is 100; the mean for three biologically independent replicates is shown (Materials and Methods).

Source data are available online for this figure.

 © 2022 The Authors

al, 1998; Imburgio et al, 2000; Meyer et al, 2015), some new mutations such as I244V, K206E/R, M219K, and T688P were detected repeatedly. They might play important roles in the recognition of the corresponding artificial promoters, which needs further investigations.

To assess the orthogonality and promoter malleability of the evolved RNAP mutants, we generated an activity map based on the *in vivo* measurements of 11 promoters against 10 RNAP mutants (Fig 4D, Appendix Fig S6, Appendix Tables S3 and S5). All evolved RNAP mutants retained their activities on the T7 promoter. Besides wild-type T7 RNAP, Mut1 and Mut7 were orthogonal, recognizing only their target sequences (1C12 and 4A2, respectively) and the T7 promoter. Apart from these, off-target crosstalk is consistently observed between different pairs (Fig 4D), which needs further efforts to remove the promiscuity of these RNAPs if they are to be applied in genetic circuit design (Tabor, 2012). The mutation E222K, known to be a specificity broadener (Ikeda et al, 1993), led to nonspecific activity on almost all sequences tested. Aside from changing from glutamic acid to lysine, other mutations at the same site including E222A (mut5) and E222G (mut6) produced mutants with similar off-target activities. H772R (mut8) also showed some non-specific activity. However, combining E222G and H772R, with one more mutation Q758R, resulted in mut1, which, as described, is highly orthogonal. This implied an underlying epistatic phenomenon. Construction of an exhaustive activity map of RNAP mutants and random sequences would be of interest to deepen understanding of the origin of the orthogonality and promoter malleability. To this end, SPACE can be used to generate abundant mutants and assay the activity without additional infrastructure.

Discussion

Range expansion is a widely observed phenomenon in nature, which involves the movement and successful establishment of natural populations across new territories due to biological invasion, anthropogenetic habitat conversion, or changes of the abiotic and biotic environmental factors (Andow et al, 1990; Walther et al, 2002; Parmesan & Yohe, 2003; Hastings et al, 2004; Excoffier et al, 2009; Fronhofer & Altermatt, 2015; Gandhi et al, 2016; Ochocki & Miller, 2017; Ramirez et al, 2019; Aguirre-Liguori et al, 2021; Liu et al, 2021). Although nonmotile, nonlethal viruses can be easily transmitted via their host species conducting range expansion and therefore spread to broader territories (Jones, 2009; Kareinen et al, 2020). It has been assumed that movements of migrating animal species could enhance the spread of pathogens including zoonotic viruses posing severe threats to human health (Altizer et al, 2011). However, the mechanism by which the range expansion of host species influences the adaptation and evolution of their viruses remains to be elucidated.

It is recognized that range-expanding species experience active evolutionary changes at the expanding front (Deforet et al, 2019; Miller et al, 2020). The fast-dispersing individuals with superior moving ability outrun other individuals of the species and inevitably accumulate at the front, resulting in an evolutionary increase in the moving ability in successive generations. This process has been proposed as a new evolutionary mechanism called "spatial sorting" (Shine et al, 2011; Phillips & Perkins, 2019). Researchers also found that parasite that infects range-expanding hosts will itself be subjected to spatial sorting (Shine et al, 2011), and its improved ability to successfully infect hosts is favored during this evolutionary process (Kelehear et al, 2012). Considering that viruses are obligate parasites and copropagate with their hosts, one could intuitively speculate that viruses that infect a range-expanding host will experience the same evolutionary process with the host, that is, the evolutionary changes of viruses mostly accumulating at the front of host expansion. However, our results in this study have proven otherwise. Unlike the spatial sorting of host species (Deforet et al, 2019; Miller et al, 2020), the active infection of neighboring susceptible host cells leads to the spatial sorting of phages with different progeny productivities perpendicular to the direction of host front propagation. Therefore, other than at the expanding front, bacteriophagic genotypes associated with higher phage progeny production rate are significantly accumulated along the sideward edge of the fan-shaped infection zone. It can also serve as a null hypothesis for the prediction of viral spread and evolution during host range expansion for future ecological studies of higher organisms.

Moreover, we applied this ecological insight to develop a SPACE system. Unlike the 17 continuous directed evolution tools comprehensively summarized by a recent review (Morrison et al, 2020), our SPACE system introduces spatial dimensions into continuous directed evolution to streamline protein engineering. This remarkably reduces the complexity of the continuous directed evolution apparatus, and enables direct visual assessment of the progress of the evolution experiments through the link between phage production rate and the size of the infection zone. As to PACE system, its selection stringency has to be optimized from time to time to avoid complete phage washout. To accelerate the selection process, the flow control needs to be closely monitored, either by manually collecting samples from the lagoon and checking the intensity of reporter signals as well as the phage titer, or by using a specialized in-line monitoring instrument (Carlson et al, 2014; Badran et al, 2016), which limits the number of evolution experiments that can be performed in parallel. Moreover, PACE relies solely on the time scale to select for stronger phage mutants based on their faster reproduction in the well-mixed liquid system. In SPACE system, the stronger mutants are spontaneously separated from weaker ones through spatial competition and get most enriched at the rim end of the sideward edge of the fan-shaped infection region. In addition, our model also predicted that the fold-enrichment rate of beneficial mutants provided by the SPACE system is higher than the optimal rate that a chemostat-like liquid continuous culturing system could achieve, regardless of the detailed progeny production rates of the mutants (Appendix Fig S7). These features of SPACE provide convenience to enable evolutionary applications that require extensive parallelization with efficiency, and it is easy to implement in general biology laboratories without expensive automated robotic experimental systems (DeBenedictis et al, 2022).

We have demonstrated that SPACE could improve the *in vivo* transcriptional activity of T7 RNAP on an artificial promoter by 70-fold after a round of overnight experiment (~20 h), which is comparable to the ~100-fold increase in activity on P_{T3} achieved by PACE at 28 h (Carlson et al, 2014). However, current format of SPACE system still has its drawbacks. For example, the selection stringency cannot be flexibly adjusted within one round of experiment, and the phage generations need to be increased by transferring evolved

© 2022 The Authors

phages to a fresh agar plate to start a new run (Appendix Text S1). As a future upgrade, additional relevant factors such as the concentrations of agar and chemoattractant in the media could be tuned to change the expansion speed of bacteria (Liu *et al*, 2019) and hence enable more flexible control over the selection stringency of the system. We expect that the increased expansion speed would result in higher selection stringency, meaning that mutants have to exhibit higher level of improvements in their activity to co-propagate with the range-expanding bacteria and outcompete other mutants (Appendix Fig S8). In addition, an attractive application of SPACE is to chart empirical fitness landscape by extensively parallelized evolution experiments. As the "fossils" will be left behind the expanding front in space, together with the convenient visual readout of competition outcomes, SPACE enables extensive exploration of fitness landscape. Overall, SPACE provides a powerful platform for applied research and fundamental investigation of evolutionary mechanisms.

Materials and Methods

Strains and media

DNA cloning was performed with chemically competent *E. coli* DH5α cells (TransGen, Beijing). All plaque assays, bacterial migration tests, *in vivo* transcriptional assays, and SPACE experiments were performed using *E. coli* FM15 strain. This strain was derived from *E. coli* MG1655-mCherry (Liu *et al*, 2019) by the following steps: (i) rendered F^+ by conjugation with *E. coli* K12 ER2738 (NEB); (ii) deletion of the α-fragment of LacZ gene with the λ Red recombineering system. The genotype of the resulting strain FM15 is *F′ proA + B+ lacI^q Δ(lacZ)M15 zzf::Tn10 (TetR)/λ– ilvG– rfb-50 rph-1 attB::Kan^R Δ(lacZ)M15*. Information about bacterial strains used in the study is listed in Appendix Table S1. Cells were cultured in Luria-Bertani medium (LB: 10 g/l NaCl, 10 g/l tryptone, 5 g/l yeast extract). LB containing 0.25% (w/v) agar (Huankai, Guangdong) was used for SPACE experiments. Antibiotics including chloramphenicol (25 μg/ml), tetracycline (15 μg/ml), carbenicillin (50 μg/ml), and spectinomycin (100 μg/ml) were added where appropriate. For *in vivo* transcriptional activity measurement, M9 medium (6.78 g/l Na_2HPO_4, 3 g/l KH_2PO_4, 1 g/l NH_4Cl, 0.5 g/l NaCl) was used, with casamino acid (CAA) and glucose supplemented where necessary.

Cloning and plasmid construction

The vectors were constructed with Gibson assembly (NEB) or ClonExpress II One Step Cloning (Vazyme, Nanjing) kits. PCRs were carried out with PrimeSTAR Max (Takara) or Q5 (NEB) following the manufacturers' instructions. Accessory plasmids (Esvelt *et al*, 2011) for T7 RNAP evolution contained, in order, an *rrnB* terminator, the promoter of interest, a strong RBS 5′-AAGGAGGAAAAAAAATATATA<u>ATG</u>-3′ where underlined bases represent the start codon, *gIII*, either the combination of *aadA* gene conferring spectinomycin resistance and pUC origin for a high-copy version or bla gene conferring carbenicillin resistance and SC101 origin for a low-copy version. Reporter plasmids were identical to SC101 accessory plasmids except for the replacement of *gIII* by *gfp*. T7 RNAP selection phage (Esvelt *et al*, 2011; Wu *et al*, 2021) was constructed by replacing all but the last 202 bp of *gIII* with the gene encoding T7 RNAP in M13K07 (NEB) helper phage and then removing the p15a origin of replication and *aph* gene to restore the M13 origin of replication. The mutagenesis plasmid (Esvelt *et al*, 2011; Badran & Liu, 2015) contained *dnaQ926*, *dam*, and *seqA* under control of psp operon. Information about plasmids used in the study is listed in Appendix Table S4.

Construction of promoter libraries

A target promoter library was constructed by altering bases of the original T7 promoter at positions from −17 to −1 relative to the transcriptional start site. In principle, this library should contain (4^{17}–1) variants, which is not a reasonable size to subject to biological process. Therefore, several rules were applied to narrow down the sequences to those which are more likely to act as promoters: (i) G + C content within 40–60%; (ii) no complicated secondary structure nor self-complementary structure; (iii) no consecutive GC regions; (iv) classifiable according to sequence similarity to T7 promoter. After this step, the size of the promoter library was reduced to approximately 2000. Ninety-six promoters were randomly selected from this library for the SPACE experiments. The sequences of these promoters are listed Appendix Table S3. These promoters were inserted upstream of *gIII* in the accessory plasmids.

Measurement of bacterial growth

The cells were grown according to the following protocol before assaying their growth curve. First, three single colonies of FM15 or FM15 carrying low-copy accessory plasmid with T7 promoter were picked from freshly streaked LB agar plates and grown overnight in 2 ml LB broth with appropriate antibiotics (tetracycline 15 μg/ml, carbenicillin 50 μg/ml) in Falcon tubes at 37°C with shaking (200 rpm). The overnight culture was then 1,000-fold diluted into prewarmed LB broth in Falcon tubes. After around 3-h shaking incubation when its optical density at 600 nm (OD_{600}) reached 0.1–0.2, the diluted culture was again 20-fold diluted into prewarmed LB media in 100-ml flask and incubated for another 2 h. Then the OD_{600} was measured with a Thermo Genesys 10S ultraviolet spectrophotometer and a proper volume of the culture was inoculated into 100 ml prewarmed LB to make an initial OD_{600} of around 0.01 in 500-ml flasks. The flasks were then incubated in a Warm-bath Shaker (ZHICHU) maintained at 37°C and 200 rpm. When the OD_{600} reached 0.1, bacterial suspensions were diluted to make OD_{600} 0.01 and different quantities of M13 or SP-T7 phages were added into each flask to obtain final titers of 10^7 or 10^8 PFU/ml. The shaking incubation continued and samples were taken for measurement of phage titer and cell number every 10 min. The phage titers were calculated by double-layer plating plaque assay or by qPCR in a CFX Connect Real-Time System (BIO-RAD) using TB Green *Premix Ex Taq*™ II (Tli RNaseH Plus) (Takara) and primer sets: fg2 5′-GCT ACAGCACCAGATTCAGC-3′ and rg2 5′-AAGCAAACTCCAACAGG TCA-3′ for all M13 phages; or SG2f 5′-CAATCGGTGATGGTCCTGT-3′ and SG2r 5′-AACTCCAGCAGAACCATATGATC-3′ for red fluorescence marker M13 phage. The cell numbers were assayed using a CytoFLEX flow cytometer (Beckman), and the gating strategy for this analysis was illustrated in Appendix Fig S5.

 © 2022 The Authors

Competition assay

GFP or RFP gene following a constitutive promoter J23100 was inserted into the phage genome downstream of *gIV*. Phages with different fluorescence genes were paired and mixed at titers ranging from 10^3 to 10^9 PFU/ml. Phage mixtures were made with different ratios dependent on experimental designs. FM15 cell suspensions at exponential phase ($OD_{600} \approx 0.2$) and 2-µl aliquots of the phage mixtures were inoculated onto soft agar. After 18–20 h incubation at 37°C, soft agar plates were inspected by fluorescence microscopy. When necessary, 2 µl of the soft agar containing bacteria and phages was aspirated with a pipettor from different positions at the center or the edge of the fan-shape infection zone, added to 998 µl fresh LB broth, and mixed by vortex at low speed. This liquid sample was then filtered through a 0.22-µm pore size PES syringe filter to remove bacteria, and subjected to quantification of phage particles by qPCR.

Single-cell fluorescence imaging

Fluorescence signals induced by M13s or M13w were measured after overnight co-culture of FM15 cells and phages in LB. For stringency test of *psp* promoter, FM15 cells carrying plasmid with/without *gfp* downstream of P_{psp} were cultured until mid-log phase and subjected to M13 phage infection at a multiplicity of infection (MOI) of approximately 10 for 2 h. Cells were imaged using a Nikon Ti-E microscope equipped with a Plan Apo λ 100× Oil Ph3 DM objective (N.A. = 1.4) and an Andor Zyla 4.2 sCMOS camera. A 1% agarose pad with 0.9% NaCl was used to immobilize the cells. After cell immobilization, images were acquired within 5 min at room temperature (RT). Fluorescent images were taken with an EGFP filter (49002; ET470/40×, ET525/50 m) or an RsRed filter (49005; ET545/30×, ET620/60 m), and a 40-ms exposure time.

Plate image capturing and processing

Plates were imaged using a Canon EOS 600D digital camera with a Canon EFS 18–135 mm lens and an exposure setting of f11, 1/500 s, ISO3200. The agar plates were illuminated by a white LED ring light with the diameter of 36 and 16 cm bellow (Liu *et al*, 2019). The area of fan shape was measured with ImageJ software (ver. 1.52a). For each plate, the region within a circle concentric to the plate bottom with radius 3.5 cm was used for area measurement.

Fluorescent images of agar plates with fan shapes were imaged using a Nikon Ti-E microscope and a Plan Fluor 4× PhL DL objective (N.A. = 0.1). Each picture of a complete plate was obtained by stitching 27 × 27 evenly divided square fields of view taken with the "ND processing" function of the NIS-Elements AR software (ver. 4.50.00). The green fluorescence images were taken with an EGFP filter (49002; ET470/40×, ET525/50 m), and the red fluorescence with an RsRed filter (49005; ET545/30×, ET620/60 m).

Mutation rate measurement

M13mp18 (Messing, 1983) (NEB), a phage vector containing beta-galactosidase (LacZ) alpha gene (507 bp) downstream of *gIV* in the M13 genome, was used for the modified *lacZ* inactivation assay. Overnight cultures of *E. coli* FM15 without mutagenesis plasmid, with P_{psp}-driven mutagenesis plasmid MP-s constructed in this study, and with MP4 (Badran & Liu, 2015) used in PACE were inoculated into 2 ml LB media supplemented with antibiotics where appropriate, and cultured at 37°C until log phase ($OD_{600} \approx 0.2$). Then approximately 20 PFU of M13mp18 was added to each bacterial culture. For induction of MP4, 25 mM (final concentration) of arabinose was added. The culture was continued to allow phages to propagate for 7–8 h. Supernatants containing progeny phages were collected after centrifugation and filtered through a 0.22 µm pore-size PES syringe filter. These samples were serially diluted, mixed with 200 µl log-phase culture of FM15 without mutagenesis plasmid, and applied to double-layered method with top agar containing 0.04% Bluo-Gal (Sangon, Shanghai) and 15 µg/ml tetracycline. The number of white or light blue plaques (lacZα$^-$ phenotype) and the total plaque number were counted and used as a measure of mutation frequency. After 24 h propagation on *E. coli* FM15 cells without mutagenesis plasmids, the testing phage vector did not produce any *lacZα*-inactive mutant plaques, while 3.9 and 3.1% of mutant plaques were produced by phages propagated on FM15 carrying MP-s and MP4 (Badran & Liu, 2015), respectively.

Range expansion-based continuous evolution

Soft LB agar was freshly prepared in 8.5-cm Petri dishes before each SPACE experiment. FM15 cells carrying both accessory plasmid and mutagenesis plasmid were cultured until its $OD_{600} \approx 0.2$, and 2-µl aliquots of the cell suspension were inoculated at the center of the agar plates. Two microliters of selection phages with a titer of approximately 5×10^8 PFU/ml were inoculated 1 cm away from the center of the soft agar. The inoculated plates were incubated at 37°C for 18–20 h, which was typically the duration of bacterial growth and phage propagation required for the formation of clear fan-shaped infection zone. After the incubation, 5 µl of the soft agar containing bacteria and phages was aspirated with a pipettor from the end of each edge of the fan shape, added to 495 µl fresh LB broth, and mixed by vortex at low speed. This liquid sample was then filtered through a 0.22-µm pore size PES syringe filter to remove bacteria, and stored at −20°C before use.

In vivo transcriptional activity measurement

FM15 cells were grown according to the following protocol before assaying their fluorescence. First, cells were inoculated from three single colonies on LB agar plates and grown overnight in 2 ml LB or M9 supplemented with 1% (w/v) CAA and 0.4% (w/v) glucose with 15 µg/ml tetracycline and 50 µg/ml carbenicillin in Falcon tubes at 37°C with shaking (200 rpm). Then, the overnight cultures of *E. coli* FM15 carrying low-copy reporter plasmids containing *gIII* downstream of T7 promoter or T7 promoter variants were diluted 1,000-fold in prewarmed M9 supplemented with 1% CAA and 0.4% glucose in Falcon tubes. After approximately 3 h, once the diluted cultures reached an OD_{600} of 0.1–0.2, the cultures were diluted 20-fold with prewarmed media in Falcon tubes again and incubated for another 2 h. OD_{600} was measured with Thermo Genesys 10S ultraviolet spectrophotometer and cultures were diluted with prewarmed media to OD_{600} of 0.02 then divided into a 96-well plate. Selection phages carrying wild-type or mutant RNAP genes were prepared by adjusting phage titers to approximately 10^{10} PFU/ml, and 20-µl

aliquots were also added to make up a total volume of 200 μl per well. The plate was then incubated at 37°C in a Digital Thermostatic Shaker (AOSHENG) maintained at 37°C and 1,000 rpm. Finally, after the 2-h incubation, a 10–20 μl sample of each culture was transferred to a new plate containing 180–190 μl PBS buffer and 2 mg/ml kanamycin to stop protein expression. For fluorescent protein maturation, all the samples were then incubated at 37°C in a Digital Thermostatic Shaker (AOSHENG) maintained at 37°C and 1,000 rpm for another 30 min. The fluorescence distribution of particles in each sample was assayed using a CytoFLEX flow cytometer (Beckman) with appropriate voltage settings (SSC: 500, FSC: 500, FITC: 2000); each distribution contained no < 50,000 events and was gated by the forward and side scattering using CytExpert (v2.2). The gating strategy for this analysis was illustrated in Appendix Fig S5. The intensity of fluorescence of each sample was calculated and normalized by the value of the wild-type T7 RNAP and T7 promoter pair.

T7 RNAP purification

T7 RNAP and mutants were cloned into isopropylthio-β-galactoside (IPTG) inducible expression vector, pQE82L, and transformed into *E. coli* BL21 competent cells. Clones were cultured in 5 ml LB with carbenicillin (50 μg/ml) overnight and transferred into 200 ml fresh LB with carbenicillin and grown until $OD_{600} \approx 1.2$. Cells were then induced by IPTG (final conc. 0.5 mM) and incubated for another 3 h in a shaking incubator at 30°C. For cell lysis, the bacterial suspension was divided into 50-ml aliquots and centrifuged at the maximum speed at 4°C for 15 min. With supernatants removed carefully, pellets were resuspended in equal volume of lysis buffer (50 mM NaH_2PO_4 pH 8.0, 300 mM NaCl, 0.5 mg/ml lysozyme, 0.5 mM dithiothreitol, DTT), and stored in freezer at −80°C immediately. After the samples were completely frozen (approximately 30 min), they were thawed on ice for 1 h, and this freeze–thaw cycle was repeated twice. The supernatants of bacterial lysates were collected by centrifugation and sterilized with 0.45 μm pore-sized filters. Ni-sepharose columns were balanced with 10-fold volume of elution buffer (50 mM NaH_2PO_4, 300 mM NaCl, pH 8.0). Then the lysate supernatants were loaded onto each column and kept steady until all liquids passed through the column. Columns were washed with 5 ml of elution buffer containing 20 μM imidazole, and then eluted with 5 ml elution buffer containing 20 and 50 μM imidazole. Elutes were collected with 1.5-ml microtubes. Proteins were dialyzed in 100 mM NaCl, 50 mM Tris–HCl, 0.1 mM DTT, 0.1 mM EDTA, 50% v/v glycerol, pH 8.0, and stored at −20°C.

In vitro transcriptional assay

The concentrations of purified T7 RNAP and mutants were determined by Bradford assay and then by Coomassie stain on a 10% SDS-PAGE gel. A 1.8-kb DNA fragment containing each promoter variant was amplified by PCR from the corresponding accessory plasmid. PCR products were purified with a QIAquick PCR purification kit, digested with *Dpn*I to remove the template plasmids, and purified again. The purified amplicons were used as templates of the *in vitro* transcriptional assay. Transcription reactions with volume of 10 μl consisted of 40 mM Tris–HCl (pH 7.9), 6 mM $MgCl_2$, 2 mM spermidine, 10 mM of DTT, 200 μM of ribonucleotide triphosphates, 0.3 μl of RNase inhibitor, 0.2 μl of pyrophosphatase, 1 μM of T7 RNAP or mutant, 20 ng/μl template, and DEPC-treated water. Reactions were incubated at 37°C for 1 h, mixed with an equivalent volume of RNA loading dye consisting of 95% formamide, 0.02% SDS, 0.02% bromophenol blue, 0.01% xylene cyanol, and 0.5 mM EDTA, and then electrophoresed on 2% agarose gels. Gels were stained with 1 μg/ml ethidium bromide and viewed on a UV transilluminator. Bands corresponding to transcription products were quantified with ImageJ software.

Model for range expansion with susceptible infected recovered kinetics

Based on classic assumptions (Kermack & McKendrick, 1927; Bailey, 1957; Anderson & May, 1991; Busenberg & Cooke, 1993; Capasso, 1993; Hethcote, 2000; Keeling & Rohani, 2007), the host population could be categorized into three classes: the susceptible cells S, the infective cells I, and the recovered cells R. Susceptible cells enter into the infective compartment after catching an illness or virus and then into the recovered class as a consequence of recovery. Presumably, an individual who recovers from the illness has perpetual immunity thereafter. The model on the bases of these hypotheses is referred to as the SIR model, which is a classical and simple model to explain the rapid rise and fall in the number of infected patients observed in epidemics such as the plague (London 1665–1666, Bombay 1906) and cholera (London 1865).

Unlike most phages, the infection of M13 phage is chronic. During the process, infected host cells are not killed, and progeny phages are continuously produced and extruded through the cell membrane as the infected cells continue to grow at a lowered rate (Marvin & Hohn, 1969; Smeal *et al*, 2017a, 2017b) until they become recovered (Appendix Fig S1). Recovered cells grow as fast as the susceptible ones, and they produce progeny phages at a much lower level as compared to the freshly infected cells (Appendix Fig S1). These features make it suitable to describe M13 phage infection process with a modified SIR model (Fig EV1, Appendix Fig S2). And different from models developed for other phages with a lytic life cycle, our model does not include a term of latent phase. This is because the initial period of ~10 min before the progeny phages start to be continuously produced (Marvin & Hohn, 1969; Ploss & Kuhn, 2010) is a negligible short time scale compared to the whole process of bacteria-phage copropagation (> 10 h) in our system.

For *E. coli* grown in semisolid agar plate, which allows the bacterial cells to swim, the spatiotemporal dynamics is governed by two elements: cell motility and cell growth, that is, diffusion, chemotaxis, and cell growth. To gain a quantitative insight on the spatial bacteria–phage copropagation process, we developed a RESIR (Range Expansion with Susceptible Infected Recovered kinetics) model based on navigated range expansion model of bacterial population (Cremer *et al*, 2019) and modified SIR model of phage infection. The motility and chemotaxis of bacteria are represented by the Keller-Segel-type diffusion and advective terms widely used in the literature (Keller & Segel, 1971a, 1971b; Fu *et al*, 2018; Cremer *et al*, 2019). The spatiotemporal dynamics of bacterial cell density (susceptible bacteria S, infected bacteria I, recovered bacteria R), concentrations of the main nutrient (n) and chemoattractant (a), and the number of M13 phage particles (p) are described as follows:

© 2022 The Authors

$$\frac{\partial S}{\partial t} = \mu\nabla^2 S - \chi\nabla\cdot(S\nabla f) + \lambda(n)S - \kappa(p)\lambda(n)pS \quad (1)$$

$$\frac{\partial I}{\partial t} = \mu\nabla^2 I - \chi\nabla\cdot(I\nabla f) + \eta_1\lambda(n)I + \kappa(p)\lambda(n)pS - \theta_1 I \quad (2)$$

$$\frac{\partial R}{\partial t} = \mu\nabla^2 R - \chi\nabla\cdot(R\nabla f) + \beta_1\lambda(n)R + \theta_1 I \quad (3)$$

$$\frac{\partial n}{\partial t} = D_n\nabla^2 n - \lambda(n)(S+I+R)/Y_n \quad (4)$$

$$\frac{\partial a}{\partial t} = D_a\nabla^2 a - \gamma(a)(S+I+R) \quad (5)$$

$$\frac{\partial p}{\partial t} = \alpha[(1-\eta_1)\lambda(n)I + (1-\beta_1)\lambda(n)R] \quad (6)$$

Herein, μ and χ are the effective diffusion and chemotactic coefficient of bacteria, respectively; D_n and D_a are diffusion coefficients of the nutrient and chemoattractant, respectively. η_1 and β_1 are the growth suspension rate of infected bacteria and recovered bacteria, θ_1 is the recovery rate of infected bacteria, α is the production rate of phage, Y_n is the yield nutrient consumption.

Chemotactic movement of bacteria depends on the concentration of the local attractant as follows (Liu *et al*, 2019):

$$f = \log\frac{1 + a/K_1}{1 + a/K_2} \quad (7)$$

where K_1 and K_2 are the lower and upper Weber offset of attractant sensing.

Bacterial cell growth is described with consideration of the growth limitations caused by nutrient availability (Murray, 2002), and follows a Monod relation

$$\lambda(n) = \frac{\lambda_0 n}{n + n_k} \quad (8)$$

with the Monod constant n_k and the growth rate λ_0.

Similarly, phage infection efficiency is described by a Monod relation

$$\kappa(p) = \frac{\kappa_0 p}{p + p_k} \quad (9)$$

with the Monod constant p_k and the phage infection efficiency κ_0.

In the same way, chemoattractant uptake is described by a Monod relation (Fu *et al*, 2018; Cremer *et al*, 2019):

$$\gamma(s) = \frac{g_0 a}{a + a_k} \quad (10)$$

with the Monod constant a_k and the uptake rate of chemoattractant g_0.

Simulations

The equations of the above bacteria–phage interaction models were integrated in MATLAB (R2019a) using second-order-centered differences for the spatial derivatives (mesh size 100 μm) and an explicit fourth-order Runge–Kutta routine for temporal integration (time step 1 s) in a Cartesian coordinate system. The parameters used in this study are summarized in Appendix Table S2.

Boundary conditions obey zero diffusive ($\partial_x\Phi = 0$, $\partial_y\Phi = 0$, where $\Phi = S, I, R, n, a, p$). The simulation starts with a locally restricted susceptible cell density S^{init}, in the radial distance r (r < 2 mm) from the center of the simulation region, $S^{init} = S_0\, e^{-\frac{r^2}{r_0^2}}$ ($s_0 = 0.2$ OD_{600} and $r_0 = 1$ mm), and the initial phage density at the inoculated site is $p^{init} = p_0\, e^{-\frac{r^2}{r_0^2}}$, where $r_0 = 1$ mm, and $p_0 = 1$ a.u. The initial nutrient and attractant concentrations are homogenously distributed with the concentration $n_0 = 30$ mM and $a_0 = 60$ μM.

Competition simulation

We further extended the RESIR model to the phage competitive model that can describe the competition between two phages, in which the phages were assumed to infect with different progeny phage production rates, despite the same infection efficiency. The phage competition infection efficiency is described as

$$\kappa(P) = \frac{\kappa_0(p_1 + p_2)}{(p_1 + p_2) + p_k} \quad (11)$$

The boundary conditions are the same as the former model, that is, all terms obey zero diffusive flux. The bacteria cell, nutrition, and chemoattractant initial conditions are the same as the former model, except for the phage. For initial conditions of the phage, the unequal (or equal) initial uniform mixture of two phages is located at the same position and the same initial density profile as the former model, that is, $p_1^{init} = p_0\, e^{-\frac{r^2}{r_0^2}}$ and $p_2^{init} = Cp_0\, e^{-\frac{r^2}{r_0^2}}$, C representing the ratio of strong and weak phages in the initial mixture.

We further considered the possibility that resistant bacterial cells immune to phage infection at all times exist in the population. Experimental results showed that ~90% of the bacterial cells exhibit fluorescence signals 10 h after infection by a reporter phage carrying red fluorescent protein gene in its genome, and this percentage could maintain over an extended culturing period (Appendix Fig S9A). Subsequent transfer of such preinfected (recovered) cells into fresh medium and inoculation of another reporter phage carrying green fluorescent protein gene did not produce any cells exhibiting green fluorescence signals (Appendix Fig S9B). These results suggested that at least 90% of bacterial cells were infected and then became resistant, and this percentage could be maintained over time. The rest 10% were possibly immune to phage infection for unknown reasons even before exposure to phages. Nevertheless, even when we assume that 10% of the bacterial cells are indeed resistant to phage infection at the beginning, the conclusions of the model simulated phage spatial competition are not changed (Appendix Fig S10).

Data availability

Major data for figures have been included in Source Data. Modeling and analysis code have been submitted to GitHub with a link https://github.com/YiZhangsiat/RESIR-model.

Expanded View for this article is available online.

Acknowledgements
The authors thank David R. Liu for insightful discussions and providing the plasmids which were used for the construction of accessory plasmid and mutagenesis plasmid. We thank Hui Wu for assistance with the protein analyses. This work was financially supported by Strategic Priority Research Program of Chinese Academy of Sciences (XDB29050500), National Key R&D Program of China (2018YFA0902700, 2018YFA0903600), National Natural Science Foundation of China (32025022, 32071417), Joint NSFC-ISF Research Grant (32061143021), CAS Interdisciplinary Innovation Team (Grant No. JCTD-2019-16), Shenzhen Science and Technology Innovation Committee (Grant No. JCYJ20180507182241844), Guangdong Provincial Key Laboratory of Synthetic Genomics (Grant No. 2019B030301006), and Guangdong Basic and Applied Basic Research Foundation (Grant No. 2020A1515110287).

Author contributions
Ting Wei: Data curation; investigation; methodology; writing – original draft; writing – review and editing. **Wangsheng Lai:** Data curation; investigation; methodology; writing – original draft. **Qian Chen:** Data curation; investigation; methodology; writing – original draft. **Yi Zhang:** Software; methodology; writing – original draft. **Chenjian Sun:** Data curation; investigation; writing – original draft. **Xionglei He:** Writing – review and editing. **Guoping Zhao:** Writing – review and editing. **Xiongfei Fu:** Software; supervision; methodology; writing – review and editing. **Chenli Liu:** Conceptualization; supervision; methodology; writing – original draft; project administration; writing – review and editing.

Disclosure and competing interests statement
The authors have filed a provisional patent application on the SPACE system and related improvements.

References

Agresti JJ, Antipov E, Abate AR, Ahn K, Rowat AC, Baret J-C, Marquez M, Klibanov AM, Griffiths AD, Weitz DA (2010) Ultrahigh-throughput screening in drop-based microfluidics for directed evolution. *Proc Natl Acad Sci USA* 107: 4004–4009

Aguirre-Liguori JA, Ramirez-Barahona S, Gaut BS (2021) The evolutionary genomics of species' responses to climate change. *Nat Ecol Evol* 5: 1350–1360

Altizer S, Bartel R, Han BA (2011) Animal migration and infectious disease risk. *Science* 331: 296–302

Anderson RM, May RM (1991) *Infectious diseases of humans: dynamics and control*. Oxford: Oxford University Press

Andow DA, Kareiva PM, Levin SA, Okubo A (1990) Spread of invading organisms. *Landsc Ecol* 4: 177–188

Badran AH, Liu DR (2015) Development of potent *in vivo* mutagenesis plasmids with broad mutational spectra. *Nat Commun* 6: 8425

Badran AH, Guzov VM, Huai Q, Kemp MM, Vishwanath P, Kain W, Nance AM, Evdokimov A, Moshiri F, Turner KH *et al* (2016) Continuous evolution of bacillus thuringiensis toxins overcomes insect resistance. *Nature* 533: 58–63

Bailey NTJ (1957) *The mathematical theory of epidemics*. London: Charles Griffin & Company

Baym M, Lieberman TD, Kelsic ED, Chait R, Gross R, Yelin I, Kishony R (2016) Spatiotemporal microbial evolution on antibiotic landscapes. *Science* 353: 1147–1151

Bloom JD, Arnold FH (2009) In the light of directed evolution: pathways of adaptive protein evolution. *Proc Natl Acad Sci USA* 106: 9995–10000

Blum TR, Liu H, Packer MS, Xiong X, Lee P-G, Zhang S, Richter M, Minasov G, Satchell KJF, Dong M *et al* (2021) Phage-assisted evolution of botulinum neurotoxin proteases with reprogrammed specificity. *Science* 371: 803–810

Bosshard L, Dupanloup I, Tenaillon O, Bruggmann R, Ackermann M, Peischl S, Excoffier L (2017) Accumulation of deleterious mutations during bacterial range expansions. *Genetics* 207: 669–684

Brissette JL, Russel M, Weiner L, Model P (1990) Phage shock protein, a stress protein of *Escherichia coli*. *Proc Natl Acad Sci USA* 87: 862–866

Brissette JL, Weiner L, Ripmaster TL, Model P (1991) Characterization and sequence of the *Escherichia coli* stress-induced psp operon. *J Mol Biol* 220: 35–48

Bryson DI, Fan C, Guo LT, Miller C, Soll D, Liu DR (2017) Continuous directed evolution of aminoacyl-tRNA synthetases. *Nat Chem Biol* 13: 1253–1260

Busenberg SN, Cooke KL (1993) *Vertically transmitted diseases: models and dynamics*. Berlin, Heidelberg: Springer

Capasso V (1993) *Mathematical structures of epidemic systems*. Berlin, Heidelberg: Springer

Carlson JC, Badran AH, Guggiana-Nilo DA, Liu DR (2014) Negative selection and stringency modulation in phage-assisted continuous evolution. *Nat Chem Biol* 10: 216–222

Chory EJ, Gretton DW, DeBenedictis EA, Esvelt KM (2021) Enabling high-throughput biology with flexible open-source automation. *Mol Syst Biol* 17: e9942

Cremer J, Honda T, Tang Y, Wong-Ng J, Vergassola M, Hwa T (2019) Chemotaxis as a navigation strategy to boost range expansion. *Nature* 575: 658–663

Crook N, Abatemarco J, Sun J, Wagner JM, Schmitz A, Alper HS (2016) *In vivo* continuous evolution of genes and pathways in yeast. *Nat Commun* 7: 13051

DeBenedictis EA, Chory EJ, Gretton DW, Wang B, Golas S, Esvelt KM (2022) Systematic molecular evolution enables robust biomolecule discovery. *Nat Methods* 19: 55–64

Deforet M, Carmona-Fontaine C, Korolev KS, Xavier JB (2019) Evolution at the edge of expanding populations. *Am Nat* 194: 291–305

Deng L-W, Perham RN (2002) Delineating the site of interaction on the pIII protein of filamentous bacteriophage fd with the F-pilus of *Escherichia coli*. *J Mol Biol* 319: 603–614

Dickinson BC, Packer MS, Badran AH, Liu DR (2014) A system for the continuous directed evolution of proteases rapidly reveals drug-resistance mutations. *Nat Commun* 5: 5352

d'Oelsnitz S, Ellington A (2018) Continuous directed evolution for strain and protein engineering. *Curr Opin Biotechnol* 53: 158–163

English JG, Olsen RHJ, Lansu K, Patel M, White K, Cockrell AS, Singh D, Strachan RT, Wacker D, Roth BL (2019) VEGAS as a platform for facile directed evolution in mammalian cells. *Cell* 178: 748–761

Esvelt KM, Carlson JC, Liu DR (2011) A system for the continuous directed evolution of biomolecules. *Nature* 472: 499–503

Excoffier L, Foll M, Petit RJ (2009) Genetic consequences of range expansions. *Annu Rev Ecol Evol Syst* 40: 481–501

Fallah-Araghi A, Baret J-C, Ryckelynck M, Griffiths AD (2012) A completely *in vitro* ultrahigh-throughput droplet-based microfluidic screening system for protein engineering and directed evolution. *Lab Chip* 12: 882–891

Fraebel DT, Mickalide H, Schnitkey D, Merritt J, Kuhlman TE, Kuehn S (2017) Environment determines evolutionary trajectory in a constrained phenotypic space. *Elife* 6: e24669

 © 2022 The Authors

Fronhofer EA, Altermatt F (2015) Eco-evolutionary feedbacks during experimental range expansions. *Nat Commun* 6: 6844

Fu X, Kato S, Long J, Mattingly HH, He C, Vural DC, Zucker SW, Emonet T (2018) Spatial self-organization resolves conflicts between individuality and collective migration. *Nat Commun* 9: 2177

Gandhi SR, Yurtsev EA, Korolev KS, Gore J (2016) Range expansions transition from pulled to pushed waves as growth becomes more cooperative in an experimental microbial population. *Proc Natl Acad Sci USA* 113: 6922–6927

Harms MJ, Thornton JW (2013) Evolutionary biochemistry: revealing the historical and physical causes of protein properties. *Nat Rev Genet* 14: 559–571

Hastings A, Cuddington K, Davies KF, Dugaw CJ, Elmendorf S, Freestone A, Harrison S, Holland M, Lambrinos J, Malvadkar U *et al* (2004) The spatial spread of invasions: new developments in theory and evidence. *Ecol Lett* 8: 91–101

Hethcote HW (2000) The mathematics of infectious diseases. *SIAM Rev* 42: 599–653

Hu JH, Miller SM, Geurts MH, Tang W, Chen L, Sun N, Zeina CM, Gao X, Rees HA, Lin Z *et al* (2018) Evolved Cas9 variants with broad PAM compatibility and high DNA specificity. *Nature* 556: 57–63

Hubbard BP, Badran AH, Zuris JA, Guilinger JP, Davis KM, Chen L, Tsai SQ, Sander JD, Joung JK, Liu DR (2015) Continuous directed evolution of DNA-binding proteins to improve TALEN specificity. *Nat Methods* 12: 939–942

Ikeda RA, Chang LL, Warshamana GS (1993) Selection and characterization of a mutant T7 RNA polymerase that recognizes an expanded range of T7 promoter-like sequences. *Biochemistry* 32: 9115–9124

Imburgio D, Rong M, Ma K, McAllister WT (2000) Studies of promoter recognition and start site selection by T7 RNA polymerase using a comprehensive collection of promoter variants. *Biochemistry* 39: 10419–10430

Jones RA (2009) Plant virus emergence and evolution: origins, new encounter scenarios, factors driving emergence, effects of changing world conditions, and prospects for control. *Virus Res* 141: 113–130

Kareinen L, Ogola J, Kivisto I, Smura T, Aaltonen K, Jaaskelainen AJ, Kibiwot S, Masika MM, Nyaga P, Mwaengo D *et al* (2020) Range expansion of bombali virus in mops condylurus bats, Kenya, 2019. *Emerg Infect Dis* 26: 3007–3010

Keeling MJ, Rohani P (2007) *Modeling infectious diseases in humans and animals*. Princeton, NJ: Princeton University Press

Kelehear C, Brown GP, Shine R (2012) Rapid evolution of parasite life history traits on an expanding range-edge. *Ecol Lett* 15: 329–337

Keller EF, Segel LA (1971a) Model for chemotaxis. *J Theor Biol* 30: 225–234

Keller EF, Segel LA (1971b) Traveling bands of chemotactic bacteria: a theoretical analysis. *J Theor Biol* 30: 235–248

Kermack WO, McKendrick AG (1927) A contribution to the mathematical theory of epidemics. *Proc R Soc Lond Ser A Contain Pap Math Phys Character* 115: 700–721

Koster DA, Mayo A, Bren A, Alon U (2012) Surface growth of a motile bacterial population resembles growth in a chemostat. *J Mol Biol* 424: 180–191

Li X, Gonzalez F, Esteves N, Scharf BE, Chen J (2020) Formation of phage lysis patterns and implications on co-propagation of phages and motile host bacteria. *PLoS Comput Biol* 16: e1007236

Liu W, Cremer J, Li D, Hwa T, Liu C (2019) An evolutionarily stable strategy to colonize spatially extended habitats. *Nature* 575: 664–668

Liu W, Tokuyasu TA, Fu X, Liu C (2021) The spatial organization of microbial communities during range expansion. *Curr Opin Microbiol* 63: 109–116

Marvin DA, Hohn B (1969) Filamentous bacterial viruses. *Bacteriol Rev* 33: 172–209

Messing J (1983) [2] New M13 vectors for cloning. In *Methods in Enzymology*, Wu R, Grossman L, Moldave K (eds), pp 20–78. San Diego, CA: Academic Press

Meyer AJ, Ellefson JW, Ellington AD (2015) Directed evolution of a panel of orthogonal T7 RNA polymerase variants for *in vivo* or *in vitro* synthetic circuitry. *ACS Synth Biol* 4: 1070–1076

Miller TEX, Angert AL, Brown CD, Lee-Yaw JA, Lewis M, Lutscher F, Marculis NG, Melbourne BA, Shaw AK, Szűcs M *et al* (2020) Eco-evolutionary dynamics of range expansion. *Ecology* 101: e03139

Morrison MS, Podracky CJ, Liu DR (2020) The developing toolkit of continuous directed evolution. *Nat Chem Biol* 16: 610–619

Murray JD (2002) *Mathematical biology I. An introduction*. New York, NY: Springer-Verlag

Ni B, Ghosh B, Paldy FS, Colin R, Heimerl T, Sourjik V (2017) Evolutionary remodeling of bacterial motility checkpoint control. *Cell Rep* 18: 866–877

Ochocki BM, Miller TE (2017) Rapid evolution of dispersal ability makes biological invasions faster and more variable. *Nat Commun* 8: 14315

Packer MS, Rees HA, Liu DR (2017) Phage-assisted continuous evolution of proteases with altered substrate specificity. *Nat Commun* 8: 956

Parmesan C, Yohe G (2003) A globally coherent fingerprint of climate change impacts across natural systems. *Nature* 421: 37–42

Pham HL, Wong A, Chua N, Teo WS, Yew WS, Chang MW (2017) Engineering a riboswitch-based genetic platform for the self-directed evolution of acid-tolerant phenotypes. *Nat Commun* 8: 411

Phillips BL, Perkins TA (2019) Spatial sorting as the spatial analogue of natural selection. *Theor Ecol* 12: 155–163

Piatkevich KD, Jung EE, Straub C, Linghu C, Park D, Suk H-J, Hochbaum DR, Goodwin D, Pnevmatikakis E, Pak N *et al* (2018) A robotic multidimensional directed evolution approach applied to fluorescent voltage reporters. *Nat Chem Biol* 14: 352–360

Ping D, Wang T, Fraebel DT, Maslov S, Sneppen K, Kuehn S (2020) Hitchhiking, collapse, and contingency in phage infections of migrating bacterial populations. *ISME J* 14: 2007–2018

Ploss M, Kuhn A (2010) Kinetics of filamentous phage assembly. *Phys Biol* 7: 045002

Rakonjac J, Model P (1998) Roles of pIII in filamentous phage assembly. *J Mol Biol* 282: 25–41

Ramirez KS, Snoek LB, Koorem K, Geisen S, Bloem LJ, Ten Hooven F, Kostenko O, Krigas N, Manrubia M, Cakovic D *et al* (2019) Range-expansion effects on the belowground plant microbiome. *Nat Ecol Evol* 3: 604–611

Raskin CA, Diaz GA, McAllister WT (1993) T7 RNA polymerase mutants with altered promoter specificities. *Proc Natl Acad Sci USA* 90: 3147–3151

Ravikumar A, Arzumanyan GA, Obadi MKA, Javanpour AA, Liu CC (2018) Scalable, continuous evolution of genes at mutation rates above genomic error thresholds. *Cell* 175: 1946–1957

Richter MF, Zhao KT, Eton E, Lapinaite A, Newby GA, Thuronyi BW, Wilson C, Koblan LW, Zeng J, Bauer DE *et al* (2020) Phage-assisted evolution of an adenine base editor with improved Cas domain compatibility and activity. *Nat Biotechnol* 38: 883–891

Rong M, He B, McAllister WT, Durbin RK (1998) Promoter specificity determinants of T7 RNA polymerase. *Proc Natl Acad Sci USA* 95: 515–519

Shih HY, Mickalide H, Fraebel DT, Goldenfeld N, Kuehn S (2018) Biophysical constraints determine the selection of phenotypic fluctuations during directed evolution. *Phys Biol* 15: 065003

Shine R, Brown GP, Phillips BL (2011) An evolutionary process that assembles phenotypes through space rather than through time. *Proc Natl Acad Sci USA* 108: 5708–5711

Smeal SW, Schmitt MA, Pereira RR, Prasad A, Fisk JD (2017a) Simulation of the M13 life cycle I: assembly of a genetically-structured deterministic chemical kinetic simulation. *Virology* 500: 259–274

Smeal SW, Schmitt MA, Pereira RR, Prasad A, Fisk JD (2017b) Simulation of the M13 life cycle II: investigation of the control mechanisms of M13 infection and establishment of the carrier state. *Virology* 500: 275–284

Tabor JJ (2012) Modular gene-circuit design takes two steps forward. *Nat Methods* 9: 1061–1063

Thuronyi BW, Koblan LW, Levy JM, Yeh WH, Zheng C, Newby GA, Wilson C, Bhaumik M, Shubina-Oleinik O, Holt JR *et al* (2019) Continuous evolution of base editors with expanded target compatibility and improved activity. *Nat Biotechnol* 37: 1091

Walther G-R, Post E, Convey P, Menzel A, Parmesan C, Beebee TJC, Fromentin J-M, Hoegh-Guldberg O, Bairlein F (2002) Ecological responses to recent climate change. *Nature* 416: 389–395

Wang T, Badran AH, Huang TP, Liu DR (2018) Continuous directed evolution of proteins with improved soluble expression. *Nat Chem Biol* 14: 972–980

Wong BG, Mancuso CP, Kiriakov S, Bashor CJ, Khalil AS (2018) Precise, automated control of conditions for high-throughput growth of yeast and bacteria with eVOLVER. *Nat Biotechnol* 36: 614–623

Wu H, Wei T, Yu B, Cheng R, Huang F, Lu X, Yan Y, Wang X, Liu C, Zhu B (2021) A single mutation attenuates both the transcription termination and RNA-dependent RNA polymerase activity of T7 RNA polymerase. *RNA Biol* 18: 451–466

License: This is an open access article under the terms of the Creative Commons Attribution License, which permits use, distribution and reproduction in any medium, provided the original work is properly cited.

 © 2022 The Authors

Expanded View Figures

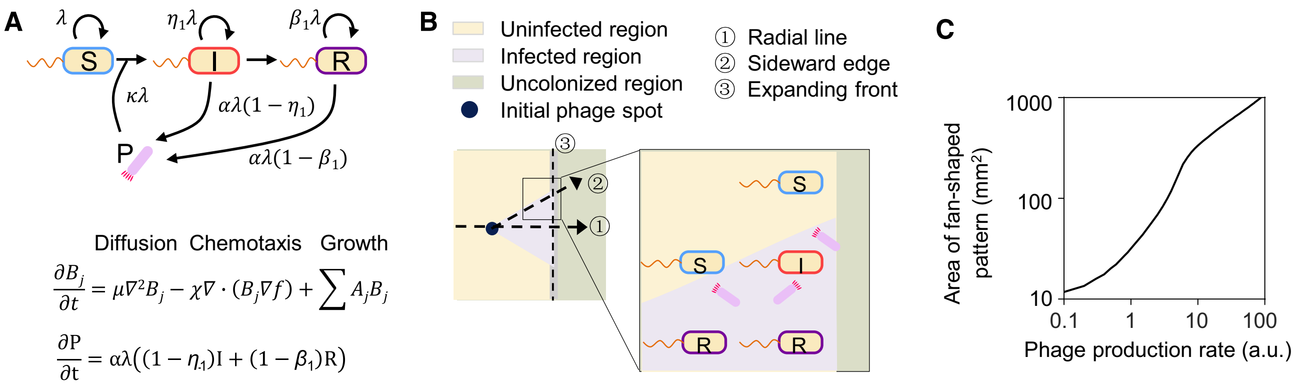

Figure EV1. Kinetic model of the interaction between bacteria and phage.

A The bacterial populations are classified into three categories: susceptible, infected, and recovered bacteria. Infected bacteria are converted from susceptible bacteria by phage infection, and eventually become recovered bacteria. These three bacterial populations all proliferate by consuming the nutrition in the semisolid culture media. In our experimental system, motile bacteria expand their range into unoccupied territories by diffusion and chemotaxis. In the meantime, the nonmotile phages are transmitted by their host bacteria, and their titer depends on the level of infectious progeny phage production by infected and recovered bacteria. The details of this model are described in Materials and Methods and Appendix Table S2.

B The spatiotemporal dynamics of phage infection in the radial direction (①) and the lateral direction (②) of the fan-shaped infection region are distinct from each other. In the radial direction, the expansion of infection range is driven by a "hitchhiking effect" (Ping *et al*, 2020), that is, phages are transported by the bacteria at the moving front. The bacterial population at the front experiences an active infection process involving the emergence of infected and recovered cells, the annihilation of susceptible cells, and eventually all cells become recovered cells, maintaining a balance between cell growth at the front and back diffusion (Cremer *et al*, 2019) (③). Differently, along the sideward edge, the expansion of infection range is driven by a "relay effect" of infected bacteria in the lateral direction, that is, infected bacteria from the infected region invade into the uninfected region, in which they produce phages continuously encountering and infecting susceptible bacterial cells. This cycle repeats and shows a relay-like effect, effectively generating a moving boundary between the infected and uninfected regions, which is eventually presented as the sideward edge (②).

C Model prediction showing that the size of the fan-shaped pattern is positively correlated with the phage production rate.

Source data are available online for this figure.

© 2022 The Authors

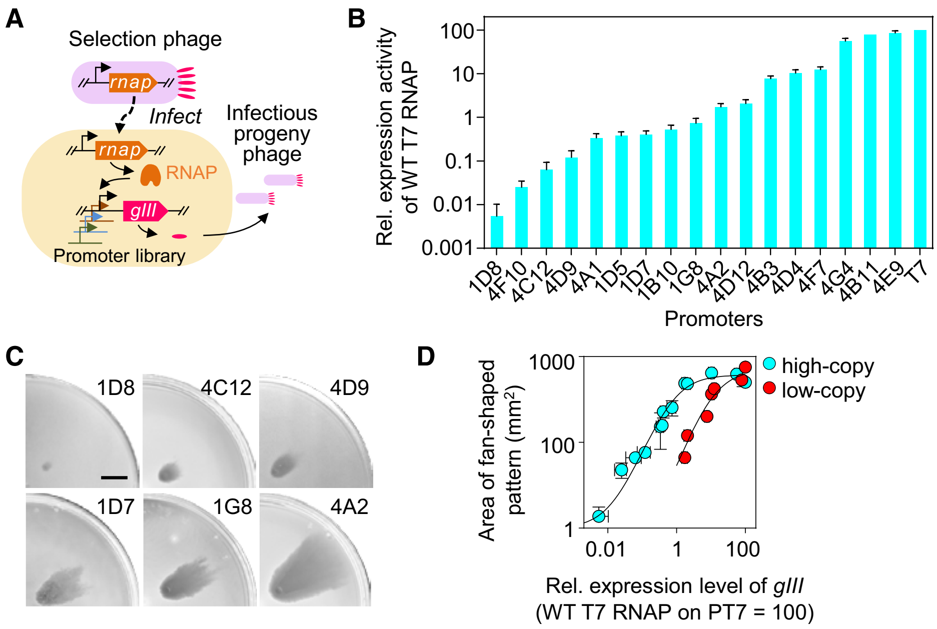

Figure EV2. Relationship between the area of the fan-shaped pattern and the *gIII* expression level.

A An activity-dependent phage propagation cassette on accessory plasmid. Expression level of *gIII* is under the control of a library of synthetic promoter variants. Selection phage carries a wild-type T7 RNA polymerase (RNAP) gene in place of its *gIII*. The T7 RNAP exhibits different transcriptional activities on different promoter variants.

B Relative expression levels of 17 synthetic promoter variants (sequences shown in Appendix Table S3) were determined by *in vivo* transcriptional activity assay (Materials and Methods). Error bars represent s.d. of three biologically independent assays.

C Photographs of a quarter of the semisolid agar plates with typical patterns obtained for bacteria carrying accessory plasmids containing representative promoters with different expression activities in (B). Scale bar represents 1 cm.

D Relationship between the area of the fan-shaped pattern and the *gIII* expression level. Data represent mean ± s.d. for at least three biological replicates. Fitting lines are generated with functions $y = 10 + 600x/(0.8 + x)$ and $y = 700x/(12 + x)$ for high- and low-copy accessory plasmids, respectively.

Source data are available online for this figure.

 © 2022 The Authors

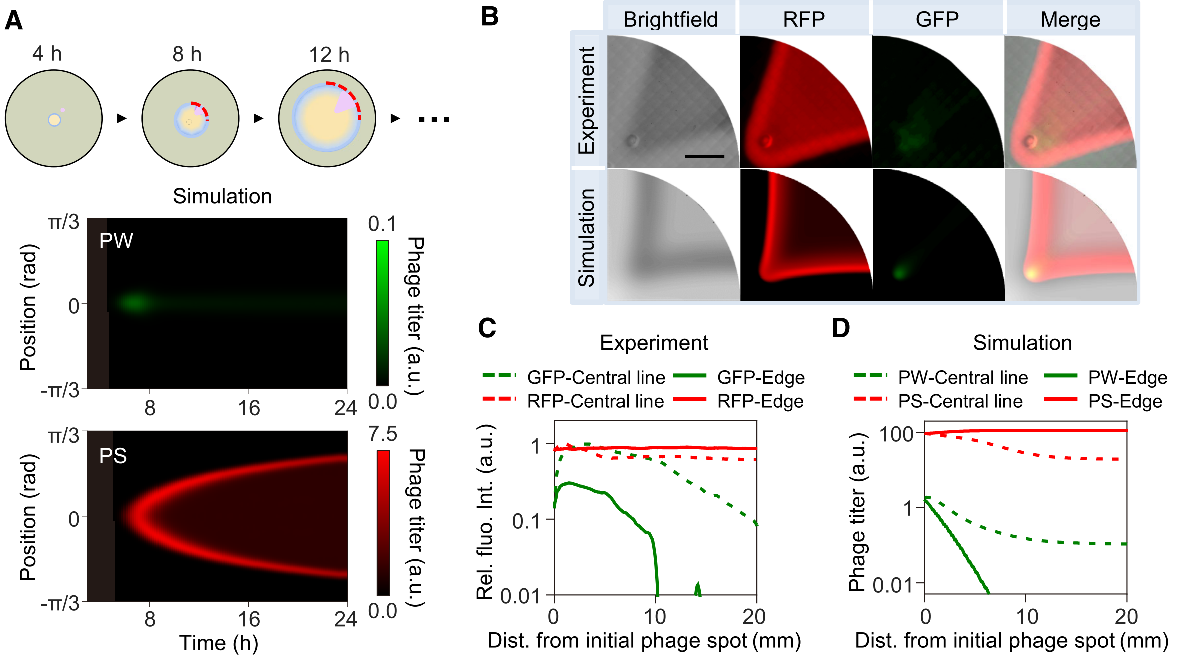

Figure EV3. Competition between weak and strong phages with an initial titer ratio of 1:1.

A Simulated kymograph of weak (PW) and strong (PS) phage titers in the bacterial expanding front as shown by the red dashed arc line in the schematic with an initial titer ratio of 1:1. The production rates of PW and PS are set as 40 and 100, respectively.

B The experimental result shows raw photographs of a representative two-phage competition assay after initial inoculation with 1:1 mixture of M13s and M13w at 1 cm away from the center. The simulation result is the outcome of competition between two phages with relative production rates as those in panel (A). Scale bar represents 1 cm.

C Profiles of the fluorescence intensity along the central radial line and the sideward edge of the fan-shaped infection zone in the experimental result in (B). The relative intensities were obtained by dividing the detected values with the maximum value of red or green fluorescence intensity, respectively.

D Plots of the simulated phage-titer profiles of PW and PS after 24-h competition.

Source data are available online for this figure.

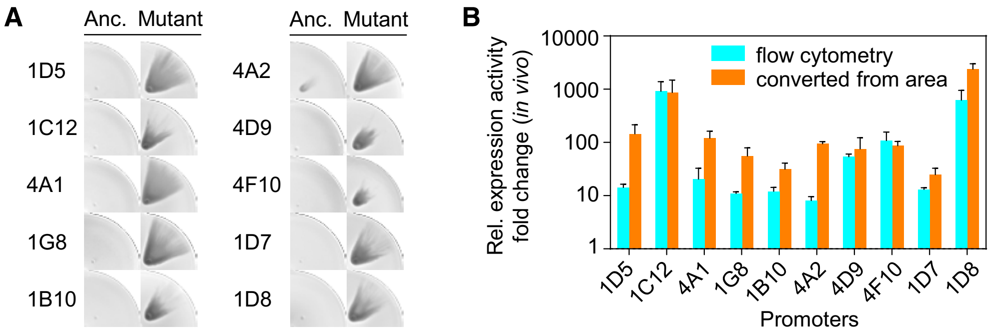

Figure EV4. Improved expression activity of RNAP mutants.

A Improvement in RNAP recognition of 10 selected synthetic promoters as shown in Fig 4B is demonstrated visually, by comparing the area size of fan shapes formed by phages carrying corresponding mutant RNAP genes with those carrying the wild-type T7 RNAP gene (Anc.). *Escherichia coli* FM15 cells carrying low-copy accessory plasmids were used. The images of a quarter of the agar plate containing a representative fan-shaped pattern are shown.

B Fold changes in the relative expression activity of RNAP mutants on their corresponding target promoters. The activity of the mutants was measured either by the *in vivo* transcriptional assay based on flow cytometry (Materials and Methods) or converted from the area of fan-shaped pattern using the transfer function in Fig EV2D. Data represent mean values ± s.d. for three biological replicates.

Source data are available online for this figure.

© 2022 The Authors

Exploiting spatial dimensions to enable parallelized continuous directed evolution

Ting Wei[1,*], Wangsheng Lai[1,*], Qian Chen[1,2,*], Yi Zhang[1,*], Chenjian Sun[1,2], Xionglei He[3], Guoping Zhao[1,4], Xiongfei Fu[1,2,+], Chenli Liu[1,2,+]

[1]CAS Key Laboratory for Quantitative Engineering Biology, Shenzhen Institute of Synthetic Biology, Shenzhen Institutes of Advanced Technology, Chinese Academy of Sciences, Shenzhen 518055, China
[2]University of Chinese Academy of Sciences, Beijing 100049, China
[3]State Key Laboratory of Biocontrol, School of Life Sciences, Sun Yat-Sen University, Guangzhou, 510275, China
[4]Key Laboratory of Synthetic Biology, Institute of Plant Physiology and Ecology, Shanghai Institutes for Biological Sciences, Chinese Academy of Sciences, Shanghai, 200032, China

*equal contribution
[+]To whom correspondence may be addressed. Email: cl.liu@siat.ac.cn or xiongfei.fu@siat.ac.cn

Appendix

Appendix Text (page 3)

Appendix Text S1. Experimental notes
Calculation of phage generation time of SPACE system

Appendix Figures

Appendix Figure S1 (page 4). Growth curves of *E. coli* and phage production after infection.
Appendix Figure S2 (page 5). Model simulation of phage infection process.
Appendix Figure S3 (page 6). Time-lapse plots of the simulated relative bacterial cell-density and nutrient profiles along an arc line across the infected region with a fixed radius.
Appendix Figure S4 (page 7). Competition between weak and strong phages with an initial titer ratio of 10^5:1.
Appendix Figure S5 (page 8). Gating strategy used for flow cytometry analysis.
Appendix Figure S6 (page 9-12). Histograms obtained for the measurement of *in vivo* transcriptional activity based on flow cytometry.
Appendix Figure S7 (page 13). Enrichment of PS by SPACE system and liquid continuous culturing system by model simulation.
Appendix Figure S8 (page 14). Simulated competition of five phage strains with different bacterial expansion speed.
Appendix Figure S9 (page 15). Ratio of bacterial cells with fluorescent signals after infection by reporter phages.
Appendix Figure S10 (page 16). Simulated competition of two phages with a host bacterial population containing resistant cells.

Appendix Tables

Appendix Table S1 (page 17). Bacterial strains used in this study.
Appendix Table S2 (page 17-18). Model parameters.
Appendix Table S3 (page 18-20). Sequences of T7 promoter variants.
Appendix Table S4 (page 20-21). Plasmids used in this study.
Appendix Table S5 (page 21). Representative mutants obtained from parallelized SPACE of T7 RNAP

Appendix References (page 21)

Appendix Text S1. Experimental notes

Calculation of phage generation time of SPACE system

We estimated the phage generation time using the same model proposed for PACE (Esvelt *et al*, 2011). This model is based on an assumption that lagoons, in which the phage-assisted evolution takes place, operate in a steady state that the total phage and cell concentration does not change over time. The average phage generation time in this model is equal to 2 / (dilution rate) (Esvelt *et al.*, 2011). In SPACE, the bacterial propagating front composed of exponentially growing and steadily advancing cells provides a constant flow of fresh host. Once infected by the phages, bacterial cells are able to migrate and carrying along progeny phages. It is analogous to a lagoon. Those left behind the propagating front are considered as outflow of the lagoon. Therefore, the dilution rate in SPACE system should be equal to (cell expansion speed /front width). The cell expansion speed measured from time-lapsed images (**Fig 1C**) is approximately 3.89 mm/h, and the front width is 1.10-1.41 mm, so the dilution rate of SPACE under this condition is 2.76-3.54 h^{-1}, comparable with the dilution rate of PACE (2.5-3.2 h^{-1}) (Esvelt *et al.*, 2011). In the meantime, the effective duration of the evolution in a single run is calculated by dividing the distance from the phage inoculation spot (10 mm from the center) to the edge of the agar plate with cell expansion speed, which is 8.35 h for an 8.5-cm plate, and 16.71 h for a 15-cm plate. Thus, a single run using an 8.5-cm agar plate corresponds to approximately 12-15 phage generations. Longer effective duration provided by a 15-cm agar plate allows 23-30 generations in a single run over night. More phage generations could be achieved by simply transferring to a new agar plate and start another run.

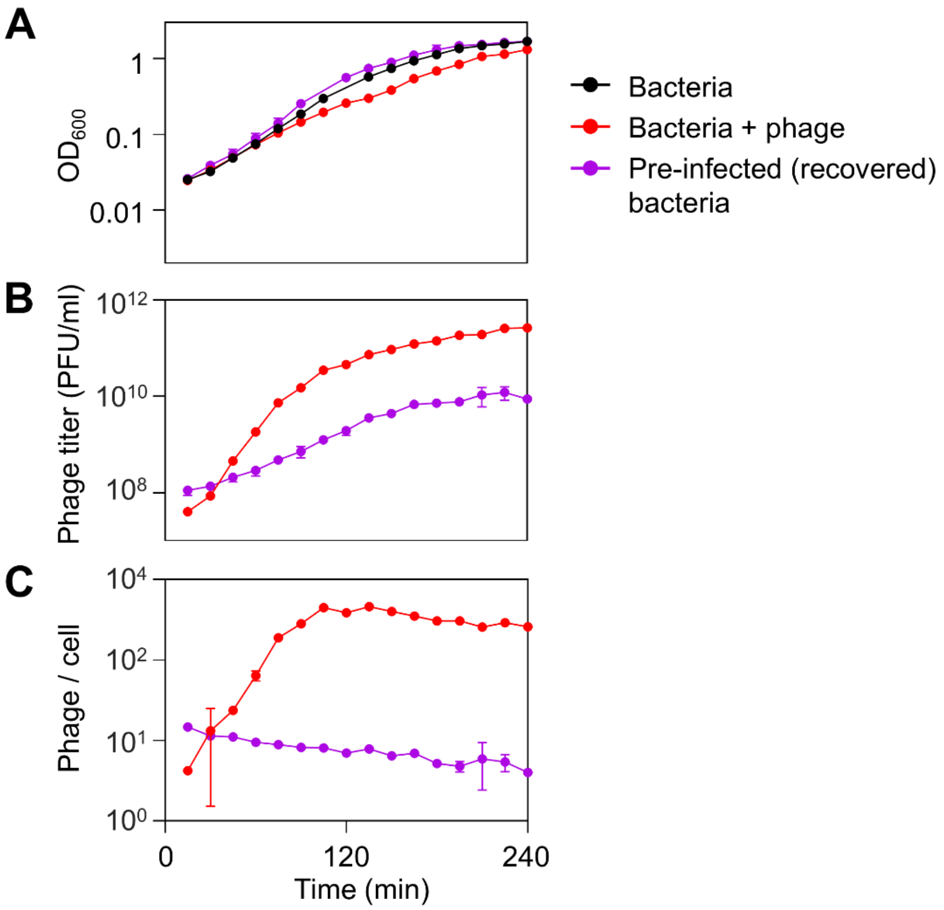

Appendix Figure S1 - Growth curves of *E. coli* and phage production after infection.

A Growth curves of uninfected, freshly infected, and pre-infected (recovered) bacteria. *E. coli* FM15 cells were cultured in LB broth. Bacteria are FM15 cells without phage inoculation; Bacteria + phage are cells with 10^8 PFU/ml of M13 phages added at the 0 time point. Pre-infected (recovered) bacteria are cells co-cultured overnight with phages, washed to remove phages in the supernatant, and transferred to fresh LB broth. For all three groups, the initial OD_{600} of the bacterial suspension was adjusted to 0.01.

B Phage titer in the supernatant of collected samples quantified by qPCR.

C Phage number per cell calculated by dividing phage titer with the cell density quantified by flow cytometry. Data represent mean values ± s.d. for three biological replicates.

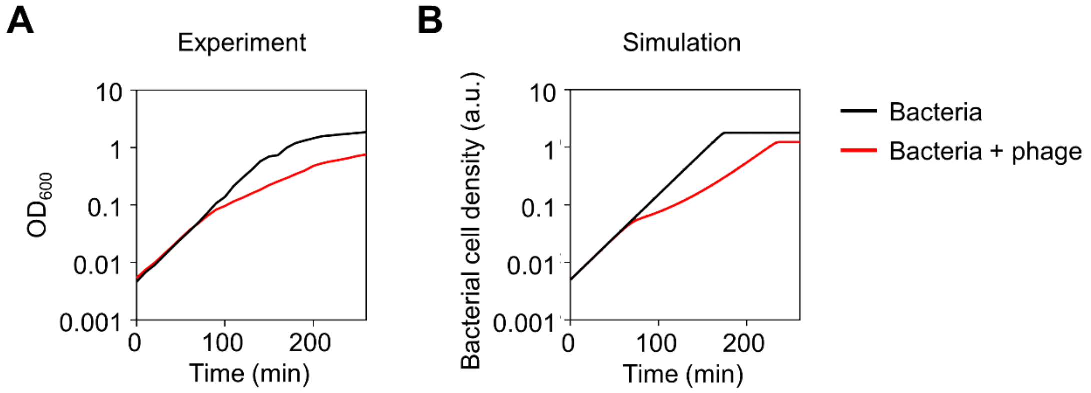

Appendix Figure S2 - **Model simulation of phage infection process.**

A Growth curves of *E. coli* FM15 cells in liquid culture without phage, or with 10^7 PFU/ml phages added at the beginning of the experiment.

B Model simulated bacterial growth curve without phage, or with an initial phage titer of 0.01 a.u. in a well-mixed system.

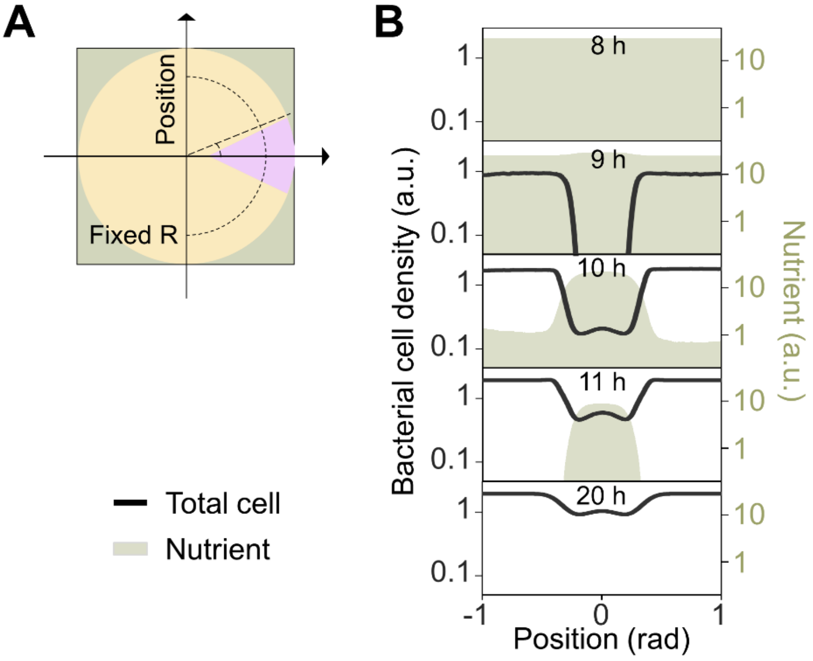

Appendix Figure S3 - **Time-lapse plots of the simulated relative bacterial cell-density and nutrient profiles along an arc line across the infected region with a fixed radius**.

A Schematic of a simulated fan shape formed after 20 h of bacterial range expansion. Yellow filled circle represents bacterial lawn. The fan-shaped infected zone is signified by purple color.

B Bacterial cell density and nutrient concentration values obtained from an arc line with a fixed radius across the fan shape as shown in (A). The model parameters used were identical to those used in the bottom two rows of Fig 1C. The fixed radius was set at 25 mm from the center of the agar plate.

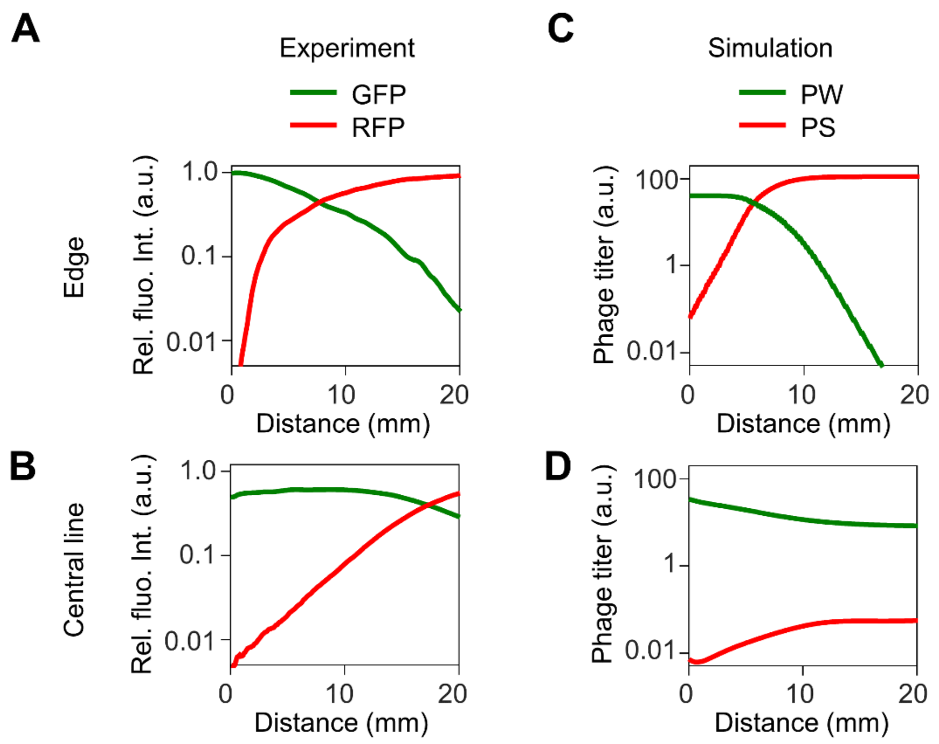

Appendix Figure S4 - Competition between weak and strong phages with an initial titer ratio of 10^5:1.

A, B Profiles of the fluorescence intensity along the central line and the edge of the fan-shaped infection zone in the experimental result in Fig 3C. The relative intensities were obtained by dividing the detected values with the maximum value of red or green fluorescence intensity, respectively.

C, D Simulated profiles of phage titer of the weak phage (PW) and the strong phage (PS) along the central line and the edge of the fan-shaped infection zone after competition as shown in Fig 3C.

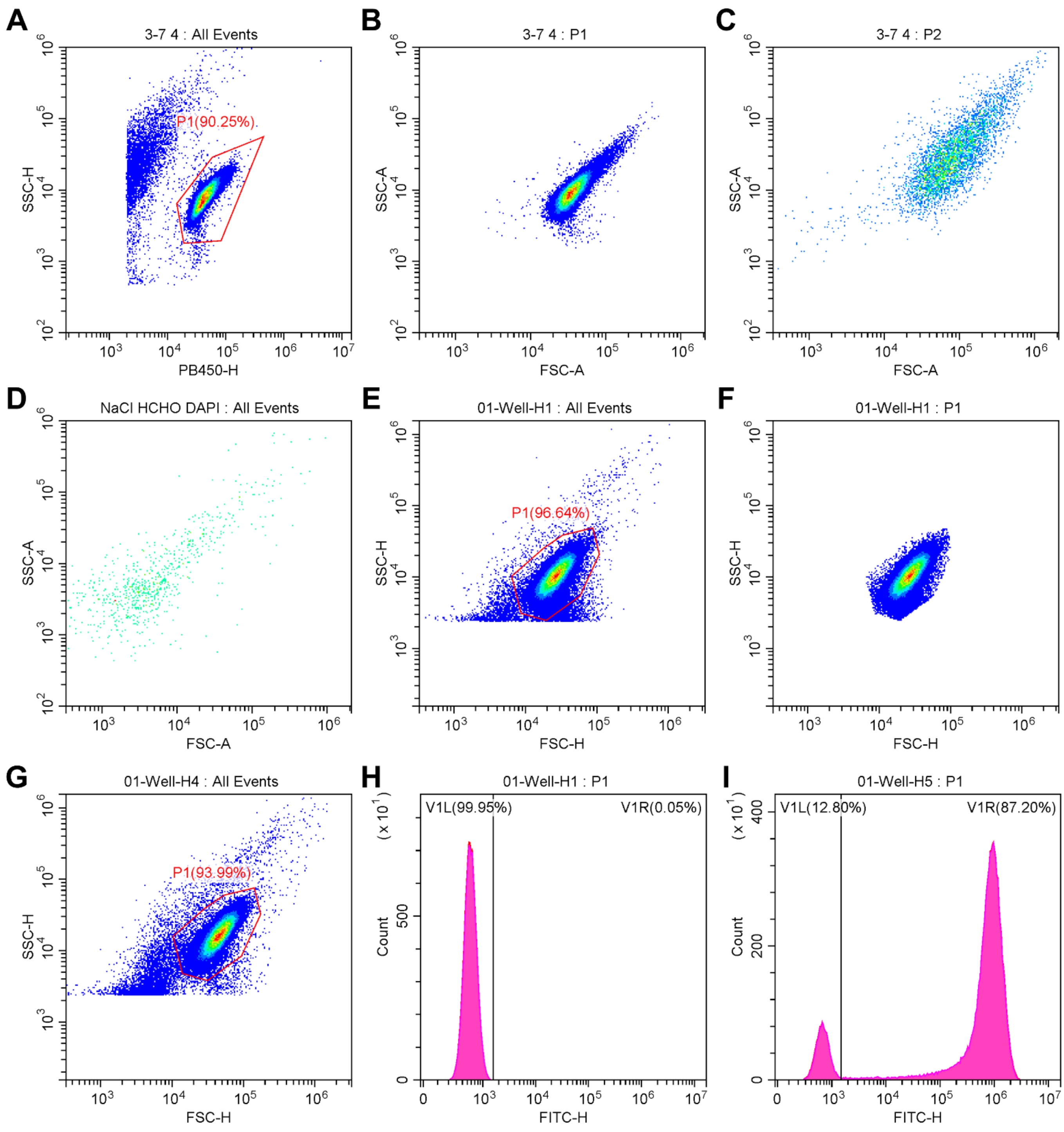

Appendix Figure S5 - Gating strategy used in flow cytometry analysis.

A-D Gating strategy used in cell counting. The DAPI-positive particles in P1 in panel (A) were regarded as bacterial cells. The FSC-H/SSC-H plots of populations inside and outside of P1 are shown in panels (B) and (C), respectively. The FSC-H/SSC-H plot of the staining buffer (0.9 % NaCl with 1 µg/ml DAPI) is shown in panel (D).

E-I Gating strategy used in the measurement of *in vivo* transcriptional activity. Particles in P1 in panel (E) were regarded as bacterial cells, and the population formed by these particles is shown in panel (F). The FSC-H/SSC-H plot of phage-infected bacterial cells is shown in panel (G). (H) For the measurement of *in vivo* transcriptional activity, V1L covering 99.95% of the population in an uninfected bacterial sample based on the distribution of FITC-H signal values was set to determine the background fluorescence. This gate was applied to phage-infected samples of the same bacterium, and particles falling in V1R as shown in panel (I) were used to measure the transcriptional activity.

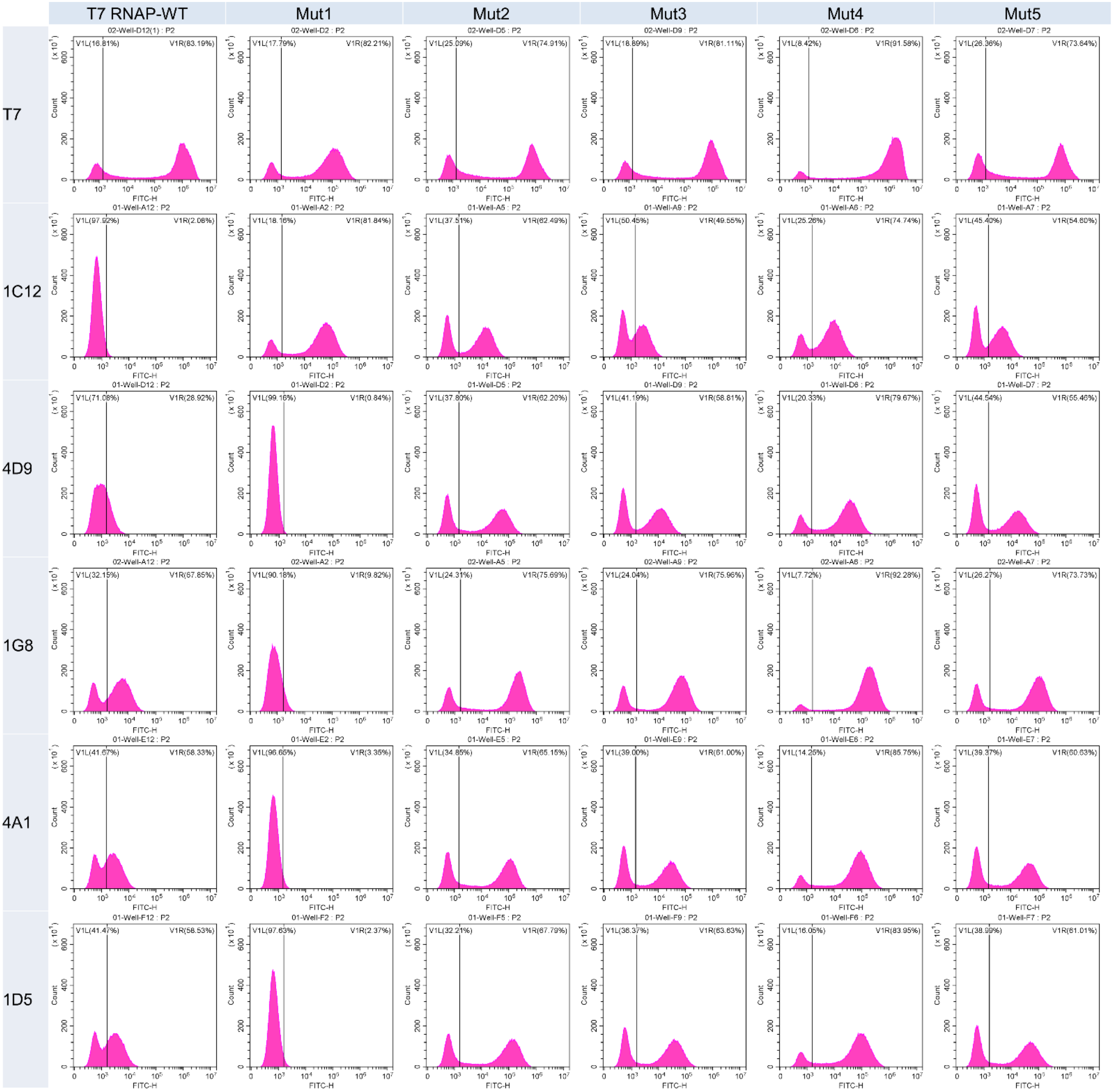

T7 RNAP-WT
Mut1
Mut2
Mut3
Mut4
Mut5
T7
1C12
4D9
1G8
4A1
1D5
FITC-H
Count

(Fig S6, Part 1)

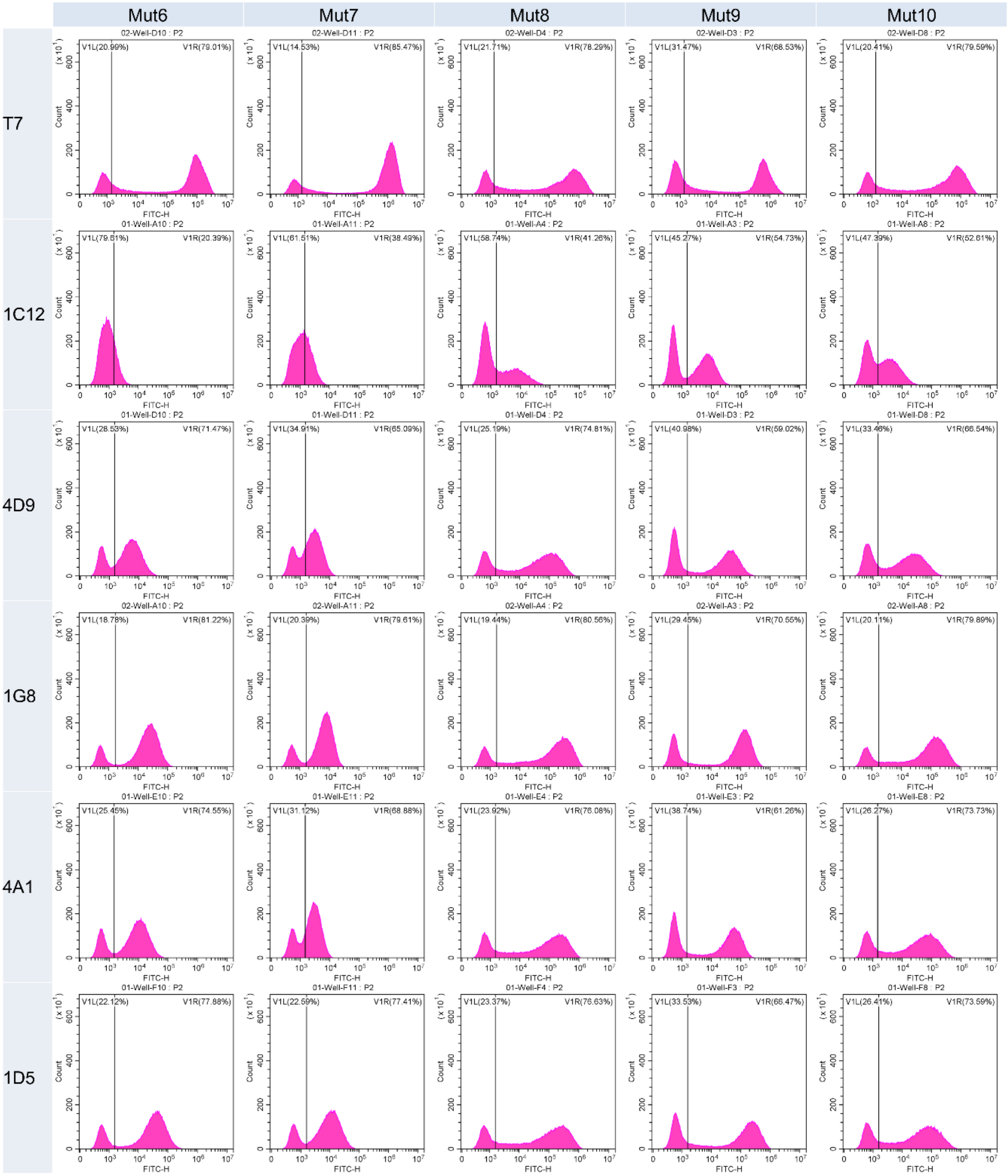

Mut6
Mut7
Mut8
Mut9
Mut10
T7
1C12
4D9
1G8
4A1
1D5
02-Well-D10 : P2
V1L(20.99%) V1R(79.01%)
02-Well-D11 : P2
V1L(14.53%) V1R(85.47%)
02-Well-D4 : P2
V1L(21.71%) V1R(78.29%)
02-Well-D3 : P2
V1L(31.47%) V1R(68.53%)
02-Well-D8 : P2
V1L(20.41%) V1R(79.59%)
01-Well-A10 : P2
V1L(79.61%) V1R(20.39%)
01-Well-A11 : P2
V1L(61.51%) V1R(38.49%)
01-Well-A4 : P2
V1L(58.74%) V1R(41.26%)
01-Well-A3 : P2
V1L(45.27%) V1R(54.73%)
01-Well-A8 : P2
V1L(47.39%) V1R(52.61%)
01-Well-D10 : P2
V1L(28.53%) V1R(71.47%)
01-Well-D11 : P2
V1L(34.91%) V1R(65.09%)
01-Well-D4 : P2
V1L(25.19%) V1R(74.81%)
01-Well-D3 : P2
V1L(40.98%) V1R(59.02%)
01-Well-D8 : P2
V1L(33.46%) V1R(66.54%)
02-Well-A10 : P2
V1L(18.78%) V1R(81.22%)
02-Well-A11 : P2
V1L(20.39%) V1R(79.61%)
02-Well-A4 : P2
V1L(19.44%) V1R(80.56%)
02-Well-A3 : P2
V1L(29.45%) V1R(70.55%)
02-Well-A8 : P2
V1L(20.11%) V1R(79.89%)
01-Well-E10 : P2
V1L(25.45%) V1R(74.55%)
01-Well-E11 : P2
V1L(31.12%) V1R(68.88%)
01-Well-E4 : P2
V1L(23.92%) V1R(76.08%)
01-Well-E3 : P2
V1L(38.74%) V1R(61.26%)
01-Well-E8 : P2
V1L(26.27%) V1R(73.73%)
01-Well-F10 : P2
V1L(22.12%) V1R(77.88%)
01-Well-F11 : P2
V1L(22.59%) V1R(77.41%)
01-Well-F4 : P2
V1L(23.37%) V1R(76.63%)
01-Well-F3 : P2
V1L(33.53%) V1R(66.47%)
01-Well-F8 : P2
V1L(26.41%) V1R(73.59%)
Count
FITC-H

(Fig S6, Part 2)

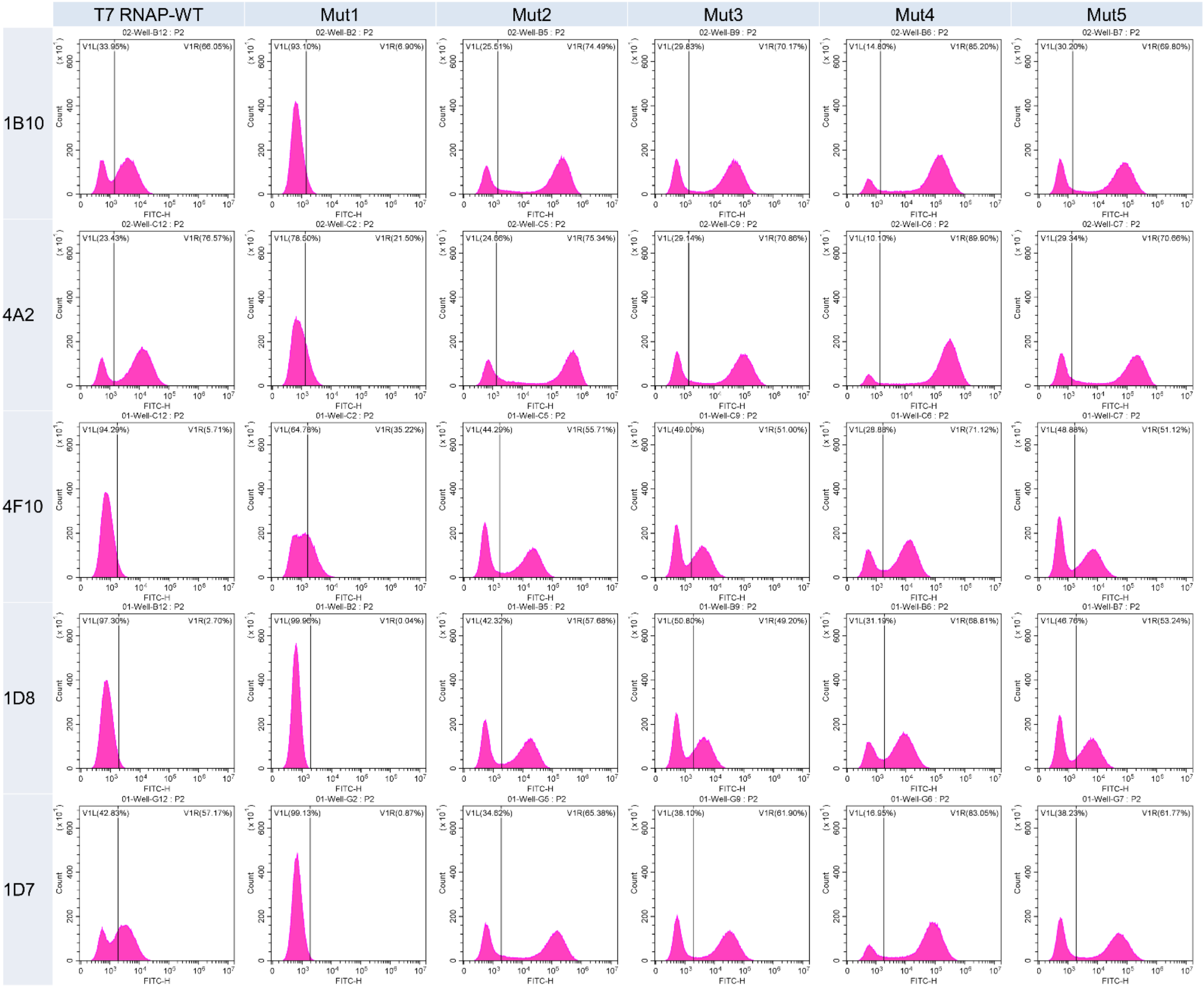
T7 RNAP-WT
Mut1
Mut2
Mut3
Mut4
Mut5
1B10
4A2
4F10
1D8
1D7
Count
FITC-H

(Fig S6, Part 3)

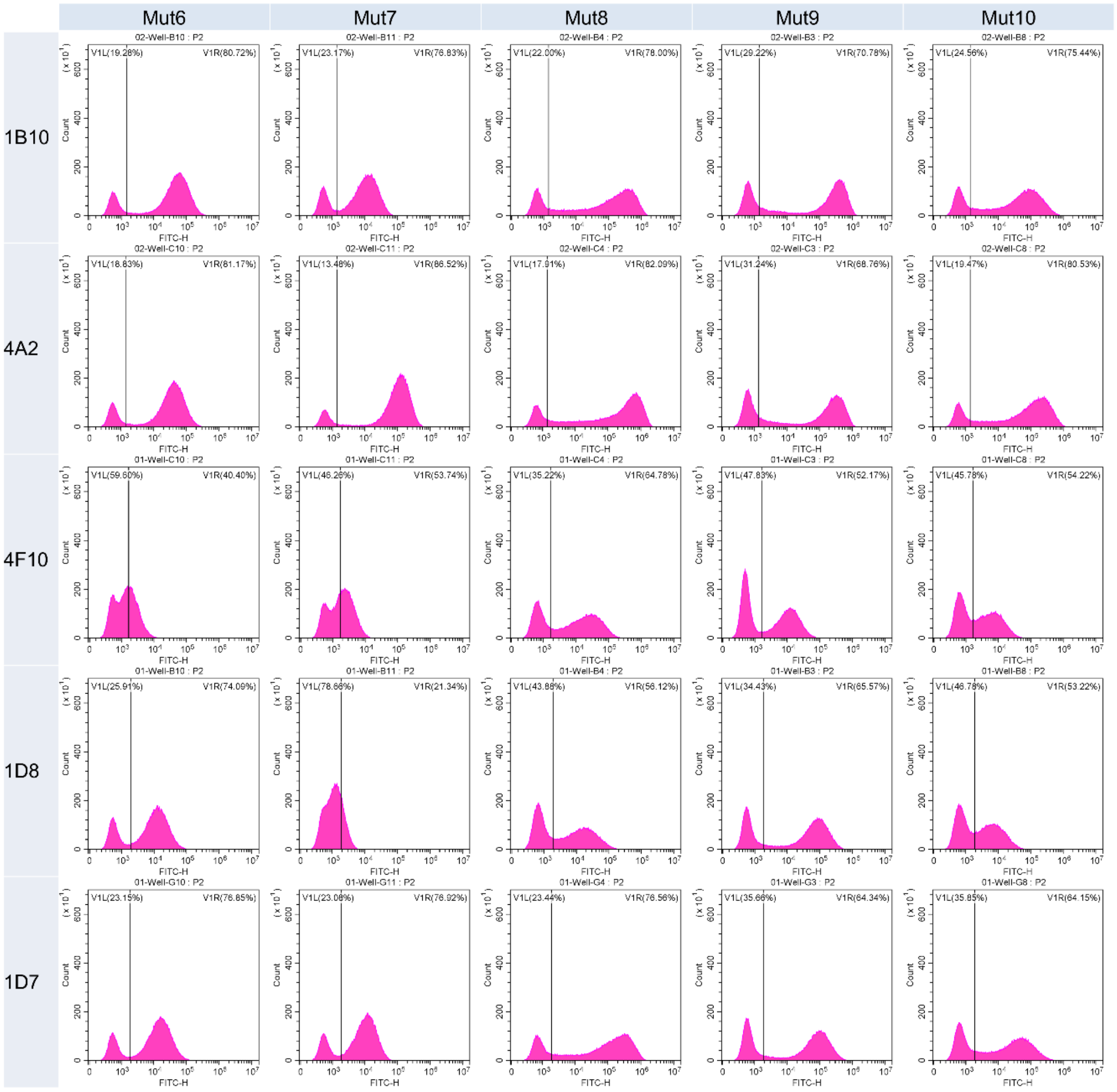

(Fig S6, Part 4)

Appendix Figure S6 - Histograms obtained for the measurement of *in vivo* transcriptional activity based on flow cytometry.

Results from one out of three replicated assays to measure the expression activity of the wild-type and 10 mutant RNAPs on T7 promoter and 10 promoter variants in **Fig 4D** are shown in this figure. Results from the other two replicates are included in the Source Data.

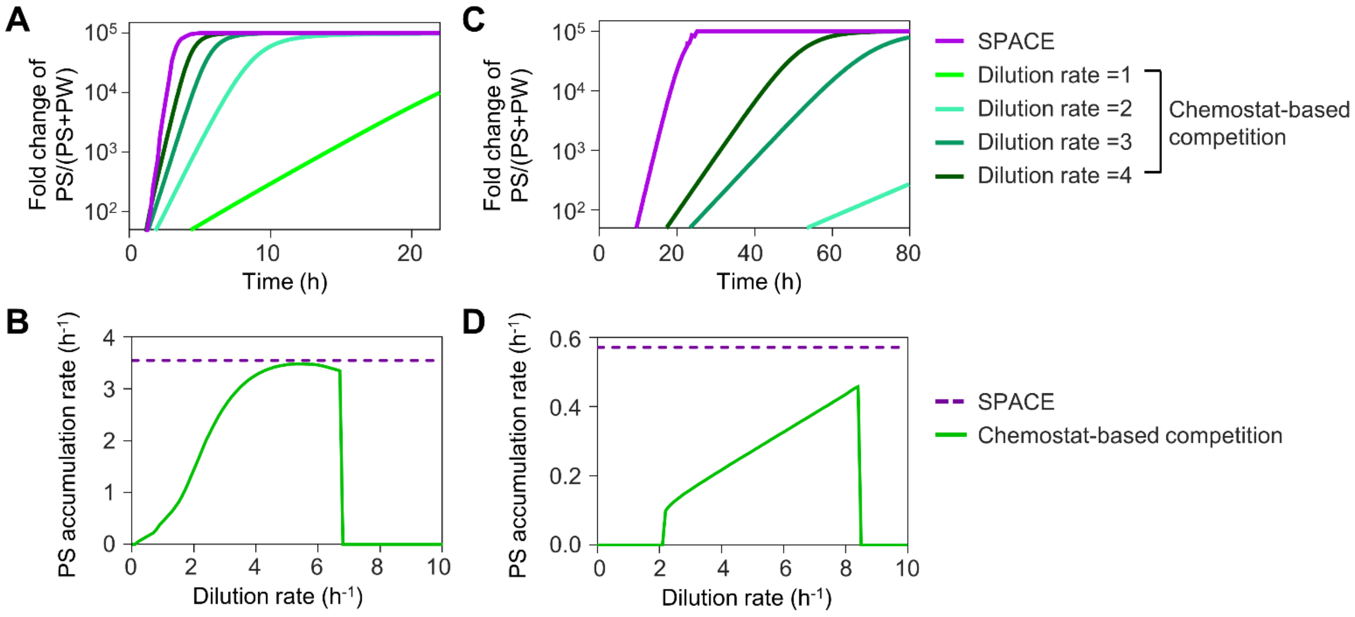

Appendix Figure S7 - Enrichment of PS by SPACE system and chemostat-based competition system by model simulation.

A, B Simulated fold change of PS/(PS+PW) and PS accumulation rate in SPACE and a chemostat-based competition system with a significant difference in the strength of PS and PW. Initial PW: PS is 10^5:1. Production rates of PW and PS are 40 and 100, respectively. Fold change along the sideward edge of infection zone under a SPACE condition the same as in **Fig 3** is compared with that in well-mixed chemostat-based competition system using different dilution rate (DR, volumes of the liquid system exchanged by continuous flow per hour). Data for liquid system in (B) are obtained by calculating the slope of curves on logarithmic scale with varied DR as shown in (A).

C, D Simulated fold change of PS/(PS+PW) and PS accumulation rate in SPACE and a chemostat-based competition system with a mild difference in the strength of PS and PW. Initial PW: PS is 10^5:1. Production rates of PW and PS are 90 and 100, respectively.

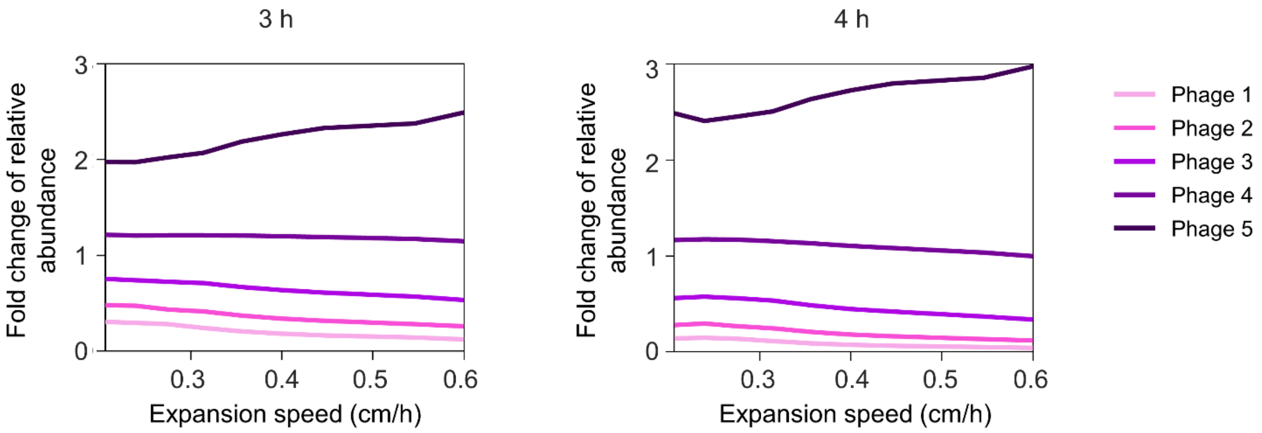

Appendix Figure S8 - Simulated competition of five phage strains with different bacterial expansion speed. Production rates of Phage 1-5 are 80, 85, 90, 95, and 100, respective. The initial mixture of phage inoculant consists of the five strains, each composing 20% of the whole population. Graphs show the fold change of the relative abundance of each phage at the bacterial expanding front near the edge as compared to that at the initial phage spot 3 h and 4 h after phage infection.

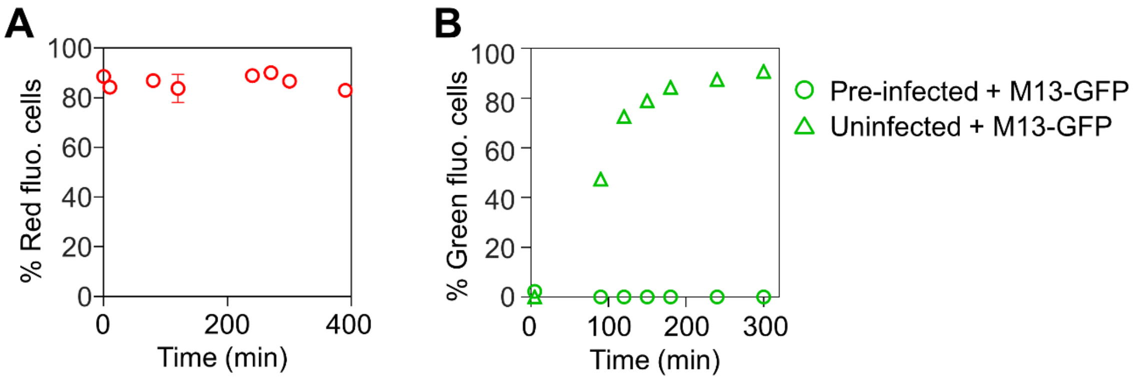

Appendix Figure S9 - Ratio of bacterial cells with fluorescent signals after infection by reporter phages.

A Time course of the percentage of cells exhibiting red fluorescence signals after overnight pre-infection by marker phages. *E. coli* FM15 cells in LB broth were infected by M13s used in **Fig 3**, the reporter M13 phage with a red fluorescent protein gene in its genome, for 10 hours. These pre-infected cells were washed to remove supernatant phages, and 1:500 transferred to fresh LB broth to continue culturing with fluorescence measurements at different time points.

B Time course of the percentage of cells exhibiting green fluorescence signals after M13-GFP infection of pre-infected and uninfected fresh bacterial cells. Pre-infected cells were prepared as in (A).

The ratio of fluorescent cells was measured by flow cytometry using PE and FITC channels for red and green fluorescence, respectively. Data represent mean values ± s.d. for three biological replicates.

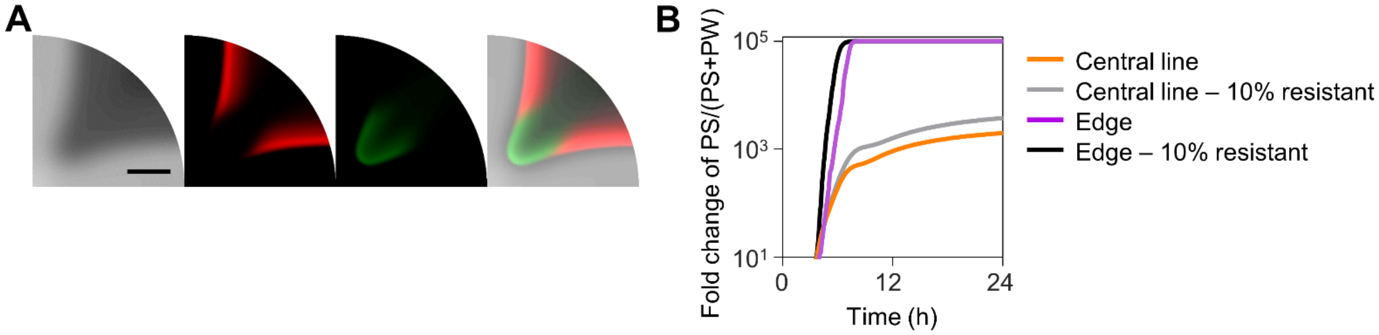

Appendix Figure S10 - Simulated competition of two phages with a host bacterial population containing resistant cells.

A, B The outcome and fold enrichment of the strong phage in simulated competition assay with an initial of weak phage (PW): strong phage (PS) at 10^5:1. The production rates of PW and PS are 40 and 100, respectively. In the host bacterial population, 10% of cells resistant to phage infection all the time was assumed for this simulation. In panel (B), the fold enrichment along the central radial line and sideward edge of infection zone is compared between two cases: bacterial population containing 0%, or 10% of cells resistant to phage infection.

Appendix Table S1. Bacterial strains used in this study.

Strain name	Genotype and remarks	Reference or source
E. coli MG1655-mCherry	*F⁻ λ– ilvG– rfb-50 rph-1 attB::mCherry (AmpR)*	Ref. (Liu *et al*, 2019)
E. coli ER2738	*F' proA+B+ lacI^q Δ(lacZ)M15 zzf::Tn10 (TetR)/fhuA2 glnV Δ(lac-proAB) thi-1 Δ(hsdS-mcrB)5*	NEB
E. coli DH5α	*F⁻ φ80d lacZΔM15 Δ(lacZYA-argF) U169 endA1 recA1 hsdR17(r_k^-, m_k^+) supE44λ- thi-1 gyrA96 relA1 phoA*	TransGen Biotech, Beijing
E. coli BL21	*F⁻ dcm omp*T *hsd*S(r_B^- m_B^-) *gal* [*mal*B$^+$]$_{K-12}$($λ^S$)	TransGen Biotech, Beijing
E. coli FMG1655-mCherry	*F' proA+B+ lacI^q Δ(lacZ)M15 zzf::Tn10 (TetR)/ λ– ilvG– rfb-50 rph-1 attB::mCherry (*Amp^R*)*	This study
E. coli FMG1655-mCherry *Δ(lacZ)M15*	*F' proA+B+ lacI^q Δ(lacZ)M15 zzf::Tn10 (TetR)/ λ– ilvG– rfb-50 rph-1 attB::mCherry (*Amp^R*) Δ(lacZ)M15*	This study
E. coli FM15	*F' proA+B+ lacI^q Δ(lacZ)M15 zzf::Tn10 (TetR)/ λ– ilvG– rfb-50 rph-1 attB::Kan^R Δ(lacZ)M15*	This study

Appendix Table S2. Model parameter

Parameter	Symbol	Value	Reference or parameter variation
Effective diffusion coefficient	μ	60 μm²/s	Ref. (Liu *et al.*, 2019)
Chemotactic coefficient	χ	410 μm²/s	Fit to match expansion speed for expansion in reference condition.
Diffusion of nutrient	D_n	800 μm²/s	Ref. (Cremer *et al*, 2016)
Diffusion of attractant	D_s	800 μm²/s	Ref. (Cremer *et al.*, 2016)
Growth rate	$λ_0$	2.08 h^{-1}	Average growth rate in LB medium. **Appendix Figure S2**
Yield nutrient consumption	Y_n	0.064 OD_{600}/mM	Ref. (Liu *et al.*, 2019)
Monod constant nutrient uptake	n_k	0.4 mM	Defined by fitting the growth curve of *E. coli*. **Appendix Figure S2**
Lower Weber offset attractant sensing	K_1	3.5 μM	Ref. (Yang *et al*, 2015)
Upper Weber offset attractant sensing	K_2	1000 μM	Ref. (Yang *et al.*, 2015)
Uptake rate chemoattractant	g_0	9 μM/(min • OD_{600})	Ref. (Fu *et al*, 2018)
Monod constant attractant uptake	s_k	1 μM	Ref. (Schellenberg & Furlong, 1977)
Phage production rate	α	0.1~100 a.u. /OD_{600}	Production rate varied to match the resultant fan shape size at different levels of *gIII* expression

Infection ability of phage	k_0	0.9 a.u. $^{-1}$	Fit to match the growth curve of *E. coli* infected by M13 bacteriophage. **Appendix Figure S2**
Monod constant phage uptake	p_k	0.01 a.u.	Fit to match the growth curve of *E. coli* infected by M13 bacteriophage. **Appendix Figure S2**
Growth coefficient of infected cell	η_1	0.1	Fit to match the growth curve of *E. coli* infected by M13 bacteriophage. **Appendix Figure S2**
Growth coefficient of recovered cell	β_1	0.9	Defined by fitting the growth curve of *E. coli* infected by M13 bacteriophage. **Appendix Figure S2**
Recovered coefficient of infected cell	θ_1	10^{-4} s^{-1}	Fit to match the growth curve of *E. coli* infected by M13 bacteriophage. **Appendix Figure S2**
Initial concentration of nutrients	n_0	30 mM	
Initial concentration of attractant	s_0	60 µM	

Appendix Table S3. Sequences of T7 promoter variants.

Number	Sequence
1A1	ACCACAGTGAACCTACAGGGAGA
1A2	TAATACGAGTCCCTACAGGGAGA
1A3	TCATACGAGTACCTACAGGGAGA
1A4	ATCGGAGTAAACGAGAAGGGAGA
1A5	AACTACTCCTTTCTAGCGGGAGA
1A6	GTTCGGGATTATGAGCCGGGAGA
1A7	GAATGCGACTCACTATAGGGAGA
1A8	TAATCCGACACACCATAGGGAGA
1A9	GGAGTATGATCTCCAAAGGGAGA
1A10	ACGCAGAACAGTACTAGGGGAGA
1A11	CCTAGTCTTTACGAGATGGGAGA
1A12	AAGTACCCTTTACCCTTGGGAGA
1B1	TAAGACGAAACACGATTGGGAGA
1B2	TAATACGACGCACGATAGGGAGA
1B3	TAATACGTAGGACGATAGGGAGA
1B4	TAATACGTAGGACGAAAGGGAGA
1B5	TAATAAGCCTCTCATCGGGGAGA
1B6	AAACGCCACTCACTGTAGGGAGA
1B7	AAGCGGCACTCAATGAAGGGAGA
1B8	TAATCCCTCCTACAATAGGGAGA
1B9	GGGAGAATTAACGGAAGGGGAGA
1B10	GAATGCAACTCAGGAAAGGGAGA
1B11	AGTAAACCTCGTTACACGGGAGA

1B12	GTTAGAGCAACCATTCTGGGAGA
1C7	TAGTACGCCCCACTATAGGGAGA
1C8	ACATACGATTCAAGCGCGGGAGA
1C9	AGGACTCTGCAGTACCTGGGAGA
1C10	TTCGACGTCCCACAAAGGGGAGA
1C11	ATGAAGCGTCCTGATCAGGGAGA
1C12	TAATACGACCCACTTCAGGGAGA
1D1	CTAGAATTCGGTTGACAGGGAGA
1D2	CAAGACTTCTGACGATAGGGAGA
1D3	TGAAGCAGCTTCGTACAGGGAGA
1D4	CGGGGTATTAAGTAGGCGGGAGA
1D5	TAGTGCGACTCACAATAGGGAGA
1D6	TGGTGCGACTAACAATAGGGAGA
1D7	TGTAAGAACTCACTTTGGGGAGA
1D8	TGTATGAACTCACGTTGGGGAGA
1D9	GCTGACGACTGACTATTGGGAGA
1D10	GTATAAGTATTCGCGCAGGGAGA
1D11	TTAGCCACTTGGAACTAGGGAGA
1D12	AAATACGACTCACTATGGGGAGA
1E1	GAAGACGTCTCACTATAGGGAGA
1E2	GCTAGGATTACGCTATAGGGAGA
1E3	GCTAGGATTACGCTATCGGGAGA
1E4	GCTAGGATTAAGATAACGGGAGA
1E5	TTCCACTTTGCCCGTTTGGGAGA
1E6	TTCCGATTTGACCGTTTGGGAGA
1E7	TAACACTTCTCACGATAGGGAGA
1E8	CAATGCTCCTCACTAAAGGGAGA
1E9	GAATACGACTTACTAGGGGGAGA
1E10	GAATCCGACTTACTAGGGGGAGA
1E11	TCACACGACACCCTATAGGGAGA
1E12	ATCTCCGGTCATACGATGGGAGA
1G1	TCTACGGAATGTCCATTGGGAGA
1G2	ATGATTCCGCGGTAGCAGGGAGA
1G3	TAGTACGCGTGACTATAGGGAGA
1G4	ATTCTGTTCGTGATTCGGGGAGA
1G5	CTTCGTGTCGGATTAAGGGGAGA
1G6	TGTACCCCTTAAACTCTGGGAGA
1G7	TGTACCCCTGAAACTCTGGGAGA
1G8	TAATACGCCTCACCACAGGGAGA
1G9	AATTGAACCTCACCGTGGGGAGA
1G10	CGTGTATGTGATACTGCGGGAGA
4A1	TAATACGCCTCACCACTGGGAGA
4A2	GAATGCGACTCCCTATAGGGAGA
4A3	AAATACTAACGATTATAGGGAGA
4A4	TAGGACGTAAGCCGAATGGGAGA
4A5	GCTAGGATTAAGCTAACGGGAGA
4A6	GTTTACGATTCTGAACCGGGAGA

4D7	AAGTACCCCTTACCCTTGGGAGA
4D8	AAGCAGAACTCTCCAAAGGGAGA
4D9	AAATAAGCCTCACCGTAGGGAGA
4D10	AACGACGACTCCGTGAAGGGAGA
4D11	ATGCAGTGGCTTAGAGTGGGAGA
4D12	TAATACGCGTCACTATAGGGAGA
4E1	TACTACGCCTCTCTAGCGGGAGA
4E2	AATCCAGATTGACCACTGGGAGA
4E3	GGCACGATAATGTGCGGGGGAGA
4E4	TCAAACGACTTACCGTAGGGAGA
4E5	CTAGCCACTGGGAACTAGGGAGA
4E6	AAGTTCACCCCTTTGATGGGAGA
4F1	GATTACGATTCTGAACCGGGAGA
4F2	TAAAGTCGATCAGTACTGGGAGA
4F3	ATGCCGATAGATGACCTGGGAGA
4F4	TAAAGTGGATCACTACTGGGAGA
4F5	AGTACTCCTGACACTCTGGGAGA
4F6	TAATGCGCCTGACAATAGGGAGA
4F7	TATGACGACTCACTATAGGGAGA
4F8	AGAGATCACGCAATCCAGGGAGA
4F9	ACTAAGAGCATCGGTAAGGGAGA
4F10	TAAAGTGGCTCACTATTGGGAGA
4F11	ATTCTCTTCGCGATTCGGGGAGA
4F12	CTAGCTTACGGTACTTAGGGAGA
4G1	GTATAAGACTCACCGCAGGGAGA
4G2	ATGATTCCGCGATAGTAGGGAGA

Appendix Table S4. Plasmids used in this study.

Plasmid name	Class	Antibiotic resistance	Origin of replication	Promoter	Genes
pLAa1~96	AP[a]	Spe	pUC	P_{T7} and variants	gIII
pLAasc1~21	AP	Carb	SC101	P_{T7} and variants	gIII
pLAa188a	AP	Carb	SC101	P_{psp}	gIII
pLM1	MP-s[b]	Chl	CloDF13	P_{psp}	pspABCDE-dnaQ926-dam-seqA
pLRp	RP[c]	Chl	CloDF13	P_{psp}	pspABCDE-sfGFP
pLAR1	ARP[d]	Spe	ColEI	P_{psp}	pspABCDE
pLAr1~21	RP	Carb	SC101	P_{T7} and variants	sfGFP
SP-T7	SP[e]		f1	P_{gIII}	T7 RNAP WT
SP-T7mut	SP		f1	P_{gIII}	T7 RNAP mutant

SP-T7GFP	SP	f1	P_{gIII}/J23100	T7 RNAP WT/sfGFP
SP-T7mutRuby	SP	f1	P_{gIII}/J23100	T7 RNAP mutant/mRuby

[a] accessory plasmid; [b] mutagenesis plasmid using psp promoter to drive mutator genes; [c] reporter plasmid; [d] accessory plasmid harboring regulator genes of psp promoter; [e] selection phage

Appendix Table S5. Representative mutants obtained from parallelized SPACE of T7 RNAP

Mutant no.	Amino acid changes
**Mut1**	E222G Q758R H772R
**Mut2**	E222K
**Mut3**	H772R
**Mut4**	E222A
**Mut5**	E222G
**Mut6**	K206R I244V
**Mut7**	E242K
**Mut8**	E222V D770N
**Mut9**	K206R E222K
**Mut10**	I244V

Appendix References

Cremer J, Segota I, Yang C-y, Arnoldini M, Sauls JT, Zhang Z, Gutierrez E, Groisman A, Hwa T (2016) Effect of flow and peristaltic mixing on bacterial growth in a gut-like channel. *Proceedings of the National Academy of Sciences* 113: 11414

Esvelt KM, Carlson JC, Liu DR (2011) A system for the continuous directed evolution of biomolecules. *Nature* 472: 499-503

Fu X, Kato S, Long J, Mattingly HH, He C, Vural DC, Zucker SW, Emonet T (2018) Spatial self-organization resolves conflicts between individuality and collective migration. *Nature communications* 9: 2177

Liu W, Cremer J, Li D, Hwa T, Liu C (2019) An evolutionarily stable strategy to colonize spatially extended habitats. *Nature* 575: 664-668

Schellenberg GD, Furlong CE (1977) Resolution of the multiplicity of the glutamate and aspartate transport systems of Escherichia coli. *The Journal of biological chemistry* 252: 9055-9064

Yang Y, M Pollard A, Höfler C, Poschet G, Wirtz M, Hell R, Sourjik V (2015) Relation between chemotaxis and consumption of amino acids in bacteria. *Molecular microbiology* 96: 1272-1282
